# Rheostats and Toggle Switches for Modulating Protein Function

**DOI:** 10.1371/journal.pone.0083502

**Published:** 2013-12-30

**Authors:** Sarah Meinhardt, Michael W. Manley, Daniel J. Parente, Liskin Swint-Kruse

**Affiliations:** Department of Biochemistry and Molecular Biology, The University of Kansas Medical Center, Kansas City, Kansas, United States of America; Oak Ridge National Laboratory, United States of America

## Abstract

The millions of protein sequences generated by genomics are expected to transform protein engineering and personalized medicine. To achieve these goals, tools for predicting outcomes of amino acid changes must be improved. Currently, advances are hampered by insufficient experimental data about nonconserved amino acid positions. Since the property “nonconserved” is identified using a sequence alignment, we designed experiments to recapitulate that context: Mutagenesis and functional characterization was carried out in 15 LacI/GalR homologs (rows) at 12 nonconserved positions (columns). Multiple substitutions were made at each position, to reveal how various amino acids of a nonconserved column were tolerated in each protein row. Results showed that amino acid preferences of nonconserved positions were highly context-dependent, had few correlations with physico-chemical similarities, and were not predictable from their occurrence in natural LacI/GalR sequences. Further, unlike the “toggle switch” behaviors of conserved positions, substitutions at nonconserved positions could be rank-ordered to show a “rheostatic”, progressive effect on function that spanned several orders of magnitude. Comparisons to various sequence analyses suggested that conserved and strongly co-evolving positions act as functional toggles, whereas other important, nonconserved positions serve as rheostats for modifying protein function. Both the presence of rheostat positions and the sequence analysis strategy appear to be generalizable to other protein families and should be considered when engineering protein modifications or predicting the impact of protein polymorphisms.

## Introduction

With the explosion of genomic sequencing, multiple sequence alignments (MSAs) of protein families are widely used to predict the functions of novel sequences, identify sites for mutagenesis, and predict the outcomes of polymorphisms [1]–[8]. To those ends, MSA analyses reliably identify conserved amino acid positions, which confer common overall structure and function to homologous proteins. Mutations at conserved positions usually impair function or destabilize structure. In addition, some nonconserved positions are important for protein function. For example, protein paralogs evolve functional variation *via* changes at important, nonconserved sites. In efforts to extract information about important, nonconserved positions from protein families, dozens of MSA analyses have been developed ([9]–[16] and many others).

In following this field, we were struck by the need for experimental studies explicitly designed to benchmark MSA analyses. In particular, we noted (i) that little information is available for nonconserved positions (most laboratory mutations are generated at conserved positions [2]) and (ii) the need for parallel mutagenesis in multiple protein homologs. Parallel mutagenesis recapitulates features of an MSA analysis, which uses the sequences of many homologs (rows) to predict family-wide properties of a given position (column). By making multiple substitutions at each position, the experimental data reveal how various amino acids of the column are tolerated in each protein (row) tested. This strategy also avoids an assumption that arises when only one homolog is used to predict family-wide behavior: “Mutational outcomes in one protein will be similar in other homologs.” Although this has been observed for many conserved positions, the assumption might *not* apply to nonconserved positions.

To implement parallel mutagenesis, we had to overcome two obstacles. First, parallel mutagenesis is hindered by the practical difficulty of performing hundreds of mutations on multiple proteins. Thus, the chosen protein function must be amenable to high-throughput assays. A bigger challenge arises in data interpretation: Each natural homolog may bind a different ligand; thus, the functional outcome of a mutation can be due to either the difference between proteins, between ligands, or both.

**Figure 1:**
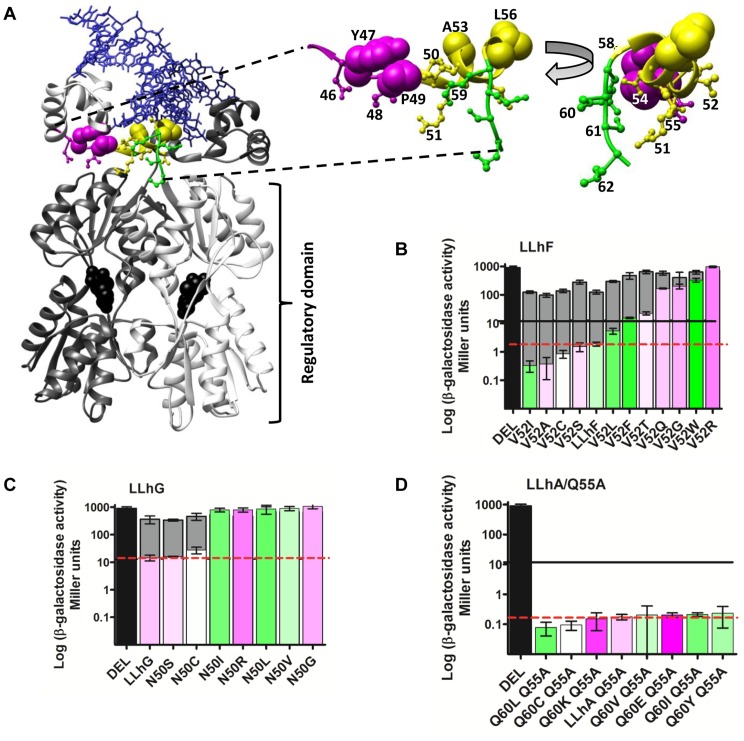
Structure and function of the LacI/GalR proteins. (A) Structure of LacI/GalR homodimer (pdb 1efa; [39]). One monomer is shown in white and the other in gray. DNA is shown with blue wires. The linker region is magenta (positions 45–49), yellow (50–58), and green (59–62). The YPAL motif is in space-filling; positions mutated in this study are in ball-and-sticks. Black space-filling shows an allosteric effector bound to the regulatory domain. On the right, the linker is enlarged and shown in two different views. (B–D) Representative functional data for LacI/GalR synthetic homologs. Repression of the *lac* operon was determined in the absence (front series) and presence (back series) of allosteric effector. Lower values correspond to tighter repression. “DEL” (black bar) shows β-galactosidase activity in the absence of repressor protein. Below 13 Miller units (solid black line), any change in repression impacted bacterial growth [17]. The red dashed lines indicate the activities of the starting proteins that are listed in Table 1. Error bars are the standard deviation of 2–4 independent bacterial colonies, each in quadruplicate or duplicate. Color coding of the front series represents amino acid hydrophobicity (green to magenta represents highest to lowest hydrophobicity); note the poor correlation with repression. Other physico-chemical scales are listed in Table S20 in Data S2 and mapped to repression data in Figures S25–S87 in Data S5, Data S6, and Data S7. (B) Rheostat example. (C) Toggle-like example (note that the red line overlaps the black line in this example). (D) Neutral example.

To overcome these difficulties, we created a family of synthetic paralogs using members of the LacI/GalR family of transcription regulators [17]–[19]. Using these proteins, the experimental hurdle was overcome by high-throughput mutagenesis and *in vivo* measurements of transcription regulation. The barrier to data interpretation was overcome by creating synthetic paralogs *via* domain recombination: The LacI/GalR proteins comprise a DNA binding domain linked to a regulatory domain by 18 amino acids (Figure 1A). Within this linker, several important nonconserved amino acids form an interface with nonconserved amino acids of the regulatory domain [20]. Synthetic “LXhX” paralogs were created by fusing the LacI DNA binding domain to various linkers and regulatory domains (Table 1). The resulting linker interface differed for each chimera but all bound the same DNA ligand [17]. Thus, when nonconserved linker positions are mutated, functional differences reflect only the differences between regulatory domains.

**Table 1:** Wild-type LacI/GalR proteins used to create the LXhX^a^ chimeras.

Natural Proteins^b^	“X” abbreviation	Mutated Proteins^c^	Repression (Miller units)^d^
LacI	L	LacI-11^e^	0.12±0.06
RbsR	R	LLhR	0.06±0.06
FruR	F	LLhF	1.9±0.3
GalR	G	LLhG	15±4
		LLhG/E62K	0.7±0.2
		LGhG	13±11
GalS	S	LLhS	6±4
		LLhS/R51S	58±20
		LLhS/D62N	3±1
		LLhS/R51S/D62N	0.06±0.03
PurR	P	LLhP	3.9±2.2
		LPhP57cs	37±5
		LGhP	320±130
TreR	T	LLh	120±16
		LLhT/V52A	0.5±0.1
AscG	A	LLhA	78±10
		LLhA/Q55A	0.2±0.04

a: Nomenclature: “L” indicates the LacI DNA binding domain (positions 1–44), “Xh” represents the protein source of the linker (positions 45–61), and the final “X” indicates the source of the regulatory domain. LPhP57cs has a linker sequence comprising PurR 45–56 and LacI 57–61 [21]. LGhP comprises the LacI DNA binding domain, the GalR linker, and the PurR regulatory domain.

b: All proteins are from *E. coli*. LacI: Lactose repressor protein. RbsR: Ribsose repressor. FruR: Fructose repressor. GalR: Galactose repressor. GalS: Galactose isorepressor. PurR: Purine repressor. TreR: Trehalose repressor. AscG: Cryptic *asc* operon repressor.

c: Point mutations listed in this table were generated in earlier studies [17], [18]. For this study, LLhT/V52A and LLhA/Q55A were used to generate mutations at most other positions because, if mutations were carried out in the weak repressors LLhT and LLhA, subsequent functional changes might be undetectable (as occurred for many variants of LGhP). A second rationale for using chimeras with point mutations was to compare outcomes between polymorphic variants (for example LLhG and LLhG/E62K). In either case, the noted position was fixed while other linker positions are mutated. (For example, position 62 was not further mutated in LLhG/E62K.).

d: These values were determined in the absence of allosteric effector for all inducible repressors and LLhA, which has no known inducer. For the co-repressible chimeras based on PurR, values are shown for the presence of effector.

e Lacks the eleven C-terminal amino acids of the tetramerization domain [34].

Here, we have compared and contrasted results from parallel mutagenesis in multiple, synthetic LacI/GalR homologs. Positions chosen for mutation were nonconserved among the natural paralogs. In addition to synthetic paralogs, the mutated homologs included proteins with closer sequence relationships (synthetic orthologs and polymorphic variants). Regardless of the sequence relationship, mutational outcomes were highly context-dependent. In addition, results showed that outcomes from mutating nonconserved positions can differ significantly from mutating conserved positions.

## Materials and Methods

### Chimeric proteins and variants

Parent chimeric proteins used in this study were previously described [17]–[19], [21] with the exception of LGhP. Chimera nomenclature follows the convention of “LXhX”, where “L” indicates the DNA binding domain of LacI (amino acids 1–44), “Xh” represents the natural protein source of the linker region (amino acids 45–61a, which in LacI contains a hinge helix), and the final “X” indicates the natural source of the regulatory domain (Table 1). In addition to the parent LXhX chimeras, we used several constructs with points mutations; the rationales for these variants are stated in the footnotes of Table 1. LGhP was created by mutating the coding region for LLhP on the pHG165 vector, using the QuikChange protocol (Table S1 in Data S1). LGhP comprises LacI 1–45, GalR 44–59, and PurR 60–341.

For dimeric LacI (“LacI-11”) and all chimeras, linker variants were constructed *via* site-directed random mutagenesis as previously described [19]. Briefly, an ensemble of primers containing randomized nucleotides (“NNN”) at one of 12 linker position codons were used to create a mixture of mutated plasmids (Quikchange site-directed mutagenesis kit, Agilent, Santa Clara, CA). The variants of LLhS/R51S and LLhS/D62N were made with the LLhS primers using the LLhS/R51S/D62N template (depending on the position to be mutated, the primers reverted either position 51 or 62 to the original sequence). Mixed plasmids were transformed into 3.300 cells (Hfr(PO1), *lacI22, λ-, e14-, relA1, spoT1, thiE1*; *E. coli* Genetic Stock Center, New Haven, CT) and grown on LB-ampicillin (100ug/mL) plates in the presence of the β-galactosidase substrate 5-bromo-4-chloro-3-indolyl-β-D-galactopyranoside (Xgal) at 37°C. Colonies with a range of β-galactosidase activities were grown overnight in 48-well blocks containing 2.5 mL 2xYT media at 37°C. From these cultures, plasmid DNA was isolated using a 96-well kit (Qiagen, Valencia, CA), and the protein coding regions were sequenced (Northwoods DNA Inc., Solway, MN or ACGT Inc., Wheeling, IL).

For ∼500 variants, the entire coding region was sequenced to confirm that no additional mutations were present. Since second site mutations arose in fewer than 1% of variants, only the N-terminal region of the repressor coding region (which includes the linker) were sequenced for remaining variants. About 5% of samples showed either double sequence on the chromatogram at the site of mutagenesis or multiple phenotypes in subsequent plate assays (see below). Since these samples appeared to contain a mixture of repressor variants, we re-transformed the DNA, purified plasmid from new colonies, re-sequenced the coding regions, and verified single phenotypes. Mutagenesis results indicated a bias against methionine substitutions, possibly due to the fact that Met has only one codon. The random mutagenesis protocol is biased towards amino acids that have more codons.

### Protein expression and activity

For each variant, protein expression and activity were determined in crude cell extracts using a pull-down assay with immobilized *lacO^1^* operator [22] and visualized by SDS-PAGE [17], [18]. The high concentration of immobilized DNA in the pull-down assay allowed robust detection of even very weak repressors (DNA binding K_d_ ≥10^−7^ M). Results from pull-down assays were previously used to estimate a lower limit of ≥2500 repressor molecules per cell, which is in vast excess of the single *lac* operon per bacterial genome. Under these expression conditions, comparisons with thermodynamic assays suggested that, *in vivo*, most of the excess repressor protein is bound to nonspecific, genomic DNA, effectively buffering repression assays against fluctuations in repressor concentration [23].

In the current study, a few variants (<30) showed no binding to immobilized *lacO^1^* and were subsequently assayed with immobilized *lacO^sym^* operator [24], which generally has higher binding affinity for LacI variants. Approximately ten variants did not show activity in either assay. For these, we could not discriminate whether the protein was not expressed, was unstable, was unable to bind DNA with even nonspecific affinity, or bound genomic *E. coli* DNA so tightly that the immobilized DNA was unable to compete. These repressor variants remain in the composite data sheet but were excluded from other analyses.

### Beta-galactosidase activity assays

For each variant, transcription repression was assayed using the reporter enzyme β-galactosidase in 3.300 cells. These cells are Δ*lacI* but wild-type for *lacZYA* [17]. Briefly, the phenotypes of bacterial colonies expressing each variant were determined in the presence and absence of effector ligand on LB agar plates, MOPS minimal media agar plates, and in liquid culture MOPS minimal media (Teknova, Hollister CA). Effector ligand concentrations were as in [17]. Variants of LLhF and LLhS were induced by some component of rich media [17], so phenotypes were not determined with LB plates. For liquid culture assays, independent determinations were made for 2 to 4 separate bacterial colonies, each measured in quadruplicate or duplicate, respectively. In almost all cases, phenotypes from plate and liquid culture assays showed good agreement. In a few cases, two separate clones of a chimera variant (sometimes with a different codon) were assayed. Repression for these samples was usually within 2-fold of each other.

We previously defined three thresholds for repressor function: (i) We used DNA pull-down experiments [17] to confirm that all 1000+ variants were over-expressed at comparable levels and were capable of binding DNA. This assay was robust even for repressors with low DNA binding affinities (K_d_ >10^−7^ M) and positive results showed that mutagenesis did not prevent the repressors from folding into a structure capable of DNA binding. (ii) Quantitative *in vivo* repression assays were used to identify variants with measureable repression, relative to a “no repressor” control (“DEL” in Figures S1–S12 in Data S3). For LLhP and LLhG variants, changes in *in vivo* repression correlated strongly with altered DNA binding affinity (K_d_) for the *lacO^1^* operator across a wide-range of *in vivo* values [23], [25]. (iii) We previously determined a biological threshold by correlating *lac* repression strength and *E. coli* growth on lactose minimal media [17]: When LacI/GalR variants repressed β-galactosidase activity below 13 Miller units (tight and moderate repressors), essentially any change in repression was sufficient to alter bacterial growth; differences between weaker repressors (above 13 Miller units) had little effect on growth.

In data analyses, we used both the repression and growth thresholds as reference points. The growth threshold denotes biologically significant changes in repression of the *lac* operon, but the repression threshold delineates a larger range of changes that can occur within the repressor proteins. This distinction is important because the growth threshold is not only family-specific, but homolog- and environment-specific. For example, wild-type LacI and PurR should have different biological thresholds because the regulation of the *pur* regulon is central to purine metabolism [26], whereas regulation of the *lac* operon optimizes the use of alternative energy sources (reviewed in [27]) and guards against membrane leakage *via* excess lactose permease [28]. In a second example, the growth threshold might differ if other sugars were present or if the number of repressor copies was decreased (as is the case for wild-type *E. coli*). Nevertheless, for regulating the *lac* operon, crossing the growth threshold of 13 Miller units corresponds to a catastrophic functional outcome and this context is useful for some analyses.

Therefore, we defined a “change” as meeting the dual conditions of (i) >2-fold difference in repression and (ii) no overlap between the standard deviations. (The 2-fold limit was usually larger than the standard deviations of compared values.) We consider >2-fold change to be biologically significant, since colonies expressing these chimeras would have different growth curves if repression was tighter than 13 Miller units [17].

### Data analyses

For each repressor variant, sequence and functional data were compiled in a Microsoft Excel (Microsoft Corp., Redmond WA) spreadsheet (Tables S3–S19 in Data S2). When needed, a program written in Python was used to extract data from the Excel file. Data plots were generated with GraphPad Prism (GraphPad Software, Inc., LaJolla, CA). Physico-chemical similarities (Table S20 in Data S2) were mapped onto bar graphs using an in-house C# program. New, unique sequences for the expanded LacI subfamily were generated by a BLAST [29] search of RefSeq (cut-off date May 7, 2011) [30] as previously described [31]. “Sequence entropy” quantifies conservation (or nonconservation) by calculating the frequency with which each amino acid appears in a particular column of an MSA and was calculated with the program BioEdit [32]; these values are summarized in Table 2. Solvent accessibility of the side chains in LacI and PurR structures was calculated with “Contacts of Structural Units” [33].

**Table 2:** Sequence entropies^a^ of LacI/GalR linker positions, calculated from various MSAs.

Linker position	All Seqs	YPAL Seqs	LacI subfamily
L45^b,c^	1.20	1.01	0.00
46	1.56	1.62	0.86
Y47	0.24	0.07	0.00
48	2.25	1.99	0.93
P49	0.70	0.00	0.00
50	1.20	0.60	0.00
51	2.15	1.92	0.07
52	2.24	1.80	0.93
A53	0.91	0.00	0.00
54	1.37	0.82	0.00
55	2.21	1.67	0.37
L56	0.96	0.00	0.00
A57	1.98	1.70	0.36
58	2.37	2.26	0.20
59	2.14	1.66	0.20
60	2.28	2.16	1.28
61	1.68	1.03	1.07
62	2.30	2.18	1.47

a Sequence entropy  = −Σ_i = 1_^21^ (f_i_*ln (f_i_)), where “f_i_” is the frequency of occurrence for each amino acid or gap at the given linker position. A sequence entropy value of zero (0) corresponds to perfect conservation; equal frequency of all 21 possibilities corresponds to 3.04.

b: Positions 47, 49, 53, 56, and 57 were not mutated in the current study.

c: The LacI, PurR, GalR, and all chimeras of this study have leucine at position 45.

## Results

Using the family of 14 synthetic LacI/GalR proteins [17] and dimeric LacI [34] (Table 1), we compared and contrasted the functional outcomes for >1000 variants at nonconserved linker positions 46, 48, 50, 51, 52, 54, 55, 58, 59, 60, 61, and 62. These positions have various levels of conservation among the LacI/GalR paralogs, whereas four other linker positions (47, 49, 53, and 56) are highly conserved (Table 2; delineation of nonconserved and conserved positions is further discussed below). Mutagenesis was accomplished *via* a site-directed random protocol [19], which usually yielded 8–12 amino acid substitutions at each position. Protein expression and ability to bind DNA were verified for all variants. Next, *in vivo* repression of the *lac* operon was assayed in the presence and absence of allosteric effectors [17]. (Allosteric effectors bind to the regulatory domains of LacI/GalR homologs and thereby alter DNA binding affinity).

Although a phenomenal dataset exists for tetrameric LacI, [35], [36] our new LacI experiments ensured that protein expression levels were comparable to those of the chimeras and that repression was measured quantitatively. Data for tetrameric LacI were reported qualitatively, with “wild-type” values that varied up to 200-fold [35], [36]. We used a version of LacI that lacks the C-terminal tetramerization domain [34], because none of the other homologs contain a tetramerization domain.

Representative results are shown in Figure 1 B–D; all other results are in Figures S1–S12 in Data S3. Statistical and biological considerations of repression “change” are discussed in Methods; usually, the assay reliably detected repression values that differed >2-fold (a few exceptions had large error bars).

Both enhancing and inhibiting substitutions were found for all repressors. However, the fraction of enhancing mutations was related to the repression of the starting protein. When the starting repressor was strong (*i.e*. LacI-11, LLhR), very few amino acid substitutions further increased repression. These strong repressors might be near the detection limit for the β-galactosidase assay, or repression itself might have a limiting value. When the starting repressor was weak (*i.e.* LGhP), again only a small percent of substitutions enhanced repression, probably because multiple amino acid changes are required. However, when the starting repressor was intermediate (*i.e.* LLhF), a larger percent of substitutions were enhancing.

More than 100 variants showed repression comparable to or tighter than LacI-11 (0.12±0.06 Miller units); these included variants of all parent proteins except those with PurR regulatory domains. Around 9% of single substitutions enhanced repression ≥5-fold relative to the starting protein, with a maximal enhancement of ∼1000-fold. All positions but 50 had at least one substitution that enhanced repression >5-fold (Table 3), but a larger fraction of enhancing substitutions was observed at positions 62, 51, and 55 (19%, 11%, and 9%, respectively; Table 3).

**Table 3:** Frequency of substitutions that enhance repression.

	46	48	50	51	52	54	55	58	59	60	61	62
Total^a^ variants	114	102	96	100	113	117	113	95	101	107	92	126
Enhance >10-fold	4	1	0	10	7	1	9	5	1	3	2	21
% Subst'ns enhanced	4^b^	1	0	11	7	1	9	6	1	3	3	19
Parent proteins mutated	13	14	14	13	14	13	14	13	14	14	14	13
Parent proteins enhanced	2^c^	1	0	5	7	1	5	3	1	3	1	6

a: Each parent protein was counted as one of the amino acids in all 12 positions.

b: % substitutions enhancing  =  (enhanced >10-fold)/(Total variants – Parent proteins mutated).

c: All enhancing substitutions at positions 46 and 48 occurred in “LXhX” chimeras, which had domain fusion between the DNA binding domain and linker.

Finally, since the linker positions bridge the DNA binding and regulatory domains, we anticipated a number of linker variants would alter allosteric response to effector. However, most amino acid substitutions altered repression only in the absence of effector. In the presence of effector, most of the variants matched their parent repressor, in either (i) always inducing to the level of no repressor (“DEL” control), or (ii) maintaining the same magnitude of response to effector. Of all 1000+ variants, only ∼30 substitutions showed a different outcome +/− effector and thus altered allosteric response.

### Nonconserved positions serve as “rheostat” locations for modifying protein function

For each mutated position in each homolog, we generated multiple amino acid substitutions (Figure 1 B–D). When the substitutions were rank-ordered by their functional outcomes, results generally showed one of three patterns: A given position could function as a “toggle”, a “rheostat” or a “neutral” location for modifying protein function (Table 4, footnotes). “Toggles” were defined by biphasic outcomes; some amino acids conveyed repression similar to the parent protein; other amino acids abolished function. Toggle behavior has been commonly expected at highly conserved positions in other proteins. “Rheostats” were defined by *progressive* repression changes that spanned at least two orders of magnitude. “Neutral” positions were defined by most amino acid substitutions having ≤2-fold change on repression (the limit of the assay) relative to the starting protein. Repression changes >2-fold can alter bacterial growth if the parent protein represses tightly (to ≤13 Miller units) [17].

**Table 4:** Rheostat, toggle-like, and neutral behaviors of nonconserved linker positions^a^.

	46	48	50	51	52	54	55	58	59	60	61	62
LacI-11	R	R	R	3^b^	R	R	r	R	R	n	R	N^c^
LLhR	N+r^d^	N+R	#	3	R	R	R	3	R	R	R	R+^e^
LLhF	r	R	r	R	R	R	R	R	R	R	r+T	R
LLhG	N	#	T	R	n/r^f^	#	r	R	#	r	n/r	R
LLhG/E62K	N+r	R	R	r	R	R	R	R	r	n/r	#	–
LGhG	R	r	#	R	r	#	R	R	r	n+T	R	R
LLhP	r	R	R+T	R	R	R	3	T	R	r	T	3
LPhP57cs	n/r	r	T	R	n/r	r/T	R	T	r	r	x	T+R^g^
LGhP	r	R	#	x	#	–	x	r	R	r	#	x
LLhS	R	#	#	r	#	#	#	#	R	r	#	R
LLhS/R51S	–	–	–	–	–	–	–	–	R	r	r	R
LLhS/D62N	–	#	#	#	#	T	#	–	–	–	–	–
LLhS: R51S/D62N	r	r	R	–	R	R	r+T	R	R	#	#	–
LLhT	–	–	–	–	3	–	–	–	–	–	–	r
LLhT/V52A	r	R	R+T	T	–	R	R	R	R	r	R	R
LLhA/Q55A	r+T	r	#	R	R	R	R	R	R	N	R	N

a: “R, rheostatic (progressive) changes that span >2 orders of magnitude; “r”, rheostat character but <2 orders of magnitude. “T”, toggle-like. “N”, neutral (within 2-fold change); “n”, between 2 and ∼5-fold change. “#”, insufficient number of substitutions to determine behavior. “–”, not mutated. “x”, weak or no measureable repression for any substitution.

b:. Substitutions generated 3 states instead of a continuum. Either 2- or 3-state toggles might reveal rheostat behavior if additional amino acid substitutions were characterized. However, in addition to reported variants, no intermediates were identified for the 2-state toggles during colony selection, which involved visual inspection of *lac* repression for several hundred bacterial colonies expressing randomly mutated chimeras.

c: In designating a neutral position, we invoked the caveat “most amino acids” because, for example, proline and glycine substitutions can have large backbone effects. Nevertheless, *all* reported variants bound DNA in the pull-down assay, which indicated that the protein structure was not grossly distorted.

d: Seven (7) of 11 amino acids are neutral; the remaining 4 have rheostat character.

e: A subset of positions had rheostat behavior, and another subset abolished detectable repression.

f: Substitution results were between neutral and rheostat behavior (∼5–9 fold change).

g: Four substitutions convey equally enhanced repression; another 6 have rheostat character.

In most of the repressors, the mutated linker positions generally behaved as functional rheostats (Table 4; Figures S1–S12 in Data S3). In contrast, the Miller lab showed that 11–13 substitutions at each of positions Tyr47, Pro49, Ala53, and Leu56 abolished measurable repression in LacI (*i.e.* function as toggles) [35], and in PurR, the position analogous to Leu56 only tolerated methionine out of seven substitutions [37]. These four positions comprise a “YPAL” motif that is conserved in ∼60% of LacI/GalR linker sequences; the presence or absence of this motif correlates with different classes of DNA ligands [31], [38].

Since the rheostat and toggle behaviors appeared to correlate with (non)conservation, we compared prior bioinformatics predictions [31] to the current experimental data. The previous work was carried out using the whole LacI/GalR family, and various computational analyses did not discriminate the two experimental classes. However, a two-tiered analysis of the LacI/GalR sequences separated the rheostat and toggle positions: First, we noted that most linker positions showed some degree of nonconservation in the whole sequence set. Nevertheless, the “PAL” of the YPAL motif were among the strongest co-evolving positions in the complete LacI/GalR family. (Y is conserved and thus not detected) [31]. Second, we noted that if sequences were restricted to a subfamily (for example, the LacI orthologs), both rheostats and toggles appeared to be conserved. Instead, an intermediate sequence set that included only YPAL-sequences separated toggles and rheostats based solely on their sequence entropies: Rheostat positions had sequence entropies ≥0.6 (Table 2), whereas toggle positions (47, 49, 53, and 56) had values <0.1.

Although rheostat behavior dominated the nonconserved positions, rheostat behavior was not always consistent among homologs. Nonconserved positions occasionally showed toggle or neutral behaviors (for example, positions 58 and 62 in Table 4). In some cases, hybrid behaviors were observed. For example, at LLhR position 62, seven amino acids produced a rheostat outcome, whereas three amino acids toggled repression “off”. Even closely-related repressors – such as the synthetic iso-repressors LLhG and LLhS – showed different behaviors at some positions. The underlying reasons for behavior switches have yet to be elucidated. Until that is accomplished, the homolog-specific behavior clearly illustrates why mutagenesis of a single homolog is insufficient for benchmarking MSA analyses of a protein family.

### Mutational epistasis occurs frequently

**Figure 2:**
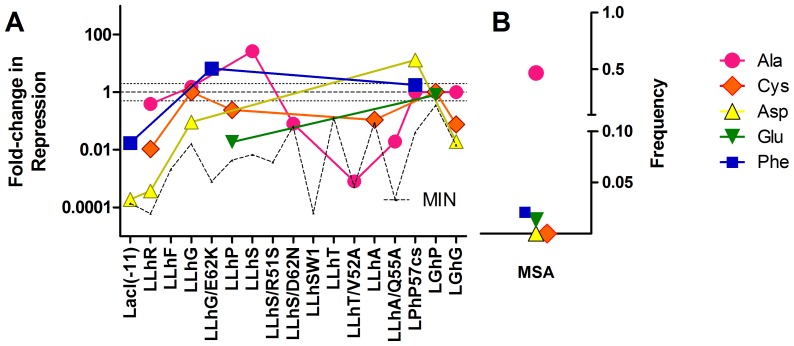
Substitution outcomes do not correlate with amino acid frequency. (A) Substitution outcomes for position 51 among the LacI/GalR chimeras; 5 amino acid substitutions are shown. Each starting protein had different repression activity, which was used to normalize its variants. No change corresponds to a value of 1 (dashed black line). The straight dotted lines indicate 2-fold change from the starting protein; this range is usually larger than the error bars of a repression measurement. Substitutions that enhance repression have increased fold-change (>2). Substitutions that diminish repression have decreased fold-change (<2). The jagged dotted line shows the no repressor “DEL” control relative to the starting protein and represents the lowest possible value. Colored connecting lines are to aid visual inspection of the data. (B) Amino acid frequency in the naturally occurring proteins at position 51, as calculated from the MSA of LacI/GalR proteins with a “YPAL” motif. Even though Ala occurs with high frequency, this substitution can be catastrophic. Further, even though Asp is absent from the natural sequences, this substitution can enhance repression in at least one chimera.

At the beginning of this study, one question of particular interest was whether an individual amino acid had similar outcomes among various homologs. To that end, we compared the functional rank order of amino acids among the repressors. Representative results are shown in Figure 2A; all other results are in Figures S13–S24 in Data S4. Several amino acid substitutions had widely different outcomes among homologs (“D” in Table 5). Outcomes at positions 51, 55, and 62 were frequently disparate, perhaps because these positions can interact with the alternative regulatory domains [20], [39], [40]. From the amino acid perspective, Leu, Arg, and Tyr were prone to different outcomes among homologs. Even Ala did not follow simple substitution rules: At some positions (*e.g.* 46 and 60), Ala substitutions were neutral, and the position would be missed as “important” in an alanine scan; at other positions (*e.g.* position 51), Ala substitutions had varied outcomes. Since the linkers are otherwise identical among the LLhX chimeras, the different outcomes indicated significant influence of the alternative regulatory domains on the rheostat positions.

**Table 5:** MSA frequency *versus* substitution outcome and results from parallel mutagenesis^a^.

	46	48	50	51	52	54	55	58	59	60	61	62^b^
Ala		P1		P2D	D	P1D	P1D	P1	P1		P1	D
Cys	–	A	A	A	A		D	P1			P1	–
Asp		–		D			P2	–	–	A		P1D
Glu	P1		–	P1			AD	P2	–			
Phe	A	A		P1D	P1D	–	D			–	P2	A
Gly		P2		P1D	P2		P2	XD	P1D	P2D		A
His		P1		–	P1D			–	P1	P1		
Ile		X		P2D	D	P2	P2D	P2		A		AD
Lys	A	P2				P1		P1D	X		L	
Leu	A	AD		P1D	P1D		P1D	P2	P1	A	AD	D
Met	–	–	–	AD	P1	–	AD	–		–	–	A
Asn	X		X	P1		–	P1	P1		–	A	P1D
Pro				AD	A					AD	A	P2D
Gln					P1	X	X		P1	X	L	
Arg	P1D	P1D		X		P2	P2D	P1D	D		L	
Ser		P2	P2	P2D	P1	P2	P1	P1D	P1		X	A
Thr	A	P1		P1	P1	P2	AD	P1D	P1		P1D	
Val	A				X		AD	P2		P2		AD
Trp	D	A		AD						–		A
Tyr	A			P2D	D	–	AD	–		–	AD	

a: “X”  =  the starting amino acid for LXhX chimeras. “D”  =  substitution caused widely different outcomes among several chimeras. “A”  =  Amino acid absent from the YPAL-MSA but allowed repression near or better than parent protein in 2 or more chimeras. “P1”  =  Amino acid present in the YPAL-MSA but diminished repression below the biologically determined threshold of 13 Miller units for at least one chimera. “P2”  =  Amino acid present in MSA but mutation diminished repression to the “no repressor” limit (“MIN” in Figure 2) for at least one chimera. “L”  =  Amino acid was absent in MSA; allows strong repression in LacI though not other chimeras (LacI data are commonly used as a single representative of the family.) “–”  =  an insufficient number of substitutions were isolated to determine general outcome.

b: In the un-mutated chimeras, position 62 differs for each regulatory domain.

Context-dependent outcomes were evident for even closely-related proteins: In addition to synthetic paralogs (*i.e*. LLhP and LLhG from paralogs PurR and GalR), this study included synthetic orthologs (LLhG and LLhS from the *E. coli* iso-repressors GalR and GalS [41]) and polymorphic variants (*i.e*. LLhG and LLhG/E62K). Both orthologs and polymorphs could show different outcomes from the same amino acid substitutions. That is, a change at a single nonconserved position could have wide-ranging consequences. These results were not predicted by current MSA analyses, since the nonconserved linker positions do *not* co-evolve with each other [31]. For the polymorphic variants, the disparate outcomes clearly demonstrate molecular-level epistasis.

Indeed, results illustrate a second difference between nonconserved and conserved positions. For conserved toggles, compensatory mutations are often described like two switches that control the same light; switching one “off” is compensated by switching the other “on”. In contrast, when two LacI/GalR rheostat positions influenced each other, changing an amino acid at one rheostat position *re-ordered the amino acid preference* at the second position. For example, at LLhS position 51 (Figure S4 in Data S3), Ala ≈ Gly < Ser ≈ Arg; whereas in LLhS/D62N, Ser ≈ Gly < Arg < Ala.

### “Conservative” amino acid changes had disparate outcomes

For conserved positions, amino acids with similar physico-chemical properties (*i.e.* Val and Ile, or Asp and Asn) are often considered to be interchangeable. This idea arose from the observation that, in naturally-occurring proteins, substitutions between similar amino acids occur more frequently than others [42]–[44]. However, in the rank-order plots of the current studies, the functions of “similar” substitutions were frequently interspersed by other amino acids (for two examples – Ile *vs.* Leu and Thr *vs*. Ser – see position 52 in LLhF; Figure S5 in Data S3). Indeed, substitution outcomes at nonconserved linker positions showed little family-wide correlation with aromaticity, hydrophobicity [45], accessible surface area of the free amino acid [46], side chain branching, or position-specific helical propensity [47] (Table S20 in Data S2; color coding of Figures 1B–D and Figures S25–S87 in Data S5, Data S6, and Data S7). Charged amino acids were often disruptive; however, this was not too surprising given the close proximity to charged DNA ligand. Patterns were occasionally observed for individual chimeras but never extended to *all* of the parent proteins. This is probably *not* a peculiarity of the LacI/GalR linkers, since disparate outcomes for “conservative” substitutions were also observed in human growth hormone [48].

### The LacI/GalR MSA does not predict amino acid outcomes

Several MSA analyses are based on the rationale that amino acids of the natural proteins reflect “allowed” substitutions, whereas amino acids absent from the natural proteins are disallowed. Therefore, we compared substitution outcomes to amino acid frequency in the YPAL subset [31] of the natural LacI/GalR proteins (Figure 2B and Figures S13–S24 in Data S4). Of the mutated positions, only position 50 showed reasonable correlation with MSA frequency, with the naturally occurring Asn and Ser residues usually having tightest repression. Nevertheless, several other amino acids at position 50 allowed measurable repression. For all other positions, repression and MSA inclusion were poorly correlated.

For example, cysteine was absent from the YPAL-MSA at most linker positions but was well-tolerated (repressed similar to or better than the parent protein) at four positions (Table 5, “A”). Cysteine may be evolutionarily unfavorable because disulfide bonds could cross-link the repressor dimer. Evolutionary rationales were more difficult to conceive for the other tolerated amino acids that were absent from various positions (Table 5, “A”). Eight of 12 linker positions tolerated amino acids missing from the YPAL-MSA, which indicates this behavior is likely to be widespread. The YPAL-MSA might be incomplete (too few species sequenced) or information might be lost during the common practice of MSA sub-sampling. Indeed, we observed at least two instances in which the subsampled YPAL MSA *lacked* amino acids that were present in the LacI subfamily. This might be a general problem with subsampling, as the LacI/GalR MSA [31] is derived from a large number of bacterial genomes and thus is larger than MSAs used for many other protein families. However, 70 amino acids that were *present* in the linker positions of the YPAL-MSA reduced repression to the level indistinguishable from “no repression” in at least one chimera (“P1”and “P2” in Table 5) [17]; twenty-five of these abolished all measurable repression (“P2” in Table 5). Thus, we conclude that the frequency with which an amino acid occurs in the natural sequences does not predict mutational outcomes for nonconserved, rheostat positions.

Frequency comparisons were repeated using the LacI-11 dataset and the LacI-subfamily MSA. This subfamily has pairwise sequence identity of 36–99%, which more closely resembles datasets used by two popular MSA analyses: SIFT [7] and Poly-phen-2 [8]. (By comparison, the sequence identities of the YPAL- and full LacI/GalR family drop as low as 15%.) Surprisingly, the LacI MSA exacerbated the disconnect between our experiments and evolutionary information: Many linker positions *not* conserved in the YPAL-MSA *are* highly conserved in the LacI subfamily (Table 2 and Table S2 in Data S1), yet our experimental dataset contained many well-tolerated amino acid substitutions. These substitutions would perhaps be erroneously predicted to be catastrophic from the restricted sequence set.

### Rheostat and toggle positions do not show obvious structural differences

Finally, we assessed whether rheostat and toggle positions could be separated by structural considerations. Inspection of the DNA-bound LacI [39] and PurR [40] crystal structures did not uncover any compelling differences. For example, both toggles (A53 and L56) and rheostats (51 and 54) interact with DNA ligand [20]. Several rheostat positions (especially 48 and 52) are as buried as the toggle positions. Both toggles and rheostats are subject to the linker conformational change observed in LacI [49]. This change was absent in LLhP [25]. Since LLhP has a slightly higher occurrence of toggle positions (Table 4), perhaps a relationship exists between protein dynamics and rheostat behavior.

## Discussion

A recent survey of >10,000 laboratory-induced protein mutations found a strong bias towards amino acid positions that are conserved during evolution [2]. Since nonconserved positions can also play important functional roles, we systematically monitored the outcome of mutagenesis at nonconserved positions in synthetic LacI/GalR homologs. Our key finding is that mutational outcomes showed a striking context-dependence, which probably explains why the products of computational protein design can often be enhanced by directed evolution. A second key finding of our study was that nonconserved positions served as rheostat locations for modifying protein activity. This contrasts with the toggle behavior of conserved positions. Further, mutational outcomes at rheostat positions differ significantly from those of toggle positions.

We have considered the possibility that rheostat behavior is just a peculiarity of the LacI/GalR linker regions. However, rheostat-like behavior is present in the data for some positions of the PDZ domain [50], E3 ubiquitin ligase [51], and two Bcl-2 homologs [52], which are among the very few proteins that have been (i) subjected to saturating mutagenesis and (ii) assayed in a way that allows detection of rheostat behavior. (Results from saturating mutagenesis of the WW domain [53] could also be re-plotted by position to look for rheostat positions.) In all of these proteins, it will be interesting to determine whether the rheostats occur at nonconserved positions.

It bears repeating here that our current definitions of “conserved” and “nonconserved” are based upon a specific group of LacI/GalR paralogs. Identification of the relevant sequence set required a “Goldilocks” approach: The whole LacI/GalR family was too large, and all linker positions appeared to be nonconserved. The ortholog subfamilies (with >40% sequence identity) were too small, and most linker positions appeared to be conserved. However, using co-evolution to divide the sequences (based on the presence of the YPAL linker motif) identified the “just right” sequences. Using these data, the toggle and rheostat positions largely separated as conserved and nonconserved positions.

This analytical approach is unlikely to be duplicated by currently available automatic methods, but our study provides guidelines for replicating this analysis in other protein families. First, deep phylogeny is required for the protein family; that is, the sequence identity cutoff for the family should *not* be limited to >40%. Second, we predict that toggles can be identified in the full sequence set as co-evolving and conserved positions. (The exception to this strategy was position 50, which functioned as a rheostat but also showed strong co-evolution with the YPAL motif. The evolutionary pressure apparently exerted on position 50 remains a mystery; the amino acids at this position are *not* encoded by rare codons.) Third, in our hands using the data from the whole LacI/GalR family, the algorithms TEA-O [11] and Evolutionary Trace Analysis (“ETA”, [13]) identified the greatest number of important linker positions [31], comprising both rheostats and toggles. Thus, for other protein families, analyses with conservation, co-evolution, and TEA-O/ETA could be combined to discriminate toggles and rheostats.

Finally, our prior study predicted that >50% of positions in the LacI/GalR family are important for function [31]. Since most of these are neither co-evolving nor conserved, rheostat positions may be more common than either toggle or neutral positions in the LacI/GalR proteins. We expect that a similarly high density of rheostat positions will occur on other protein scaffolds that have evolved a variety of functional modifications, whereas highly conserved proteins might contain a higher percent of toggle positions. Understanding the nature of a protein position can help researchers predict either rheostat or toggle outcomes upon mutagenesis. The different mutagenesis outcomes for toggle and rheostat positions compel future studies of nonconserved positions as crucial for advancing protein engineering and predicting the medical impact of polymorphisms in human exomes. Additional data described in this work can be found in the online Supporting Information.

## Supporting Information

Data S1 **Supporting tables.** Table S1. Primers used in the construction of LGhP. Table S2. Amino acid frequency in the linker positions of the LacI subfamily.
(PDF)

Data S2 **Supporting tables.** Table S3. Values from repression assay for LacI-11 variants. Table S4. Values from repression assay for LLhR variants. Table S5. Values from repression assay for LLhF variants. Table S6. Values from repression assay for LLhG variants. Table S7. Values from repression assay for LLhG/E62K variants. Table S8. Values from repression assay for LGhG variants. Table S9. Values from repression assay for LLhP variants. Table S10. Values from repression assay for LPhP and LPhP57cs variants. Table S11. Values from repression assay for LGhP variants. Table S12. Values from repression assay for LLhS variants. Table S13. Values from repression assay for LLhS/R51S variants. Table S14. Values from repression assay for LLhS/D62N variants. Table S15. Values from repression assay for LLhS/R51S/D62N variants. Table S16. Values from repression assay for LLhT variants. Table S17. Values from repression assay for LLhT/V52A variants. Table S18. Values from repression assay for LLhA variants. Table S19. Values from repression assay for LLhA/Q55A variants. Table S20. Physico-chemical properties of amino acids.
(XLSX)

Data S3 **Supporting figures.** Figures S1–S12. Beta-galactosidase reporter gene assays: positions 46–62. These plots show results from β-galactosidase (lacZ) reporter gene assays for >1000 variants of the LacI/GalR chimeras and LacI-11. Lower values correspond to tighter repression of the *lac* operon. The first bar in each graph is labeled DEL (black) and shows β-galactosidase activity in the absence of repressor protein. Below 13 Miller units (solid black line), any change in repression altered bacterial growth [17]. The red dashed lines indicate the activities of un-mutated, starting proteins. Note that some red lines obscure the black lines in some panels. Error bars are the standard deviation of 2–4 independent bacterial colonies, each in quadruplicate or duplicate. All variants showed expressed and active protein *in vivo*, as assessed by the DNA pull-down assay. In Figures S1–12, data are organized to show all variants at a given position (*e.g.* position 46) in all mutated proteins on one page. Assays were carried out in the absence and presence of allosteric effectors [17]. For all inducible repressors, the front colored series shows repression in the absence of effector and the back gray series show repression in the presence effector. For the co-repressible chimeras based on PurR (LLhP, LPhP57cs, LGhP), the front series shows repression in the presence of effector and the back series shows repression in the absence. LLhA variants have no known allosteric effectors. Exceptions to the general description: (1) Values for the LLhA/Q55A variants R51L, R51M, and V52L were only determined from one days assay (2 colonies each in duplicate). (2) The LGhG variants H48I and H48N are not shown on the following plots but had white phenotypes (tight repression) in plate assays. In liquid culture assays, these variants appeared to be toxic to *E. coli*. Plate assays results were taken into consideration when assigning rheostat behavior to position 48 in LGhG (Table 4 in the main document).
(PDF)

Data S4 **Supporting figures.** Figures S13–S24. Parallel amino acid substitutions: positions 46–62. These plots compare the outcomes of parallel amino acid substitutions among the LacI/GalR homologs. Results are organized to show all variants obtained at a position on one page; five amino acids are shown per panel, as indicated in the legends at the right. In the left-hand panels, data for each variant were normalized relative to the starting protein, which had different repression values (Table 1 in the main document). If an amino acid substitution caused no change, this corresponds to a value of 1 on the plots (dashed black line). As discussed in Methods, data within 2-fold are considered equivalent to each other, which is indicated by the straight dotted lines on the plots; this range is usually larger than the error bars of a repression measurement. Substitutions that enhanced repression have increased fold-change (>2). Substitutions that diminished repression have decreased fold-change (<2). The jagged dotted line labeled MIN shows the no repressor DEL control relative to the starting protein and represents the lowest possible value for each homolog. The colored connecting lines are to aid visual inspection of the data. The right-hand panels show amino acid frequency in the naturally occurring proteins at the relevant positions, as calculated from the YPAL-MSA. The following definitions were used to assign the results in Table 5: “A”  =  amino acid absent from the YPAL-MSA but substitution allowed repression near the parent value or better in two or more chimeras; “P1”  =  amino acid present in the YPAL-MSA but substitution diminished repression below the biologically determined threshold of 13 Miller units (not shown) for at least one chimera; “P2”  =  amino acid present in the YPAL-MSA but substitution diminished repression to MIN; these designations were only used for parent chimeras with repression better than 13 Miller units; “D”  =  substitution caused widely different outcomes among several chimeras; “L”  =  absent in the YPAL- MSA but allowed strong repression in LacI-11. LacI data are commonly used as a single representative of the family for benchmarking MSA analyses.
(PDF)

Data S5 **Supporting figures.** Figures S25–S54. Physico-chemical trends: positions 46–54. Repression assay data for each position were color-coded according to various physico-chemical scales (Table S20 in Data S2). For example, Figures S25–S28 show results for position 46 color-coded by accessible surface area of the free side chain [46], side chain branching, charge/polarity/aromaticity, and hydrophobicity [45]. The relevant parameter can be determined from the legend in the lower right hand corner of each graph. Positions 50–58 have potential to participate in an alpha helix, and repression assay results were also compared to both average and position-specific helical propensities [47]. For simplicity, only one helix color scale is used as a legend, with magenta corresponding to high propensity and green corresponding to low propensity. Since wild-type LacI and PurR have different length helices [20], we compared multiple helical scales to results for each position. However, no scale showed good correlation with the functional assay among all chimeras.
(PDF)

Data S6 **Supporting figures.** Figures S55–S72. Physico-chemical trends: positions 55–59. Repression assay data for each position were color-coded according to various physico-chemical scales (Table S20 in Data S2). For example, Figures S55–S57 show results for position 55 color-coded by accessible surface area of the free side chain [46], side chain branching, and charge/polarity/aromaticity. The relevant parameter can be determined from the legend in the lower right hand corner of each graph. Positions 50–58 have potential to participate in an alpha helix, and repression assay results were also compared to both average and position-specific helical propensities [47]. For simplicity, only one helix color scale is used as a legend, with magenta corresponding to high propensity and green corresponding to low propensity. Since wild-type LacI and PurR have different length helices [20], we compared multiple helical scales to results for each position. However, no scale showed good correlation with the functional assay among all chimeras.
(PDF)

Data S7 **Supporting figures.** Figures S73–S87. Physico-chemical trends: positions 60–62. Repression assay data for each position were color-coded according to various physico-chemical scales (Table S20 in Data S2). For example, Figures S73–S75 show results for position 60 color-coded by accessible surface area of the free side chain [46], side chain branching, and charge/polarity/aromaticity. The relevant parameter can be determined from the legend in the lower right hand corner of each graph. (Note that the legend is placed in a middle location for position 62, in order to place results for LLhT and LLhT/V52A in close proximity).
(PDF)
