# Supplementary material for: Rheostats and Toggle Switches for Modulating Protein Function
Source: PLoS One. 2013 Dec 30;8(12):e83502. doi: 10.1371/journal.pone.0083502 (PMC3875437; doi:10.1371/journal.pone.0083502)
Supplement: Data S1 — Supporting tables. Table S1. Primers used in the construction of LGhP. Table S2. Amino acid frequency in the linker positions of the LacI subfamily. (PDF) [file pone.0083502.s001.pdf]

Supplemental Material for:
Rheostats and toggle switches for modulating protein function
File 1: Supplementary Tables

Meinhardt, Manley, Parente and Swint-Kruse

List of Tables

S1 Primers used in the construction of LGhP 2
S2 Amino acid frequency in the linker positions of the LacI subfamily 3

Table S1: Primers used in the construction of LGhP

Name	Sequence
LhG-NL	GCGGCGATGGCGGAGCTCAGTTACCATCCCAAC
rev LhG-NL	GTTGGGATGGTAACTGAGCTCCGCCATCGCCGC
LhG-Hhalf	CCCAACCCCGAGGCACGAGAACTGGCG
rev LhG-Hhalf	CGCCAGTTCTCGTGCCTCGGGGTTGGG
LhG-Hfull	CCCAACGCCAACGCACGAGCACTGGCG
rev LhG-Hfull	CGCCAGTGCTCGTGCGTTGGCGTTGGG
LGhPChalffor	CAACTGGCGCGCCAAAAGACGAAATCTATCGG
LGhPChalfrev	CCGATAGATTTCGTCTTTTGGCGCGCCAGTTG
GhPCLFor	CAACTGGCGCAACAAACCACGAAATCTATCGG
GhPCLRev	CCGATAGATTTCGTGGTTTGTTGCGCCAGTTG
Gh-Fix1	GAACGCCAACGCCCGTGCGCTGGCGC
rev Gh-Fix1	GCGCCAGCGCACGGGCGTTGGCGTTC
GhP-Fix2	CTGGCGCAGCAGACCACTAAATCTATCG
rev GhP-Fix2	CGATAGATTTAGTGGTCTGCTGCGCCAG

Table S2: Amino acid frequency (%) in the linker positions of the LacI subfamily.

	45	46	47	48	49	50	51	52	53	54	55	56	57	58	59	60	61	62
A								1	100				88			9	13	
C																		
D																		1
E																		
F																		
G								3						95				
H		17					1									12		
I				55				1			1						6	
K															95			
L	100							12			9	100					1	53
M																		9
N		70				100												
P					100												1	16
Q										100	90					61		1
R		12					99							5	5	5		5
S		1						10								5	69	1
T				6												8	8	12
V				38				73					12				1	
W																		
Y			100															1
GAP				1														
