# Supplementary material for: Rheostats and Toggle Switches for Modulating Protein Function
Source: PLoS One. 2013 Dec 30;8(12):e83502. doi: 10.1371/journal.pone.0083502 (PMC3875437; doi:10.1371/journal.pone.0083502)
Supplement: Data S3 — Supporting figures. Figures S1–S12. Beta-galactosidase reporter gene assays: positions 46–62. These plots show results from β-galactosidase (lacZ) reporter gene assays for >1000 variants of the LacI/GalR chimeras and LacI-11. Lower values correspond to tighter repression of the lac operon. The first bar in each graph is labeled DEL (black) and shows β-galactosidase activity in the absence of repressor protein. Below 13 Miller units (solid black line), any change in repression altered bacterial growth [17]. The red dashed lines indicate the activities of un-mutated, starting proteins. Note that some red lines obscure the black lines in some panels. Error bars are the standard deviation of 2–4 independent bacterial colonies, each in quadruplicate or duplicate. All variants showed expressed and active protein in vivo, as assessed by the DNA pull-down assay. In Figures S1–12, data are organized to show all variants at a given position (e.g. position 46) in all mutated proteins on one page. Assays were carried out in the absence and presence of allosteric effectors [17]. For all inducible repressors, the front colored series shows repression in the absence of effector and the back gray series show repression in the presence effector. For the co-repressible chimeras based on PurR (LLhP, LPhP57cs, LGhP), the front series shows repression in the presence of effector and the back series shows repression in the absence. LLhA variants have no known allosteric effectors. Exceptions to the general description: (1) Values for the LLhA/Q55A variants R51L, R51M, and V52L were only determined from one days assay (2 colonies each in duplicate). (2) The LGhG variants H48I and H48N are not shown on the following plots but had white phenotypes (tight repression) in plate assays. In liquid culture assays, these variants appeared to be toxic to E. coli. Plate assays results were taken into consideration when assigning rheostat behavior to position 48 in LGhG (Table 4 in the main document) [file pone.0083502.s003.pdf]

Supplemental Material for:
Rheostats and toggle switches for modulating protein function
File 3: Beta-galactosidase reporter gene assays

Meinhardt, Manley, Parente and Swint-Kruse

List of Figures

S1 Beta-galactosidase reporter gene assay: Position 46 3
S2 Beta-galactosidase reporter gene assay: Position 48 4
S3 Beta-galactosidase reporter gene assay: Position 50 5
S4 Beta-galactosidase reporter gene assay: Position 51 6
S5 Beta-galactosidase reporter gene assay: Position 52 7
S6 Beta-galactosidase reporter gene assay: Position 54 8
S7 Beta-galactosidase reporter gene assay: Position 55 9
S8 Beta-galactosidase reporter gene assay: Position 58 10
S9 Beta-galactosidase reporter gene assay: Position 59 11
S10 Beta-galactosidase reporter gene assay: Position 60 12
S11 Beta-galactosidase reporter gene assay: Position 61 13
S12 Beta-galactosidase reporter gene assay: Position 62 14

Figures S1-S12. These plots show results from β-galactosidase (lacZ) reporter gene assays for $>$1000 variants of the LacI/GalR chimeras and LacI-11. Lower values correspond to tighter repression of the lac operon. The first bar in each graph is labeled DEL (black) and shows β-galactosidase activity in the absence of repressor protein. Below 13 Miller units (solid black line), any change in repression altered bacterial growth [17]. The red dashed lines indicate the activities of un-mutated, starting proteins. Note that some red lines obscure the black lines in some panels. Error bars are the standard deviation of 2-4 independent bacterial colonies, each in quadruplicate or duplicate. All variants showed expressed and active protein in vivo, as assessed by the DNA pull-down assay.

In Figures S1-12, data are organized to show all variants at a given position (e.g. position 46) in all mutated proteins on one page. Assays were carried out in the absence and presence of allosteric effectors [17]. For all inducible repressors, the front colored series shows repression in the absence of effector and the back gray series show repression in the presence effector. For the co-repressible chimeras based on PurR (LLhP, LPhP57cs, LGhP), the front series shows repression in the presence of effector and the back series shows repression in the absence. LLhA variants have no known allosteric effectors.

Exceptions to the general description: (1) Values for the LLhA/Q55A variants R51L, R51M, and V52L were only determined from one days assay (2 colonies each in duplicate). (2) The LGhG variants H48I and H48N are not shown on the following plots but had white phenotypes (tight repression) in plate assays. In liquid culture assays, these variants appeared to be toxic to *E. coli*. Plate assays results were taken into consideration when assigning rheostat behavior to position 48 in LGhG (Table 4 in the main document).

Position 46

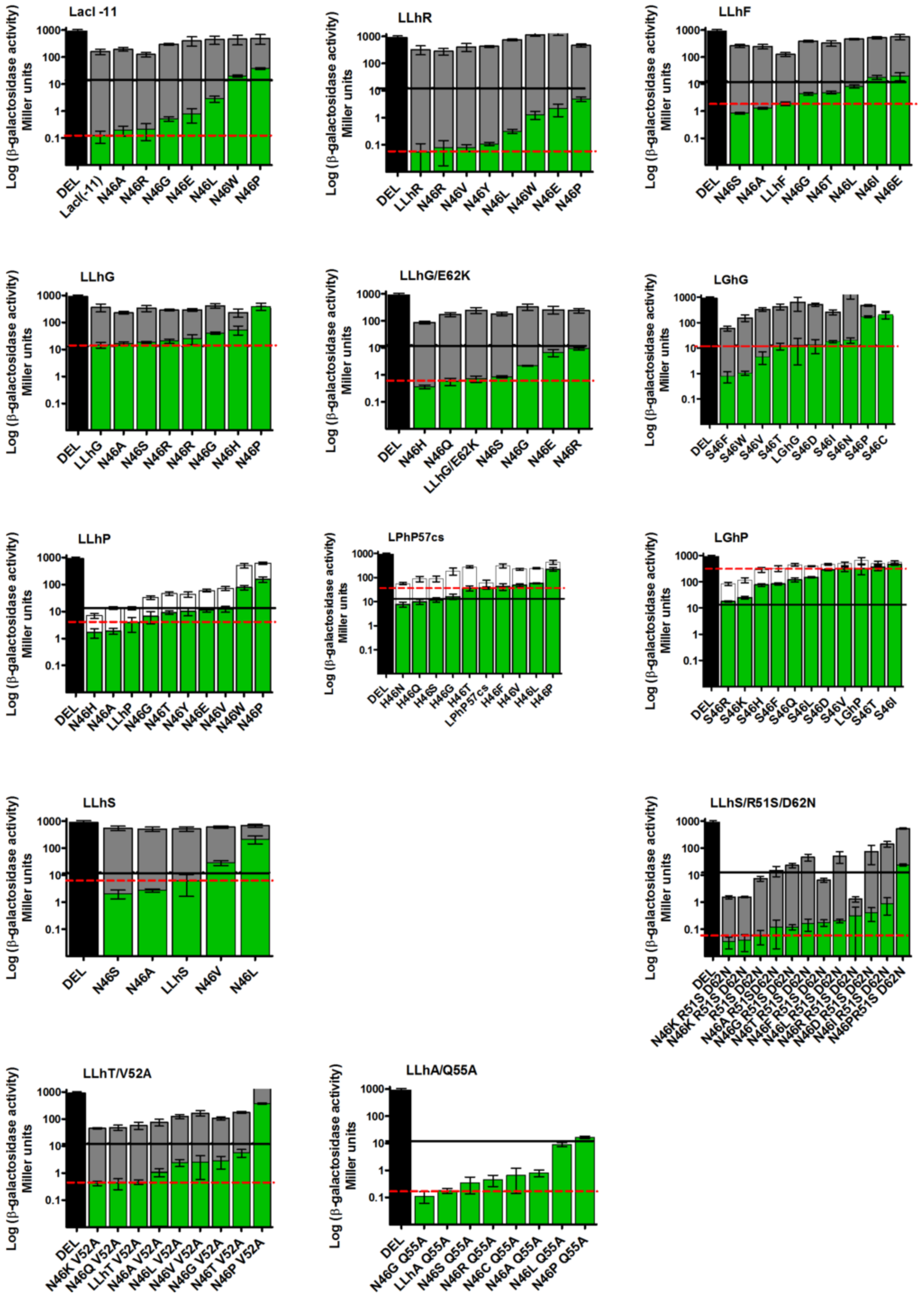

Figure S1: Beta-galactosidase reporter gene assay: Position 46

Position 48

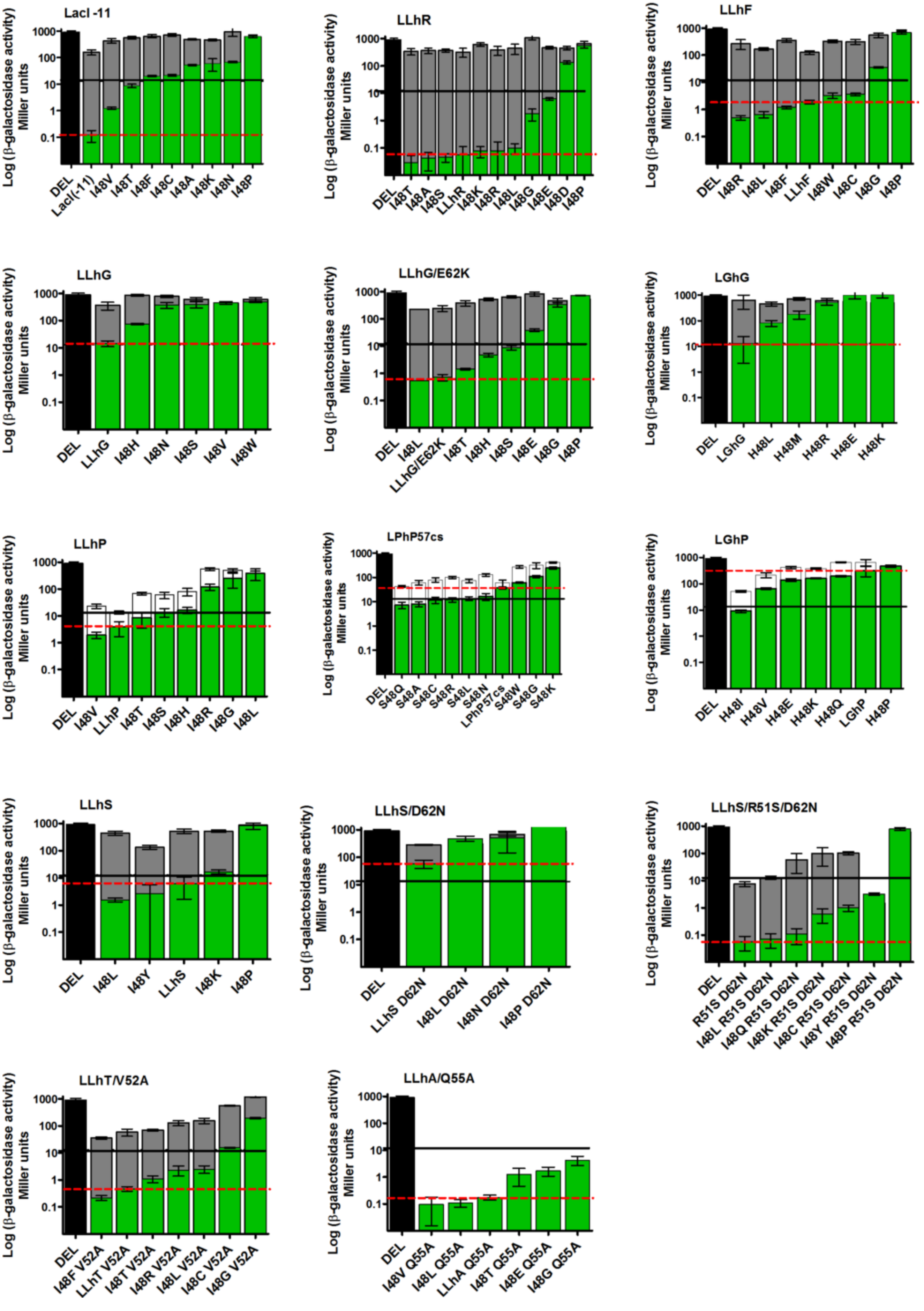

Figure S2: Beta-galactosidase reporter gene assay: Position 48

Position 50

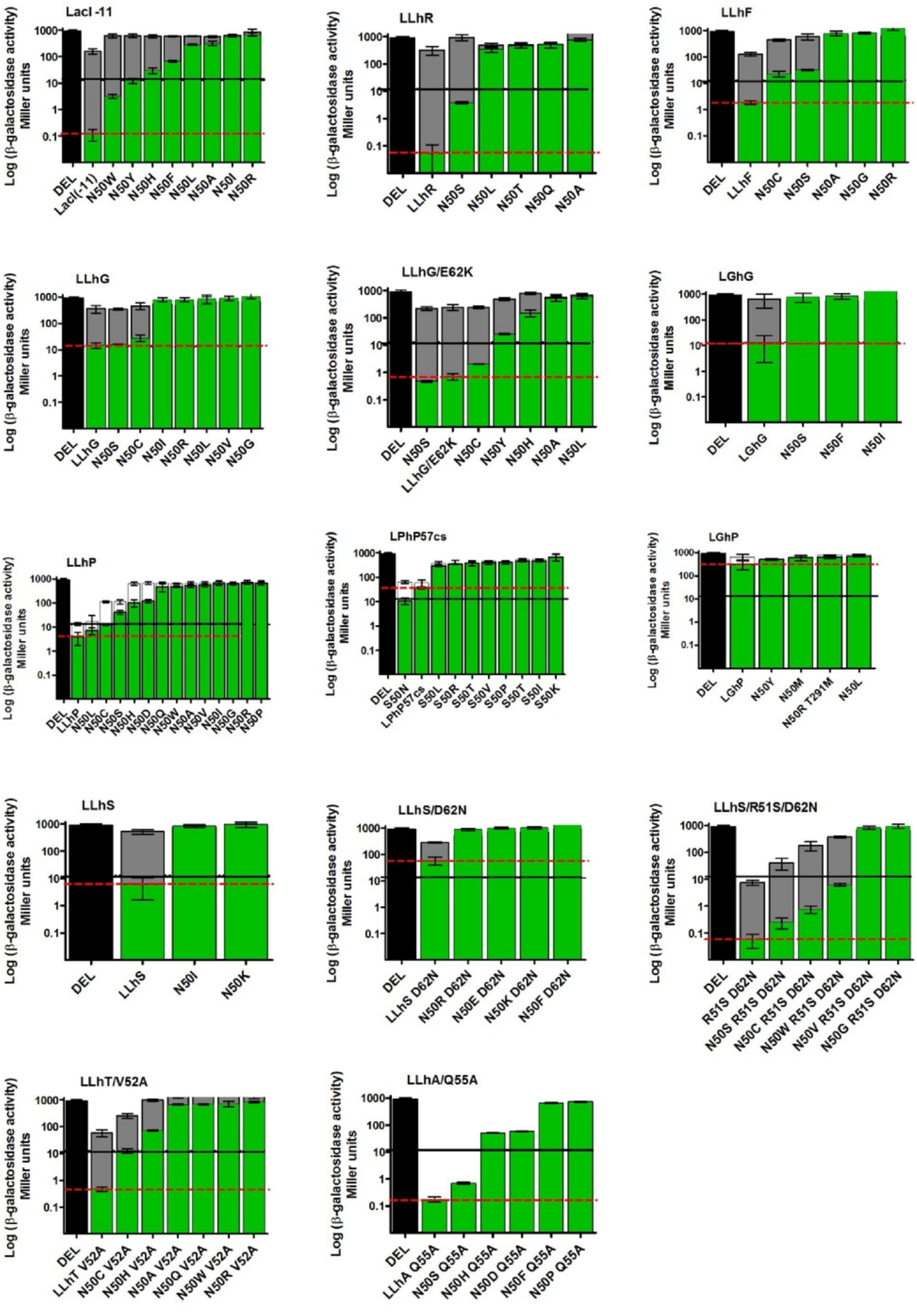

Figure S3: Beta-galactosidase reporter gene assay: Position 50

Position 51

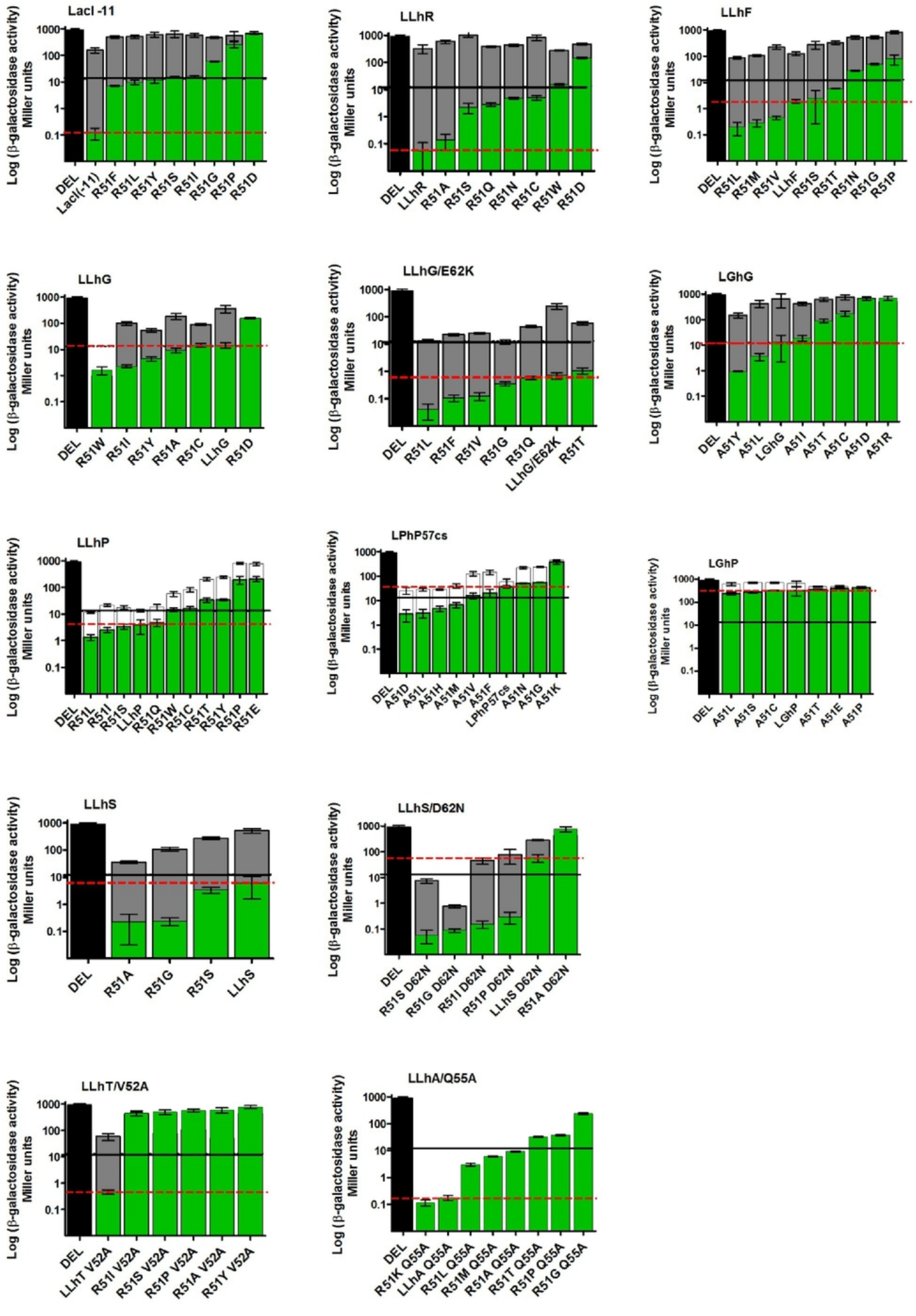

Figure S4: Beta-galactosidase reporter gene assay: Position 51

Position 52

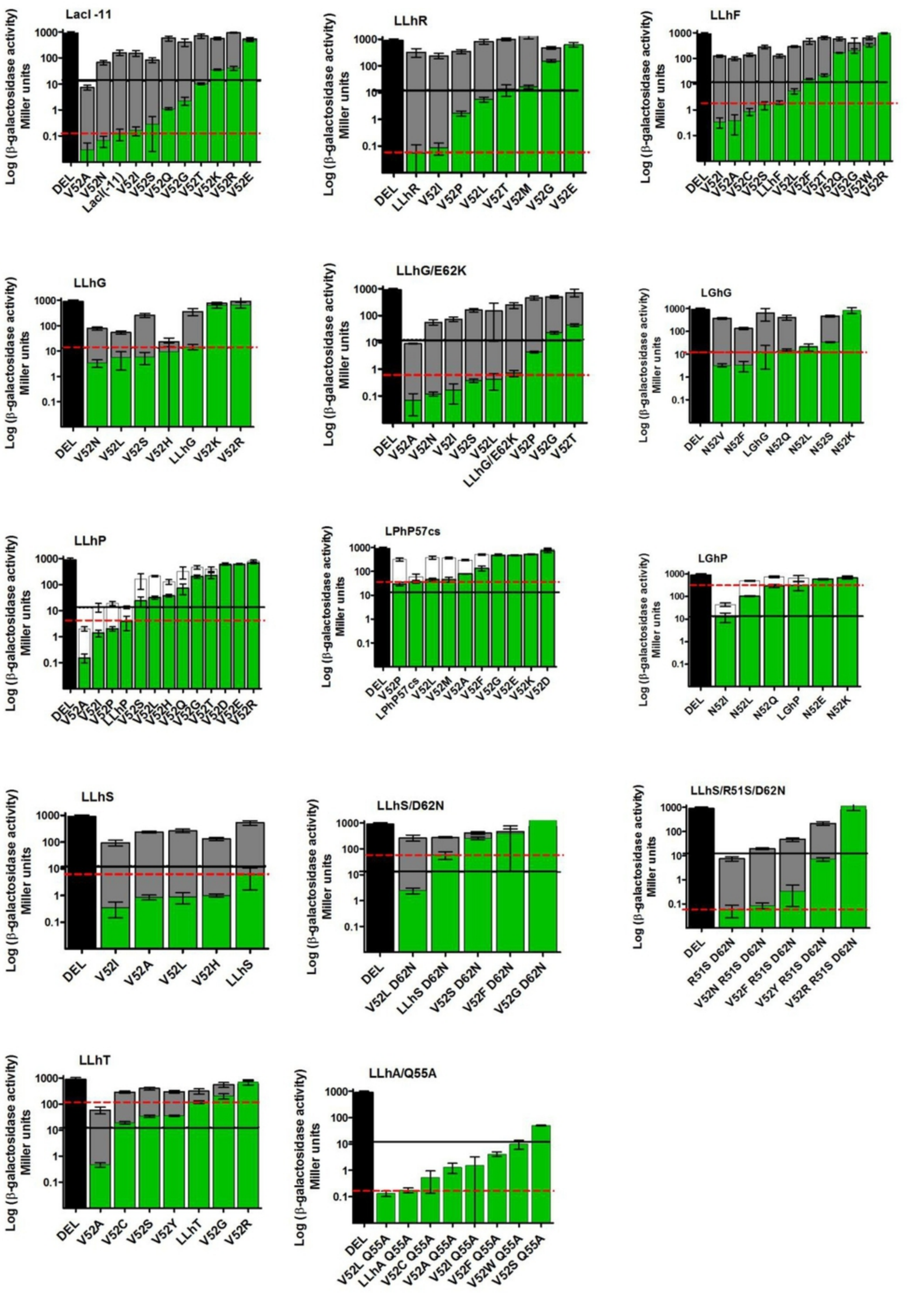

Figure S5: Beta-galactosidase reporter gene assay: Position 52

Position 54

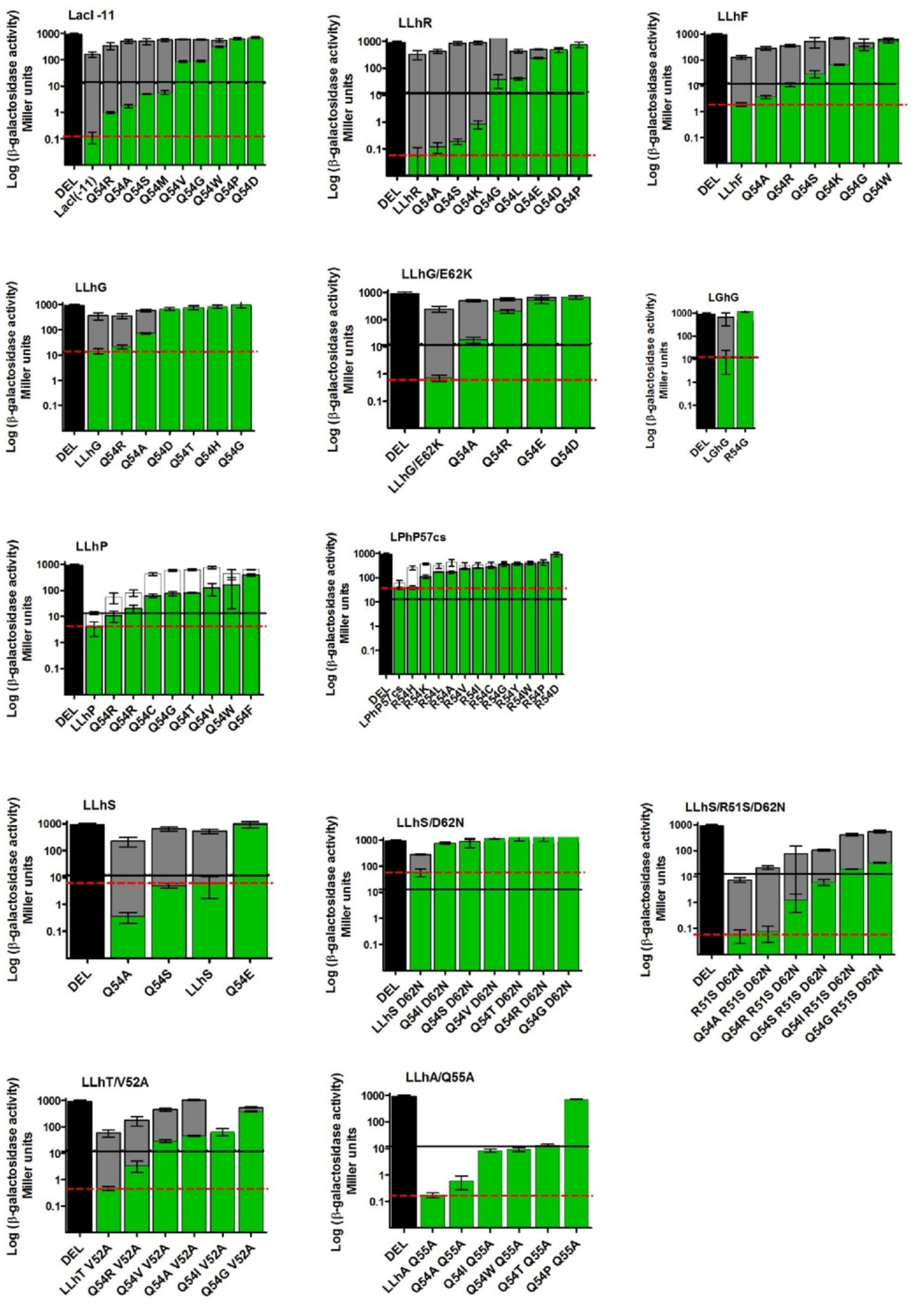

Figure S6: Beta-galactosidase reporter gene assay: Position 54

Position 55

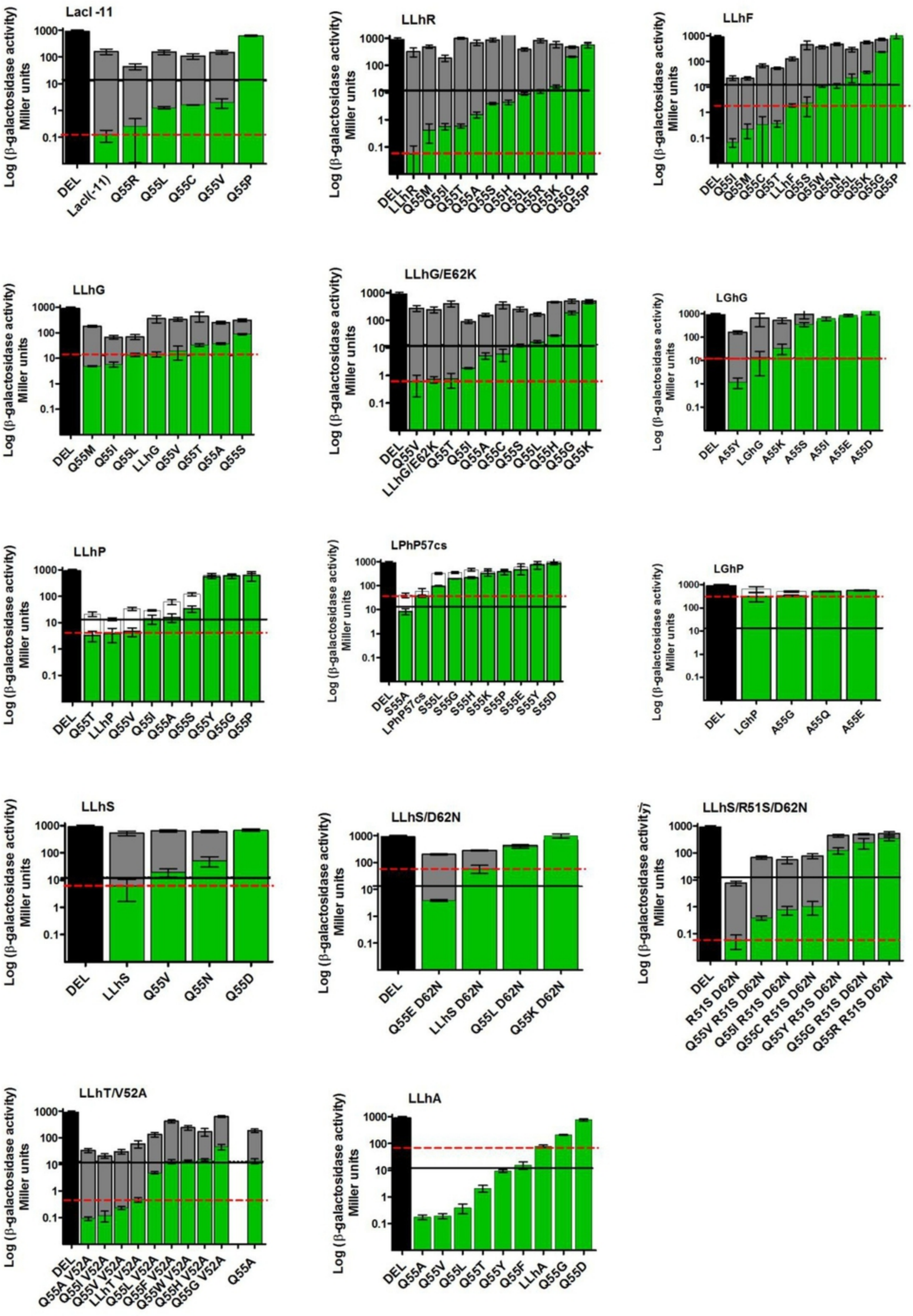

Figure S7: Beta-galactosidase reporter gene assay: Position 55

Position 58

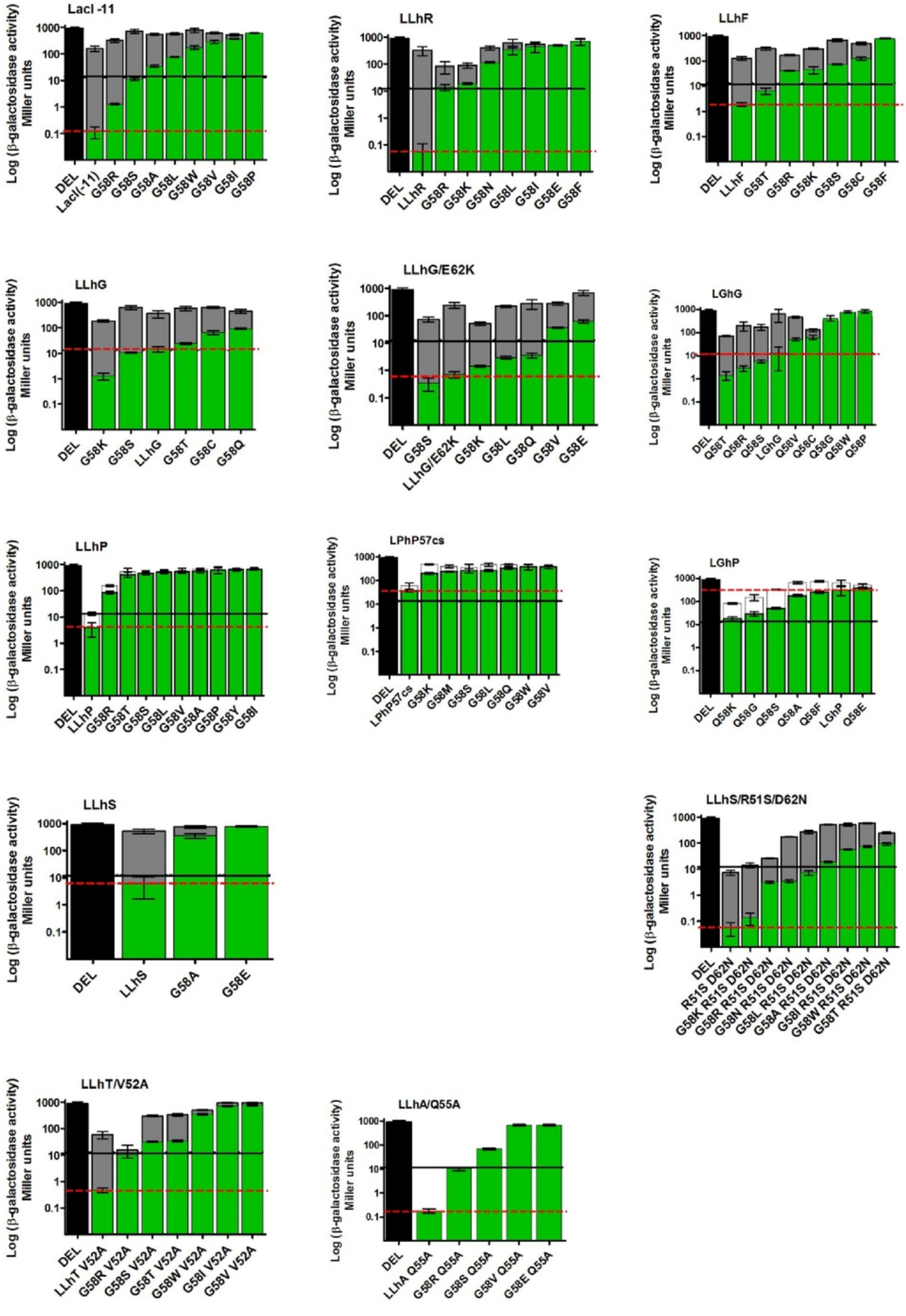

Figure S8: Beta-galactosidase reporter gene assay: Position 58

Position 59

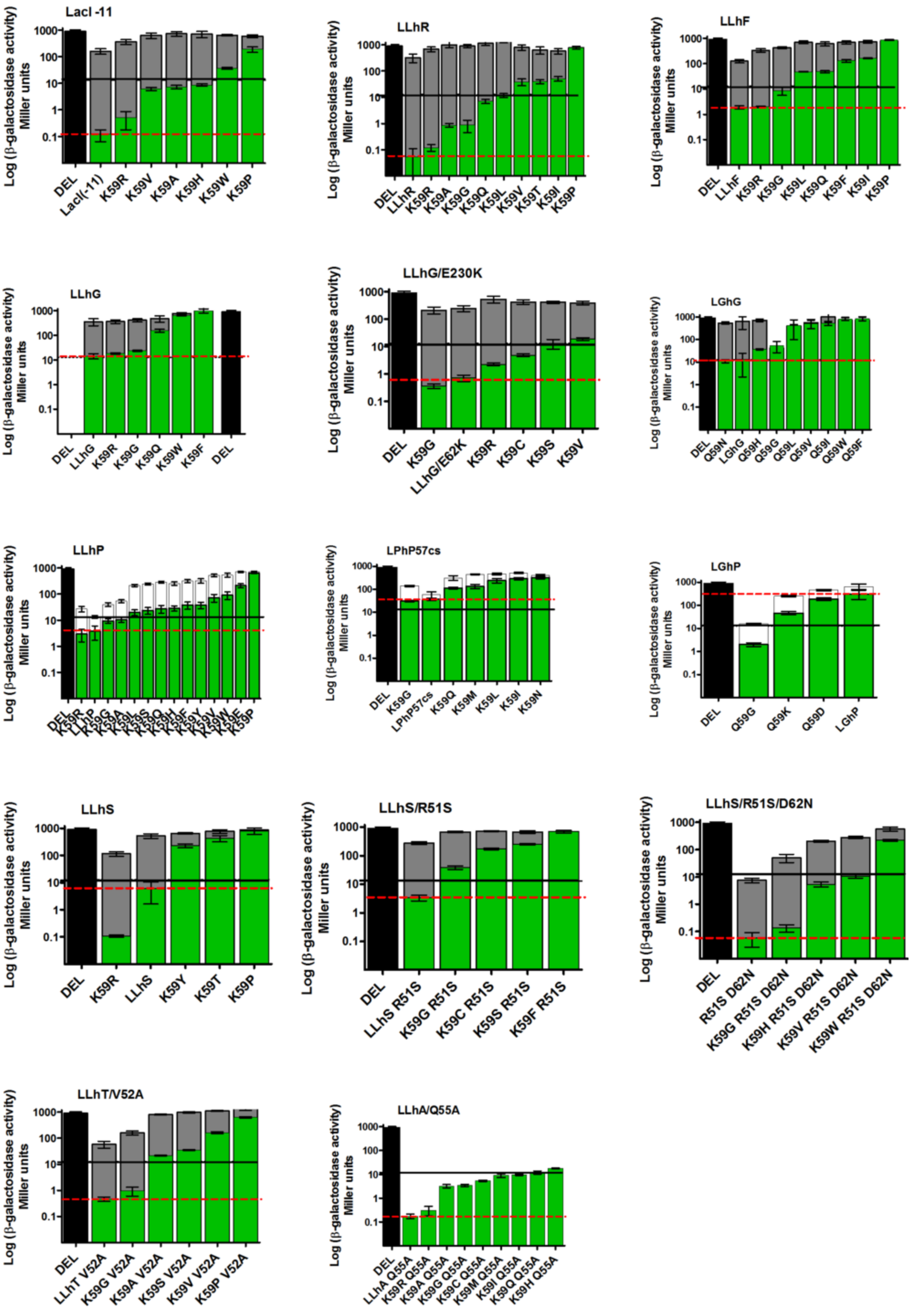

Figure S9: Beta-galactosidase reporter gene assay: Position 59

Position 60

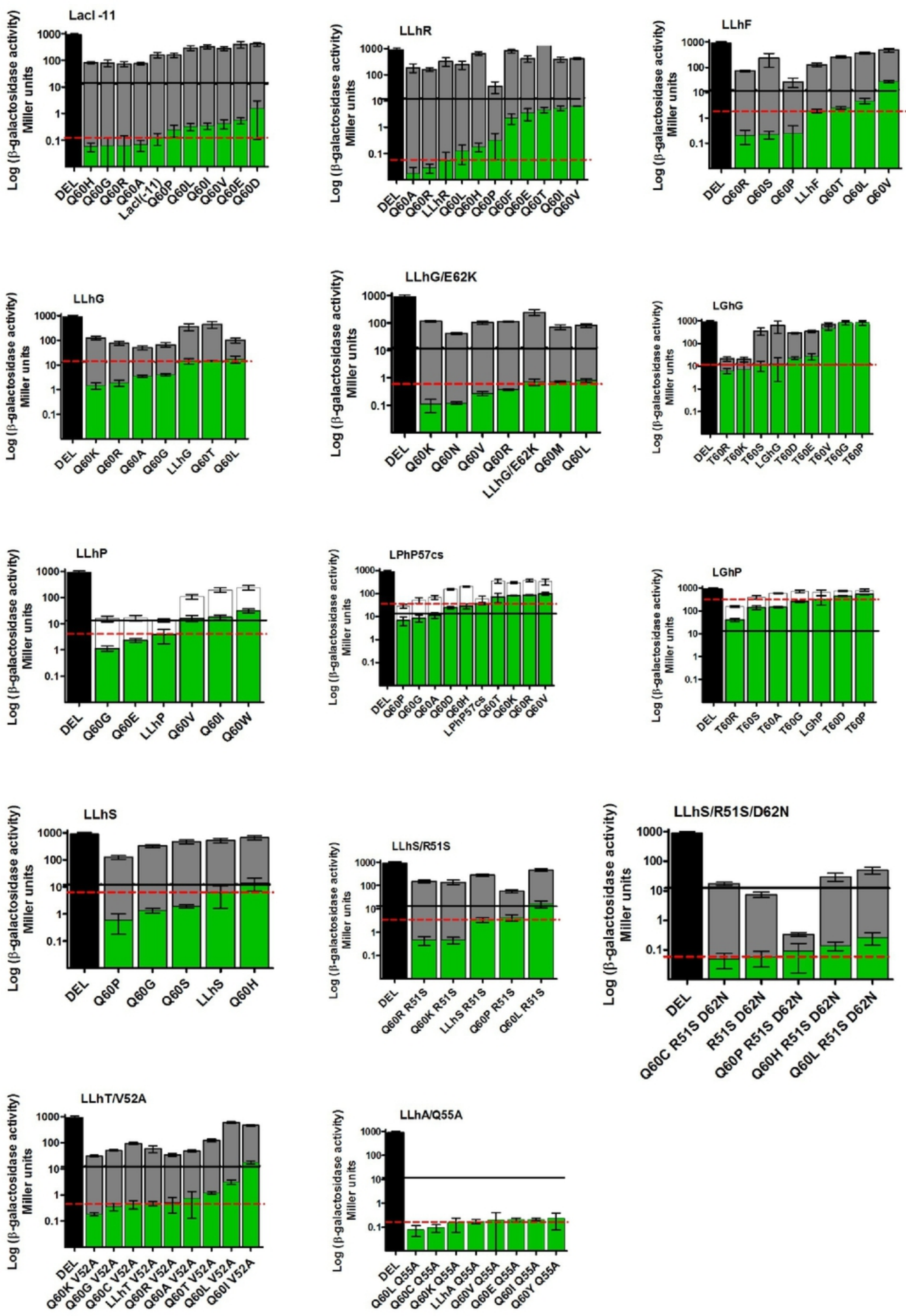

Figure S10: Beta-galactosidase reporter gene assay: Position 60

Position 61

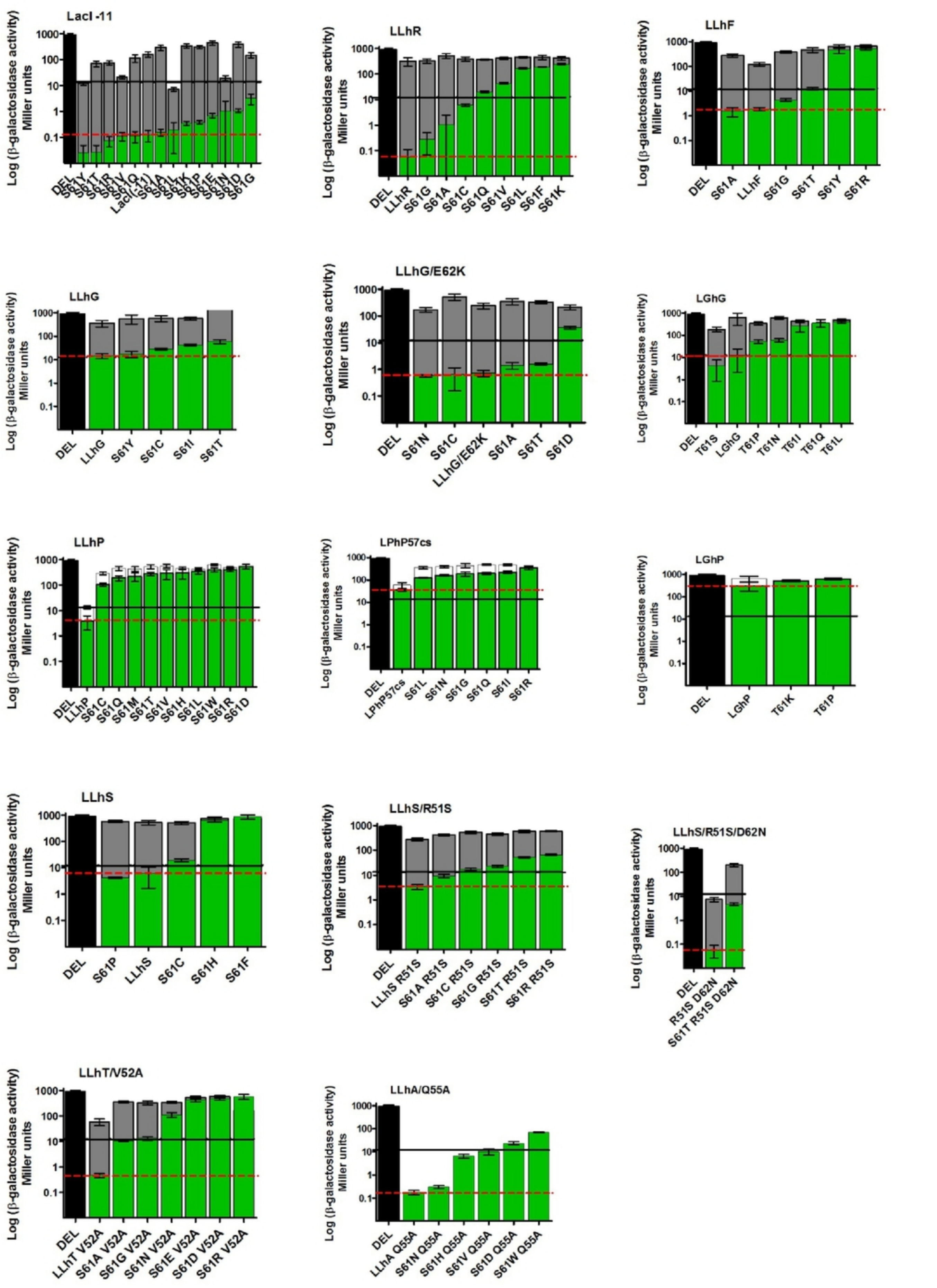

Figure S11: Beta-galactosidase reporter gene assay: Position 61

Position 62

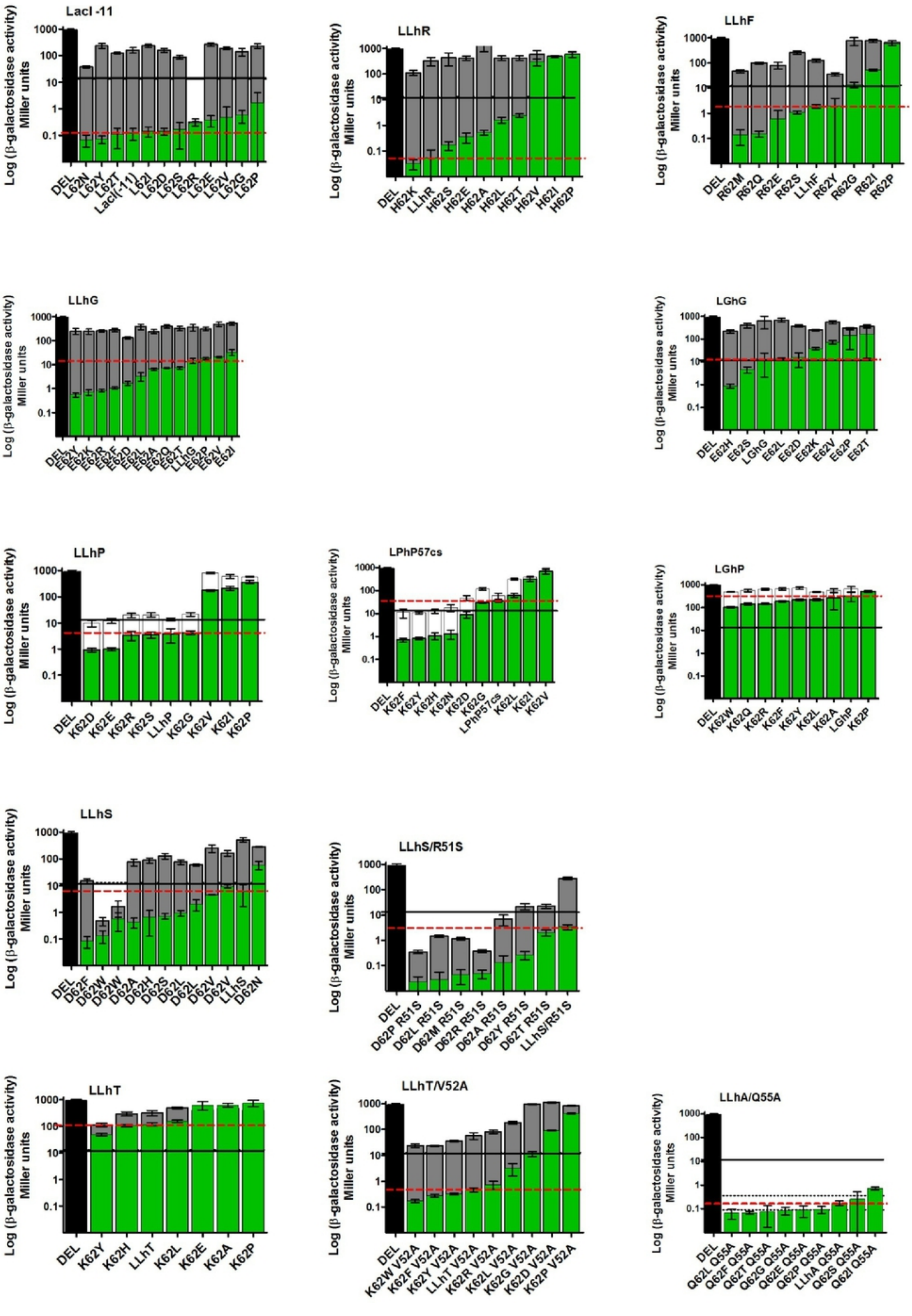

Figure S12: Beta-galactosidase reporter gene assay: Position 62
