# Supplementary material for: Rheostats and Toggle Switches for Modulating Protein Function
Source: PLoS One. 2013 Dec 30;8(12):e83502. doi: 10.1371/journal.pone.0083502 (PMC3875437; doi:10.1371/journal.pone.0083502)
Supplement: Data S4 — Supporting figures. Figures S13–S24. Parallel amino acid substitutions: positions 46–62. These plots compare the outcomes of parallel amino acid substitutions among the LacI/GalR homologs. Results are organized to show all variants obtained at a position on one page; five amino acids are shown per panel, as indicated in the legends at the right. In the left-hand panels, data for each variant were normalized relative to the starting protein, which had different repression values (Table 1 in the main document). If an amino acid substitution caused no change, this corresponds to a value of 1 on the plots (dashed black line). As discussed in Methods, data within 2-fold are considered equivalent to each other, which is indicated by the straight dotted lines on the plots; this range is usually larger than the error bars of a repression measurement. Substitutions that enhanced repression have increased fold-change (>2). Substitutions that diminished repression have decreased fold-change (<2). The jagged dotted line labeled MIN shows the no repressor DEL control relative to the starting protein and represents the lowest possible value for each homolog. The colored connecting lines are to aid visual inspection of the data. The right-hand panels show amino acid frequency in the naturally occurring proteins at the relevant positions, as calculated from the YPAL-MSA. The following definitions were used to assign the results in Table 5: “A” = amino acid absent from the YPAL-MSA but substitution allowed repression near the parent value or better in two or more chimeras; “P1” = amino acid present in the YPAL-MSA but substitution diminished repression below the biologically determined threshold of 13 Miller units (not shown) for at least one chimera; “P2” = amino acid present in the YPAL-MSA but substitution diminished repression to MIN; these designations were only used for parent chimeras with repression better than 13 Miller units; “D” = substitution caused widely different outc [file pone.0083502.s004.pdf]

Supplemental Material for:
Rheostats and toggle switches for modulating protein function
File 4: Parallel amino acid substitutions

Meinhardt, Manley, Parente and Swint-Kruse

List of Figures

S13 Parallel amino acid substitutions: Position 46 3
S14 Parallel amino acid substitutions: Position 48 4
S15 Parallel amino acid substitutions: Position 50 5
S16 Parallel amino acid substitutions: Position 51 6
S17 Parallel amino acid substitutions: Position 52 7
S18 Parallel amino acid substitutions: Position 54 8
S19 Parallel amino acid substitutions: Position 55 9
S20 Parallel amino acid substitutions: Position 58 10
S21 Parallel amino acid substitutions: Position 59 11
S22 Parallel amino acid substitutions: Position 60 12
S23 Parallel amino acid substitutions: Position 61 13
S24 Parallel amino acid substitutions: Position 62 14

Figures S13-S24. These plots compare the outcomes of parallel amino acid substitutions among the LacI/GalR homologs. Results are organized to show all variants obtained at a position on one page; five amino acids are shown per panel, as indicated in the legends at the right.

In the left-hand panels, data for each variant were normalized relative to the starting protein, which had different repression values (Table 1 in the main document). If an amino acid substitution caused no change, this corresponds to a value of 1 on the plots (dashed black line). As discussed in Methods, data within 2-fold are considered equivalent to each other, which is indicated by the straight dotted lines on the plots; this range is usually larger than the error bars of a repression measurement. Substitutions that enhanced repression have increased fold-change (>2). Substitutions that diminished repression have decreased fold-change (<2). The jagged dotted line labeled MIN shows the no repressor DEL control relative to the starting protein and represents the lowest possible value for each homolog. The colored connecting lines are to aid visual inspection of the data.

The right-hand panels show amino acid frequency in the naturally occurring proteins at the relevant positions, as calculated from the YPAL-MSA. The following definitions were used to assign the results in Table 5:

- A = amino acid absent from the YPAL-MSA but substitution allowed repression near the parent value or better in two or more chimeras.
- P1 = amino acid present in the YPAL-MSA but substitution diminished repression below the biologically determined threshold of 13 Miller units (not shown) for at least one chimera.
- P2 = amino acid present in the YPAL-MSA but substitution diminished repression to MIN; these designations were only used for parent chimeras with repression better than 13 Miller units.
- D = substitution caused widely different outcomes among several chimeras.
- L = absent in the YPAL- MSA but allowed strong repression in LacI-11; LacI data are commonly used as a single representative of the family for benchmarking MSA analyses.

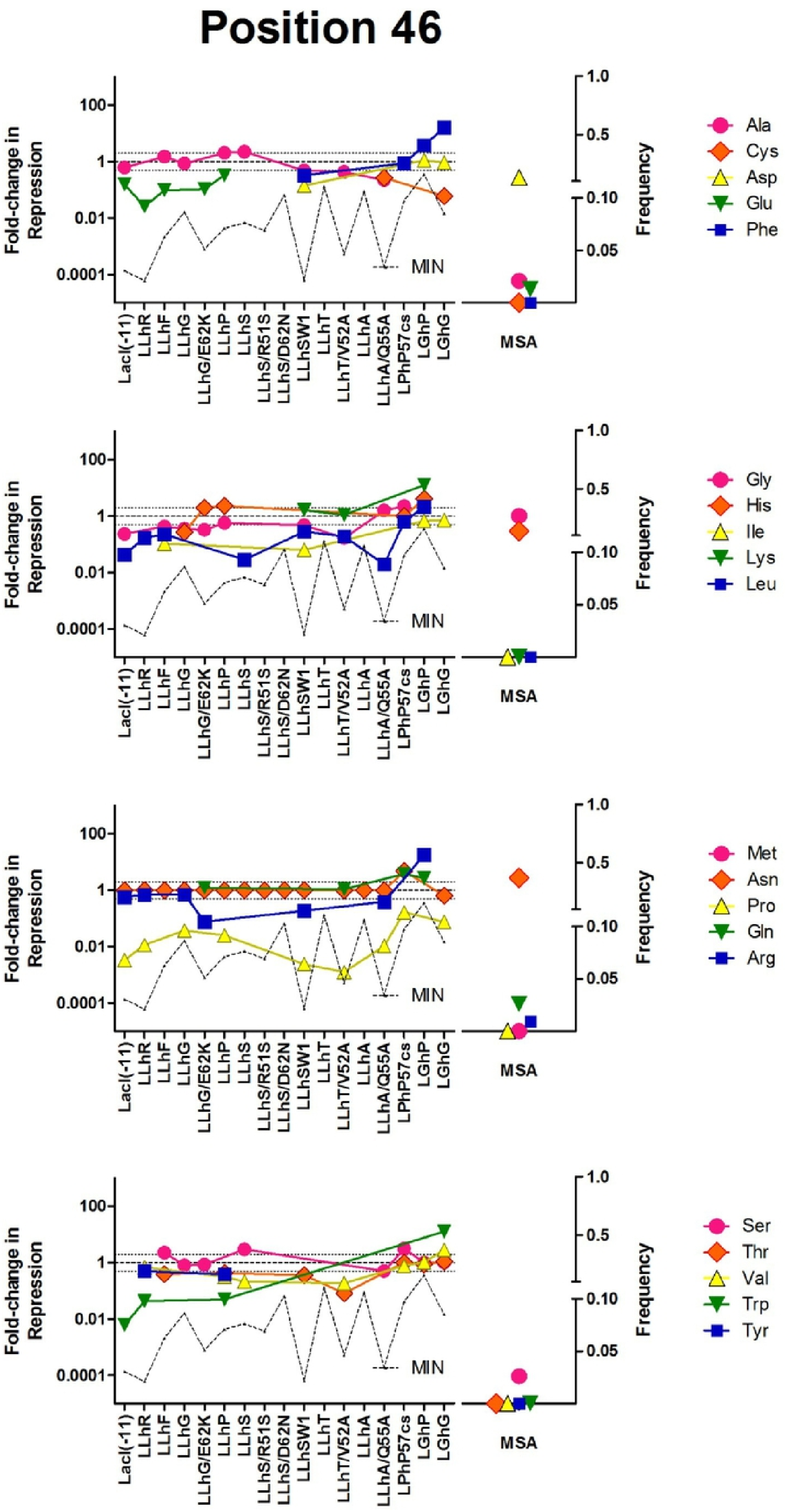

Figure S13: Parallel amino acid substitutions: Position 46

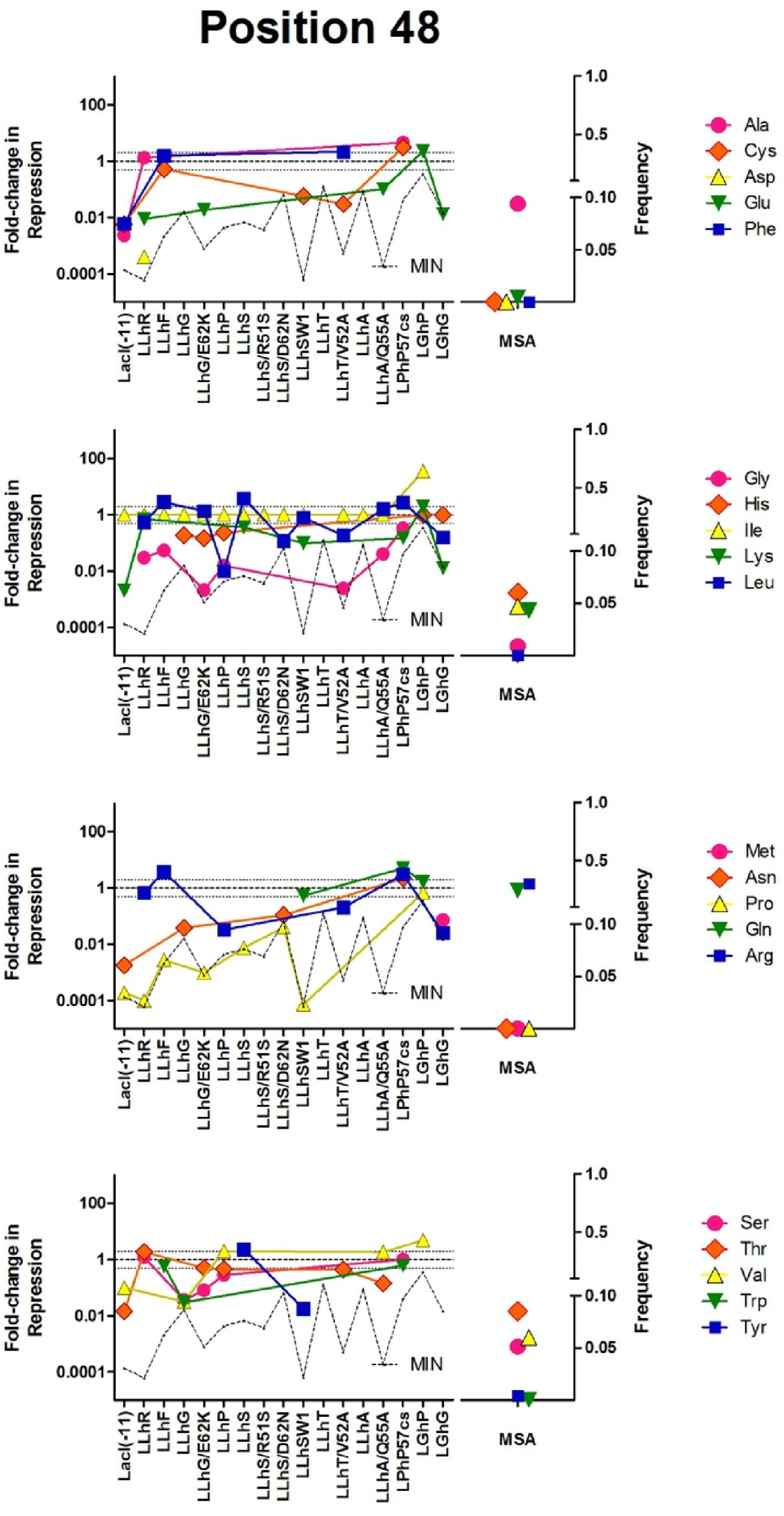

Figure S14: Parallel amino acid substitutions: Position 48

Position 50

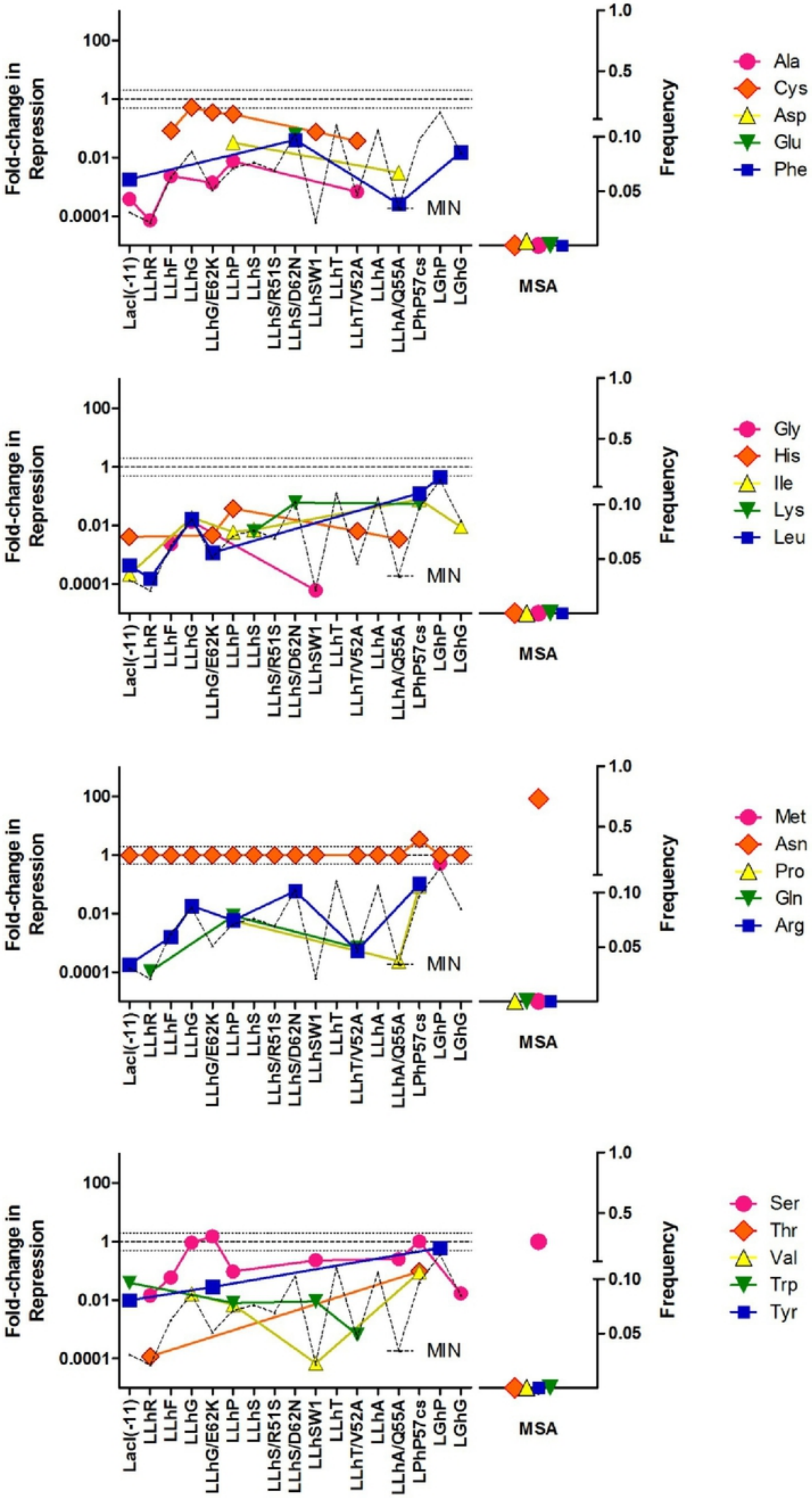

Figure S15: Parallel amino acid substitutions: Position 50

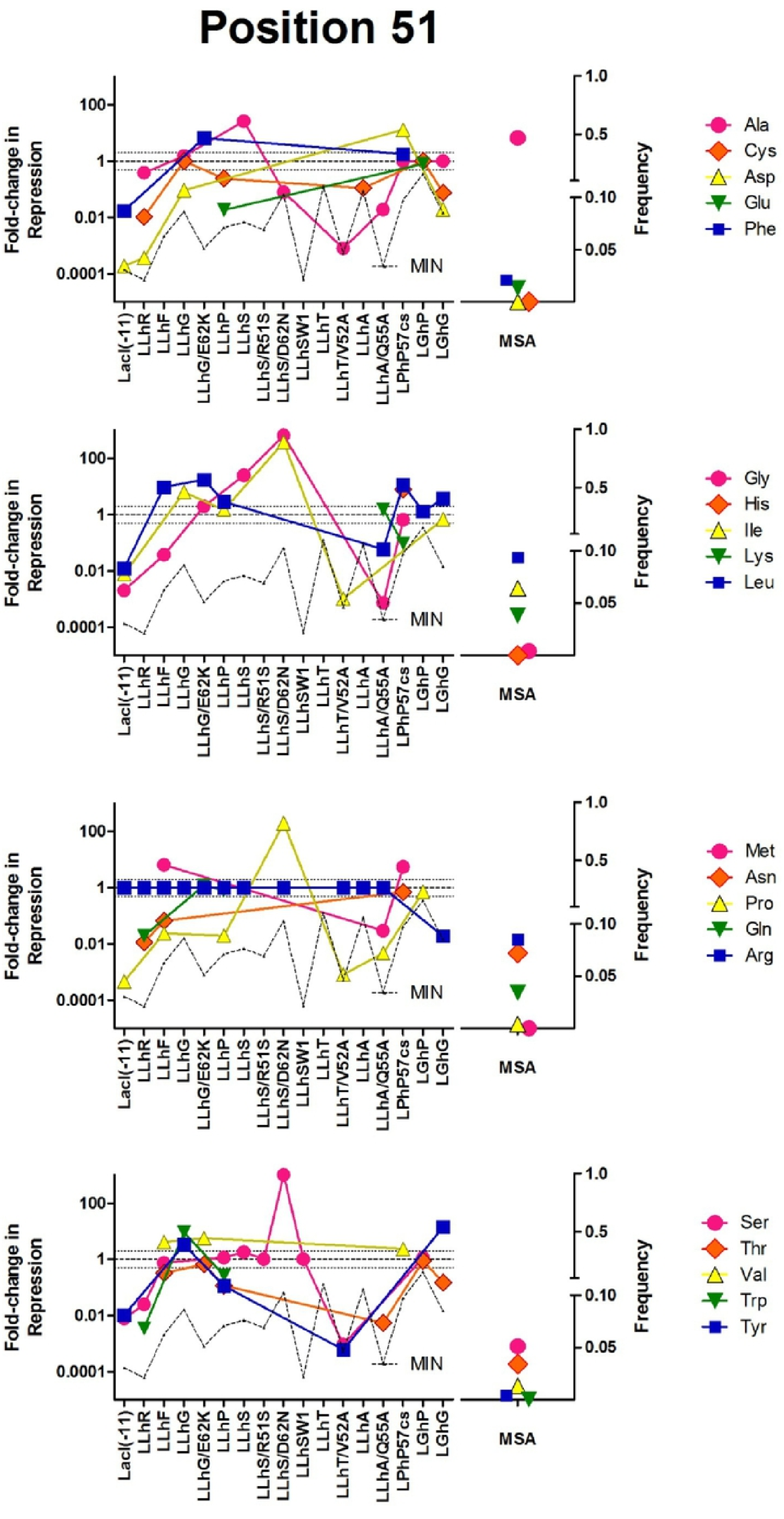

Figure S16: Parallel amino acid substitutions: Position 51

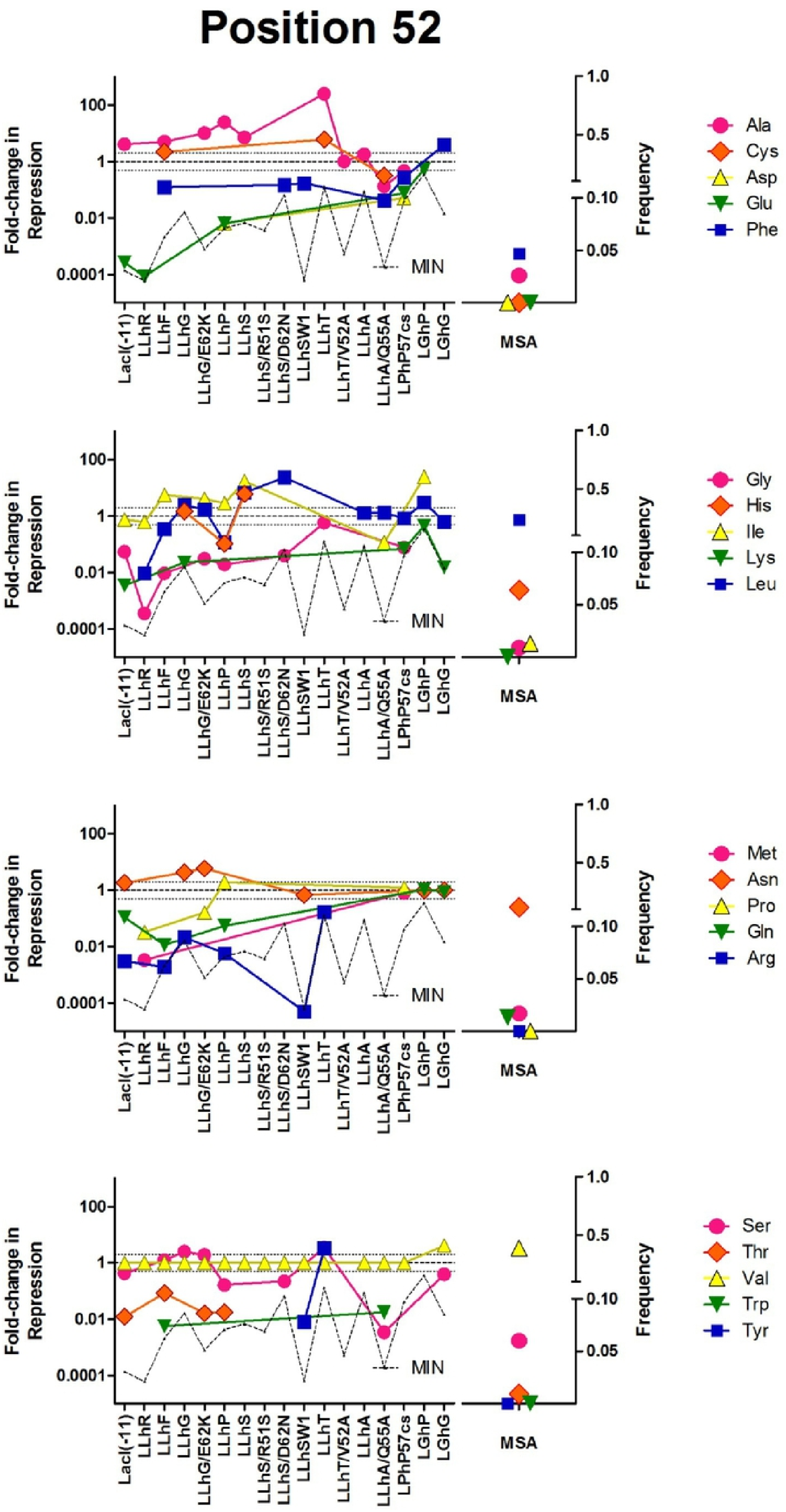

Figure S17: Parallel amino acid substitutions: Position 52

Position 54

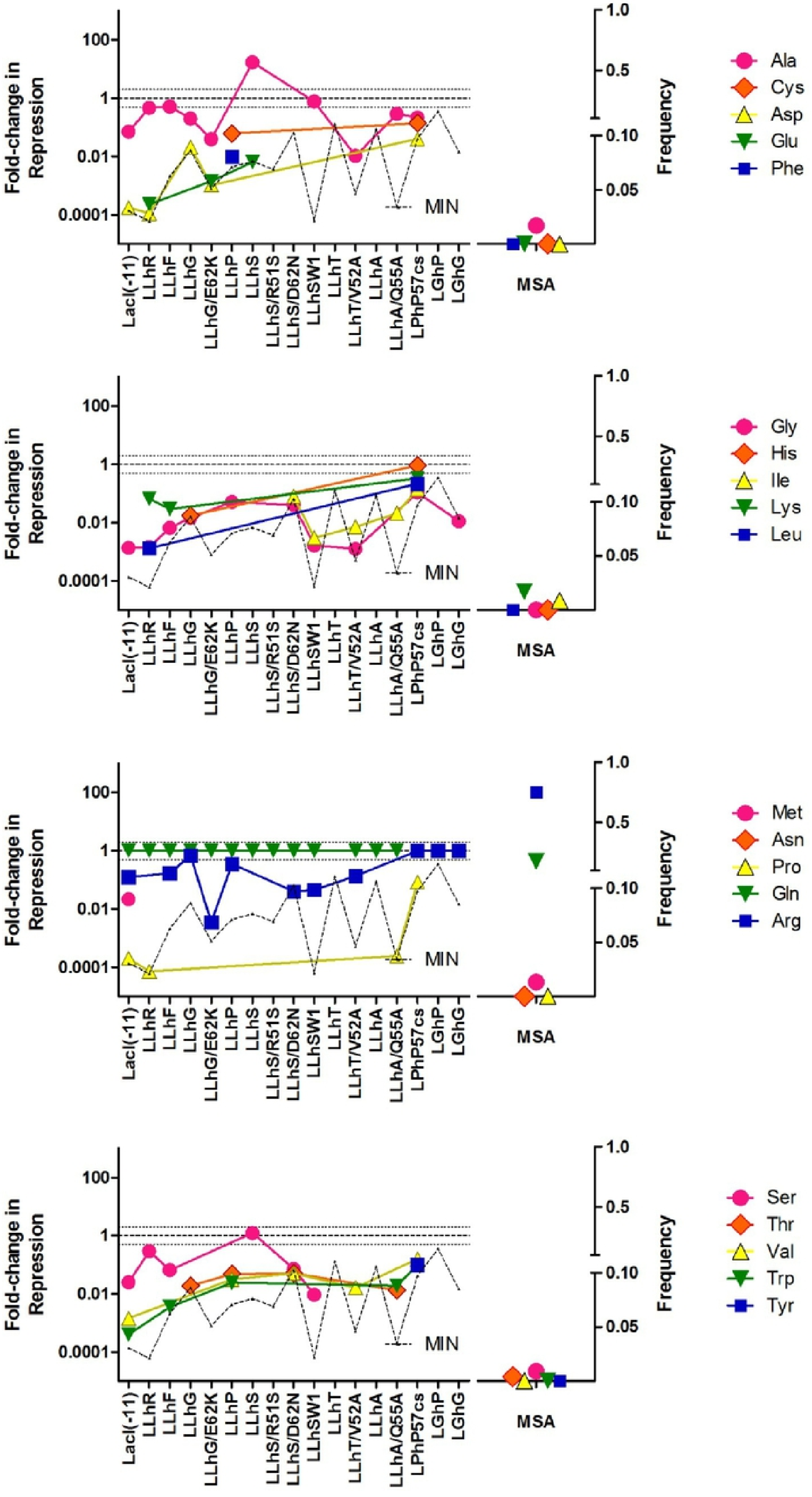

Figure S18: Parallel amino acid substitutions: Position 54

Position 55

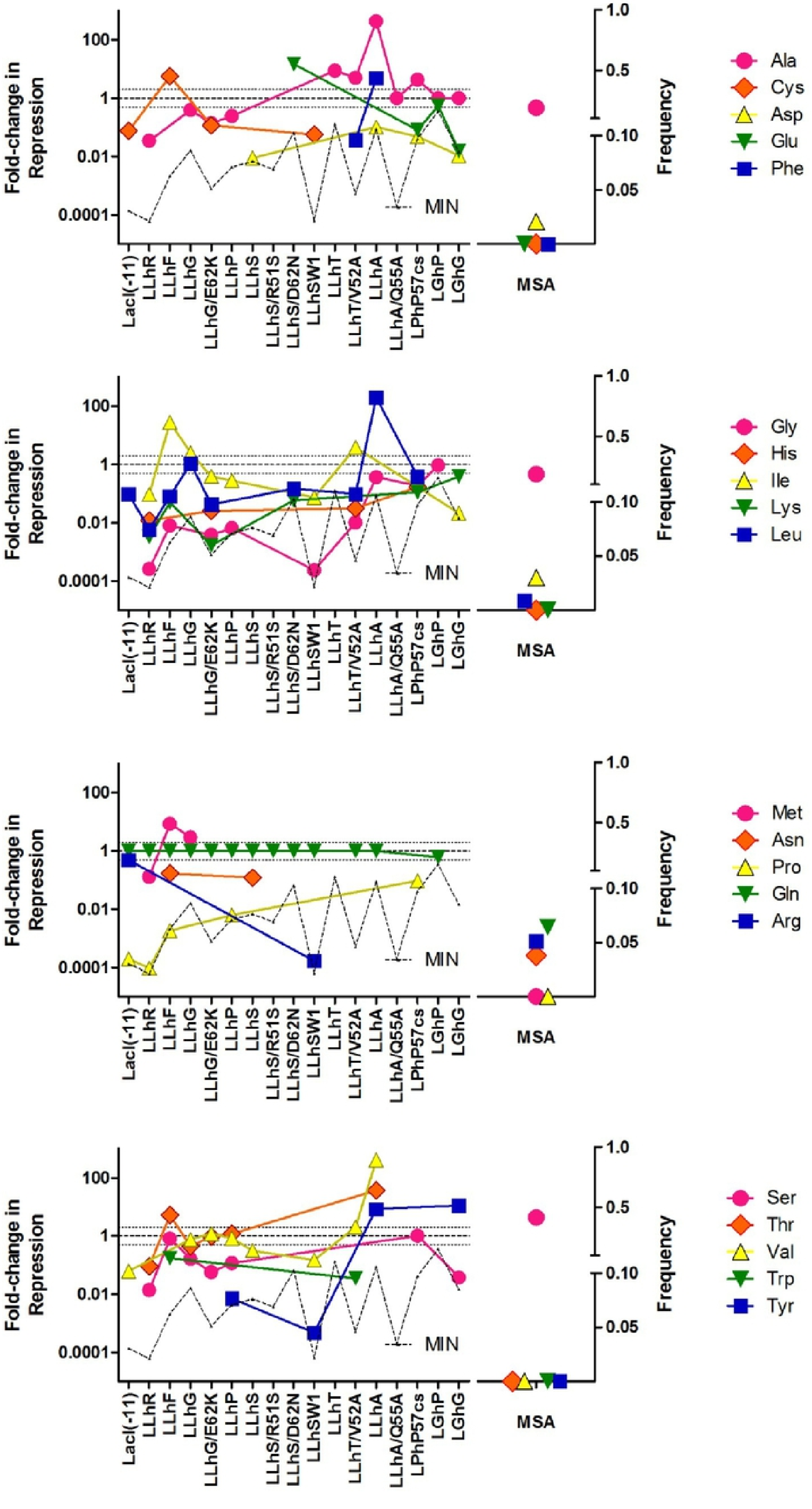

Figure S19: Parallel amino acid substitutions: Position 55

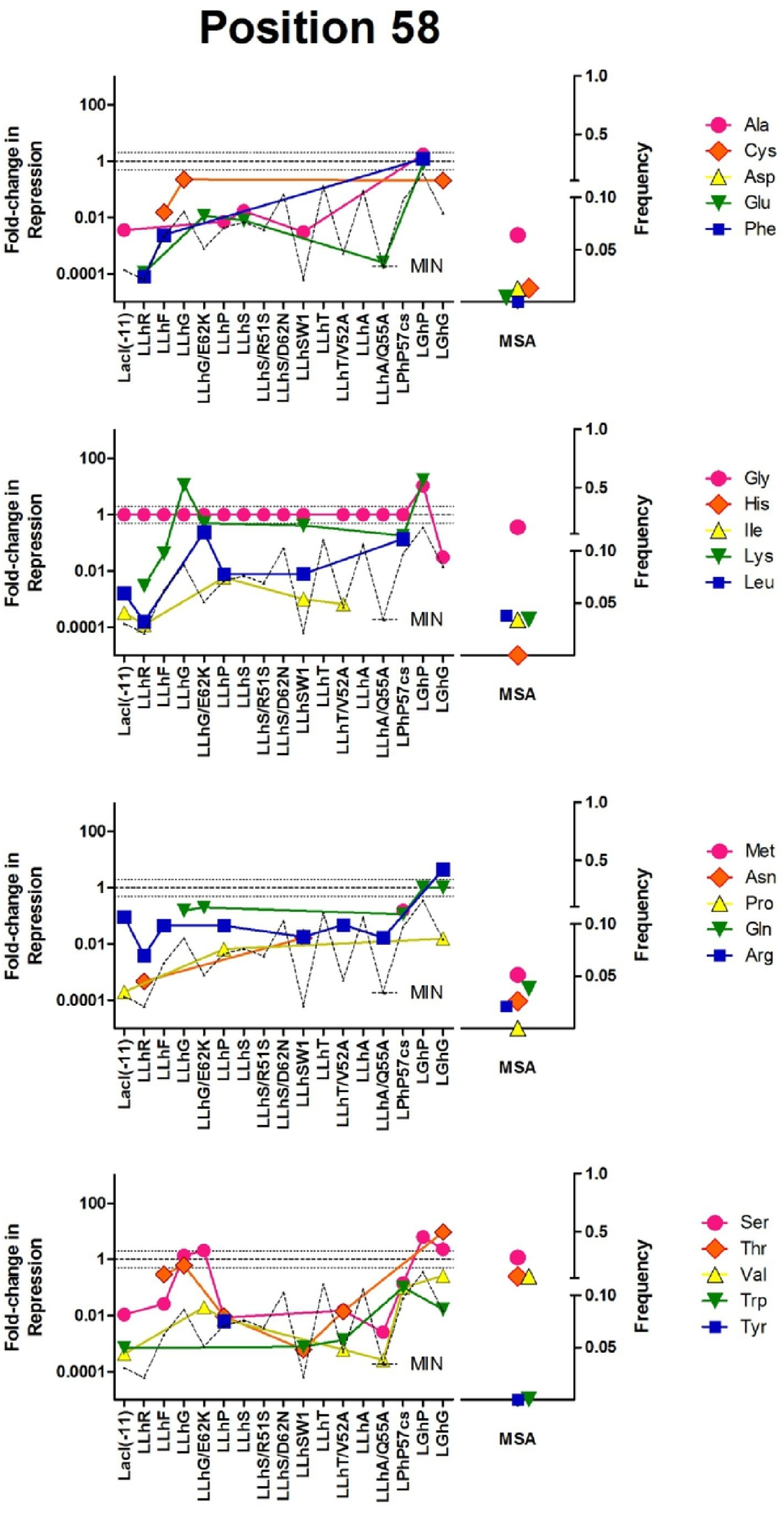

Figure S20: Parallel amino acid substitutions: Position 58

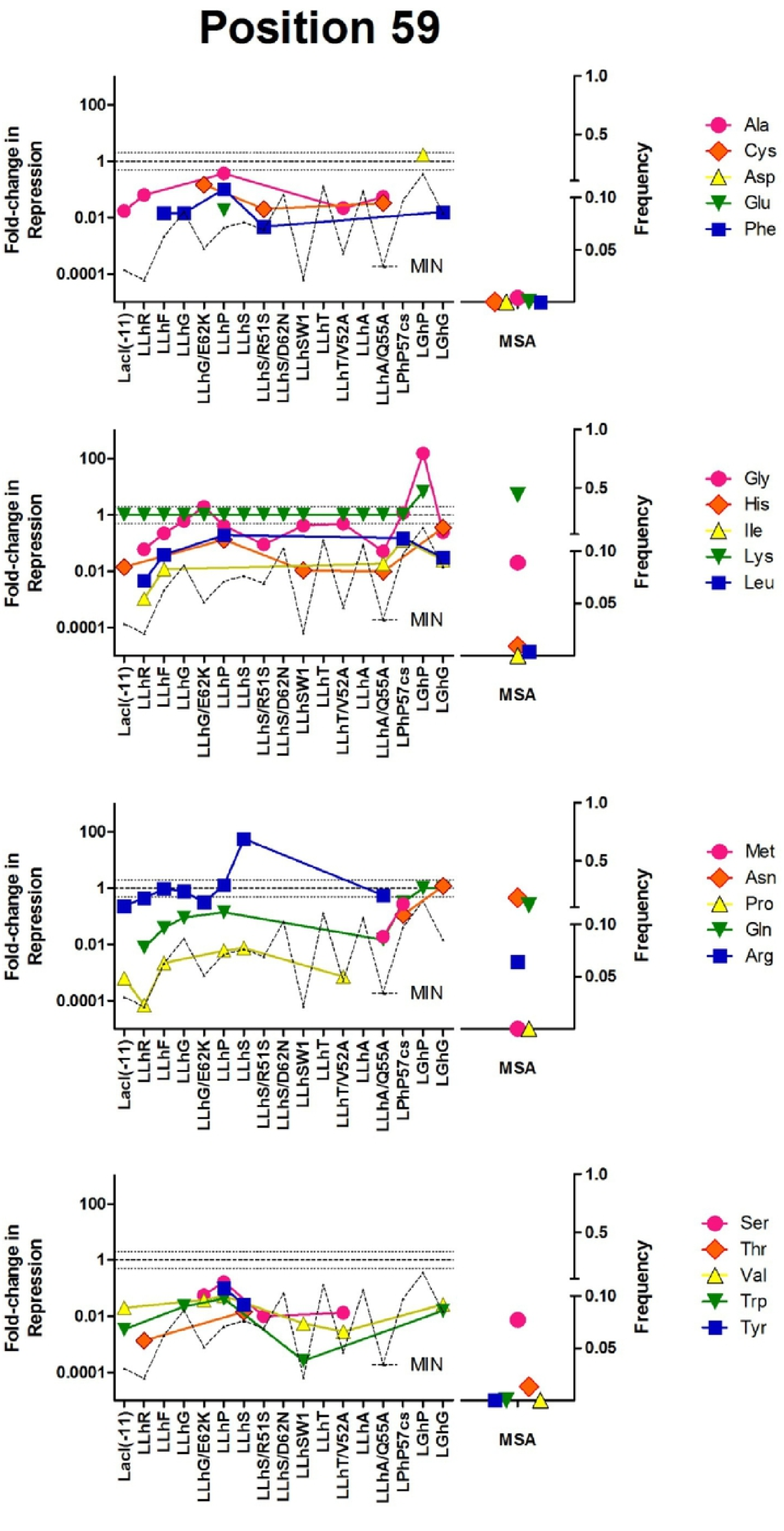

Figure S21: Parallel amino acid substitutions: Position 59

Position 60

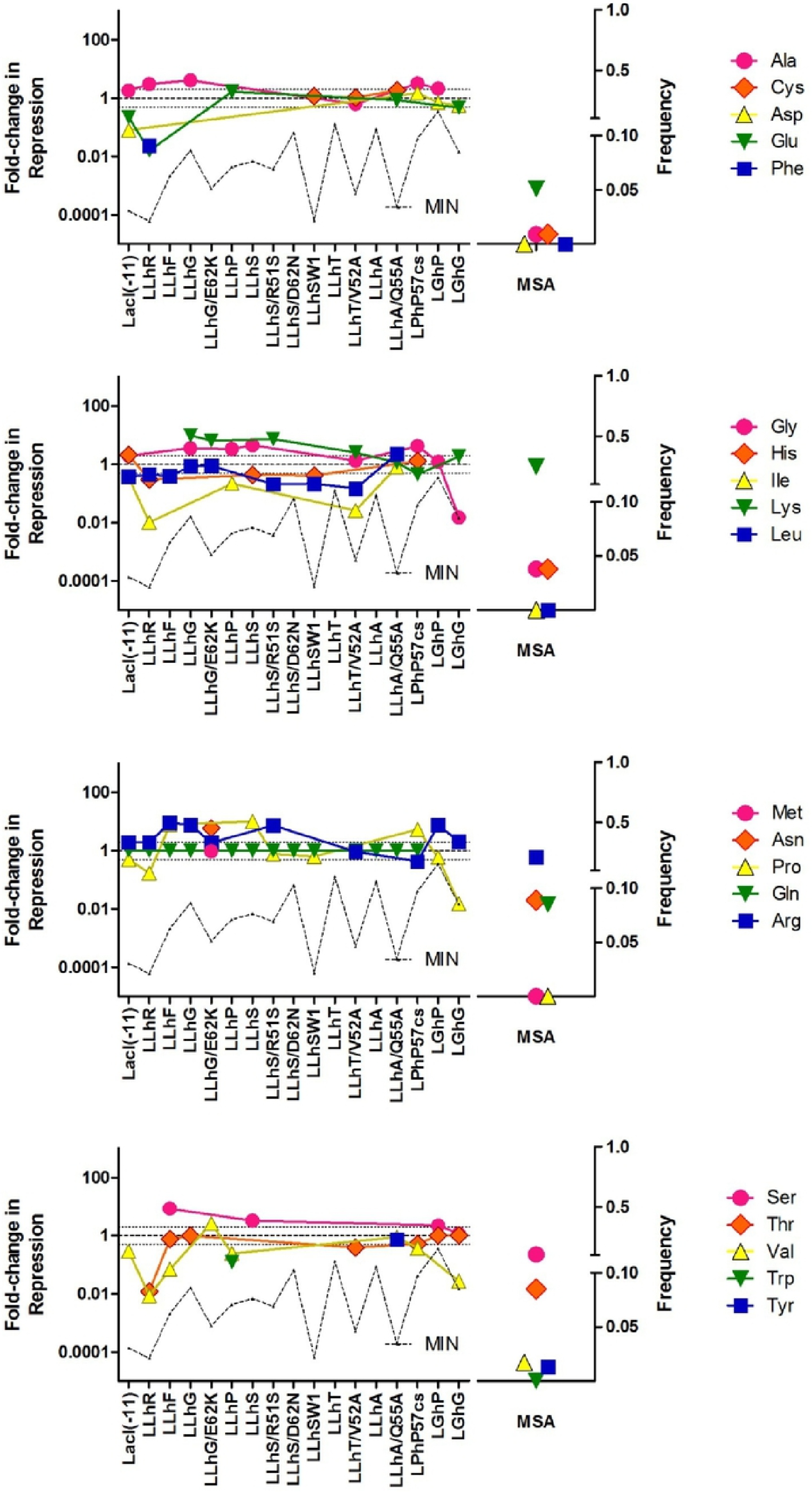

Figure S22: Parallel amino acid substitutions: Position 60

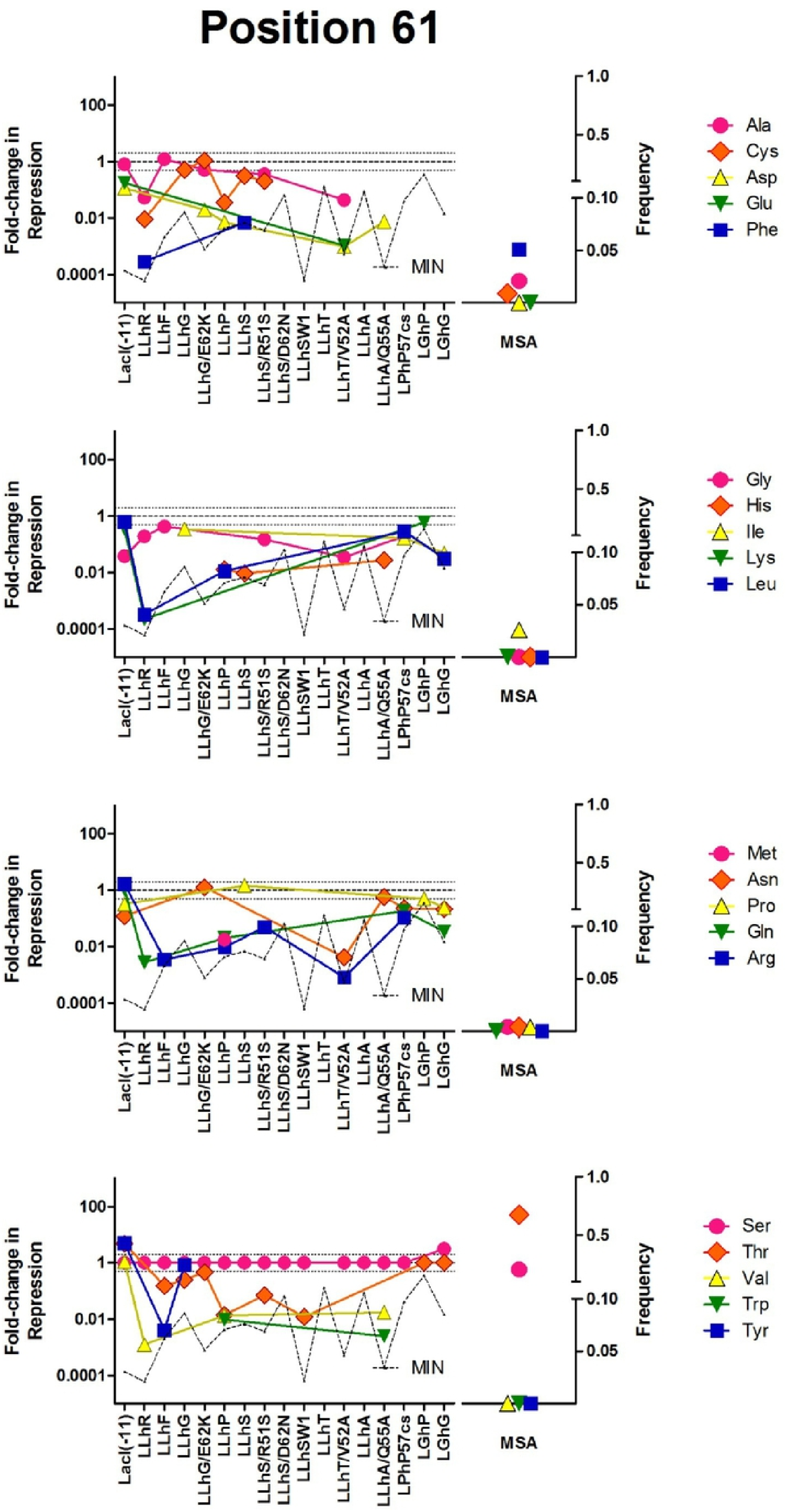

Figure S23: Parallel amino acid substitutions: Position 61

Position 62

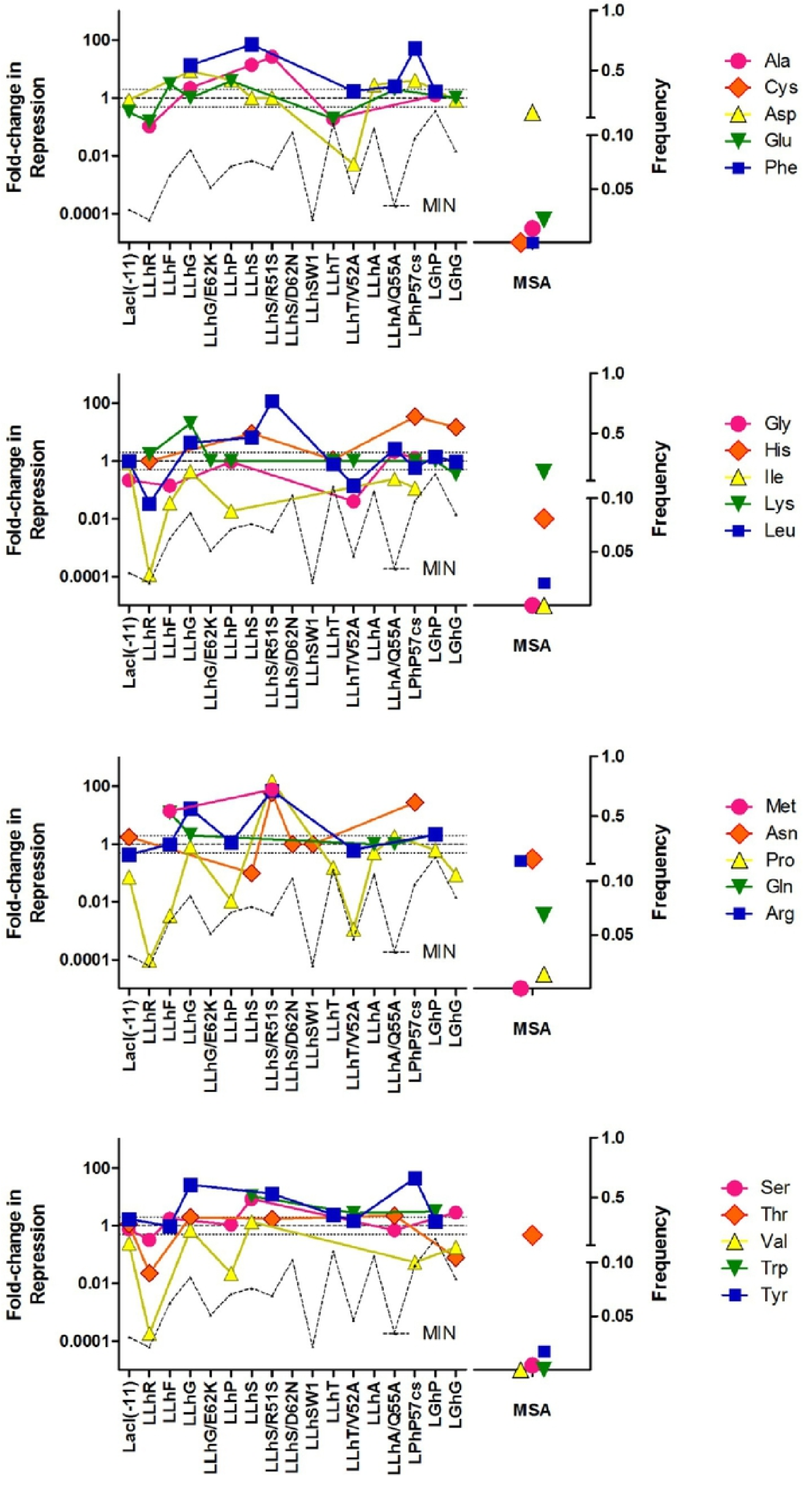

Figure S24: Parallel amino acid substitutions: Position 62
