# Supplementary material for: Rheostats and Toggle Switches for Modulating Protein Function
Source: PLoS One. 2013 Dec 30;8(12):e83502. doi: 10.1371/journal.pone.0083502 (PMC3875437; doi:10.1371/journal.pone.0083502)
Supplement: Data S5 — Supporting figures. Figures S25–S54. Physico-chemical trends: positions 46–54. Repression assay data for each position were color-coded according to various physico-chemical scales (Table S20 in Data S2). For example, Figures S25–S28 show results for position 46 color-coded by accessible surface area of the free side chain [46], side chain branching, charge/polarity/aromaticity, and hydrophobicity [45]. The relevant parameter can be determined from the legend in the lower right hand corner of each graph. Positions 50–58 have potential to participate in an alpha helix, and repression assay results were also compared to both average and position-specific helical propensities [47]. For simplicity, only one helix color scale is used as a legend, with magenta corresponding to high propensity and green corresponding to low propensity. Since wild-type LacI and PurR have different length helices [20], we compared multiple helical scales to results for each position. However, no scale showed good correlation with the functional assay among all chimeras. (PDF) [file pone.0083502.s005.pdf]

Supplemental Material for:
Rheostats and toggle switches for modulating protein function
File 5: Physicochemical trends: Positions 46-54

Meinhardt, Manley, Parente and Swint-Kruse

List of Figures

S25 Physicochemical trends: Position 46, Accessible surface area . . . 3
S26 Physicochemical trends: Position 46, Side chain branching . . . 4
S27 Physicochemical trends: Position 46, Charge/polarity/aromaticity . . . 5
S28 Physicochemical trends: Position 46, Hydrophobicity . . . 6
S29 Physicochemical trends: Position 48, Accessible surface area . . . 7
S30 Physicochemical trends: Position 48, Side chain branching . . . 8
S31 Physicochemical trends: Position 48, Charge/polarity/aromaticity . . . 9
S32 Physicochemical trends: Position 48, Helical propensity (N′ scale) . . . 10
S33 Physicochemical trends: Position 48, Hydrophobicity . . . 11
S34 Physicochemical trends: Position 50, Accessible surface area . . . 12
S35 Physicochemical trends: Position 50, Side chain branching . . . 13
S36 Physicochemical trends: Position 50, Charge/polarity/aromaticity . . . 14
S37 Physicochemical trends: Position 50, Helical propensity (N1 scale) . . . 15
S38 Physicochemical trends: Position 50, Hydrophobicity . . . 16
S39 Physicochemical trends: Position 51, Accessible surface area . . . 17
S40 Physicochemical trends: Position 51, Side chain branching . . . 18
S41 Physicochemical trends: Position 51, Charge/polarity/aromaticity . . . 19
S42 Physicochemical trends: Position 51, Helical propensity (N2 scale) . . . 20
S43 Physicochemical trends: Position 51, Hydrophobicity . . . 21
S44 Physicochemical trends: Position 52, Accessible surface area . . . 22
S45 Physicochemical trends: Position 52, Side chain branching . . . 23
S46 Physicochemical trends: Position 52, Charge/polarity/aromaticity . . . 24
S47 Physicochemical trends: Position 52, Helical propensity (N3 scale) . . . 25
S48 Physicochemical trends: Position 52, Hydrophobicity . . . 26
S49 Physicochemical trends: Position 54, Accessible surface area . . . 27
S50 Physicochemical trends: Position 54, Side chain branching . . . 28
S51 Physicochemical trends: Position 54, Charge/polarity/aromaticity . . . 29
S52 Physicochemical trends: Position 54, Helical propensity (C4 scale) . . . 30
S53 Physicochemical trends: Position 54, Helical propensity (Midpoint scale) . . . 31
S54 Physicochemical trends: Position 54, Hydrophobicity . . . 32

Figures S25-S54. Physico-chemical trends: positions 46-54. Repression assay data for each position were color-coded according to various physico-chemical scales. For example, Figures S25-S28 show results for position 46 color-coded by accessible surface area of the free side chain [46], side chain branching, charge/polarity/aromaticity, and hydrophobicity [45]. The relevant parameter can be determined from the legend in the lower right hand corner of each graph. Positions 50-58 have potential to participate in an alpha helix, and repression assay results were also compared to both average and position-specific helical propensities [47]. For simplicity, only one helix color scale is used as a legend, with magenta corresponding to high propensity and green corresponding to low propensity. Since wild-type LacI and PurR have different length helices [20], we compared multiple helical scales to results for each position. However, no scale showed good correlation with the functional assay among all chimeras.

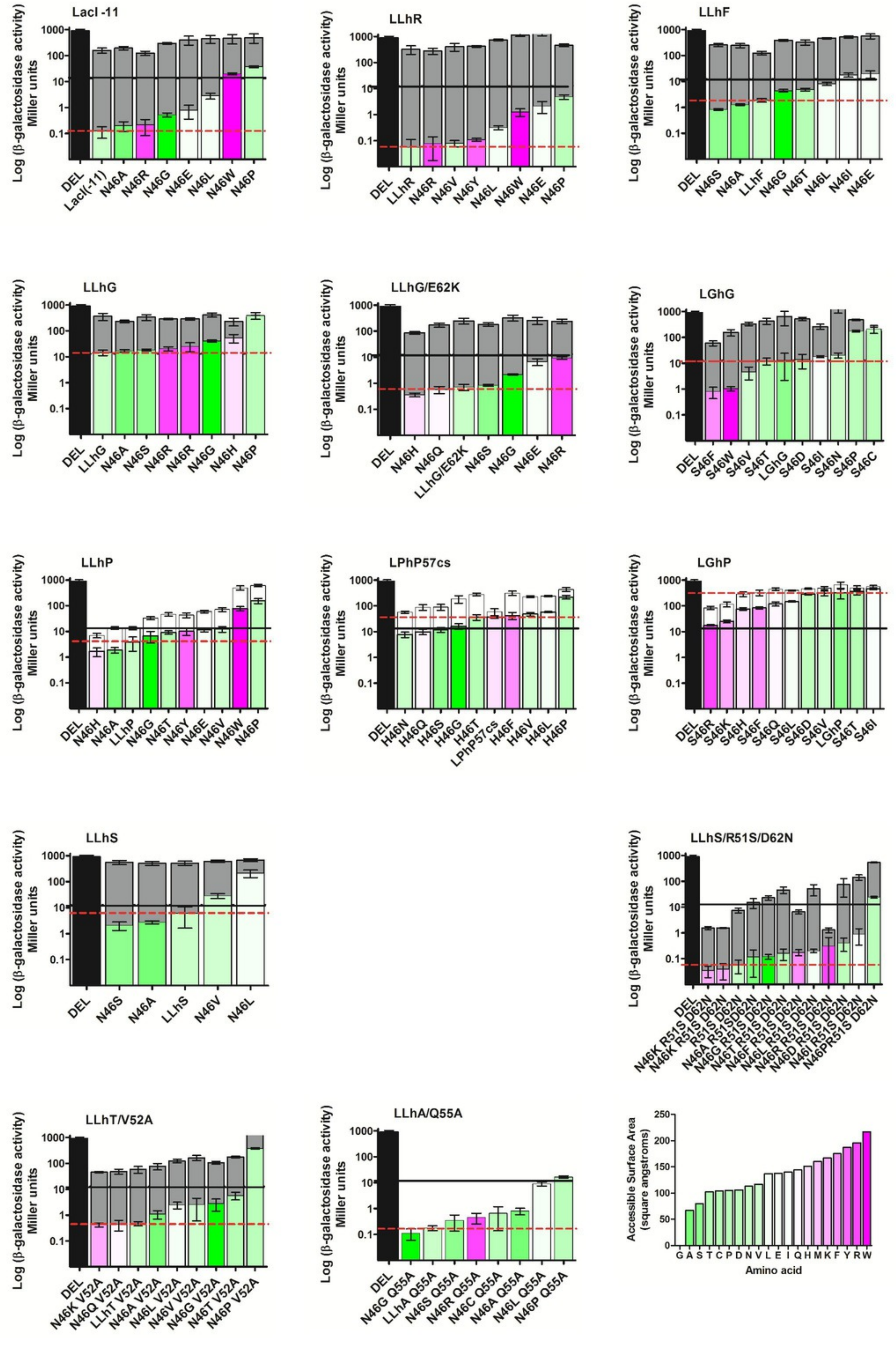

Figure S25: Physicochemical trends: Position 46, Accessible surface area

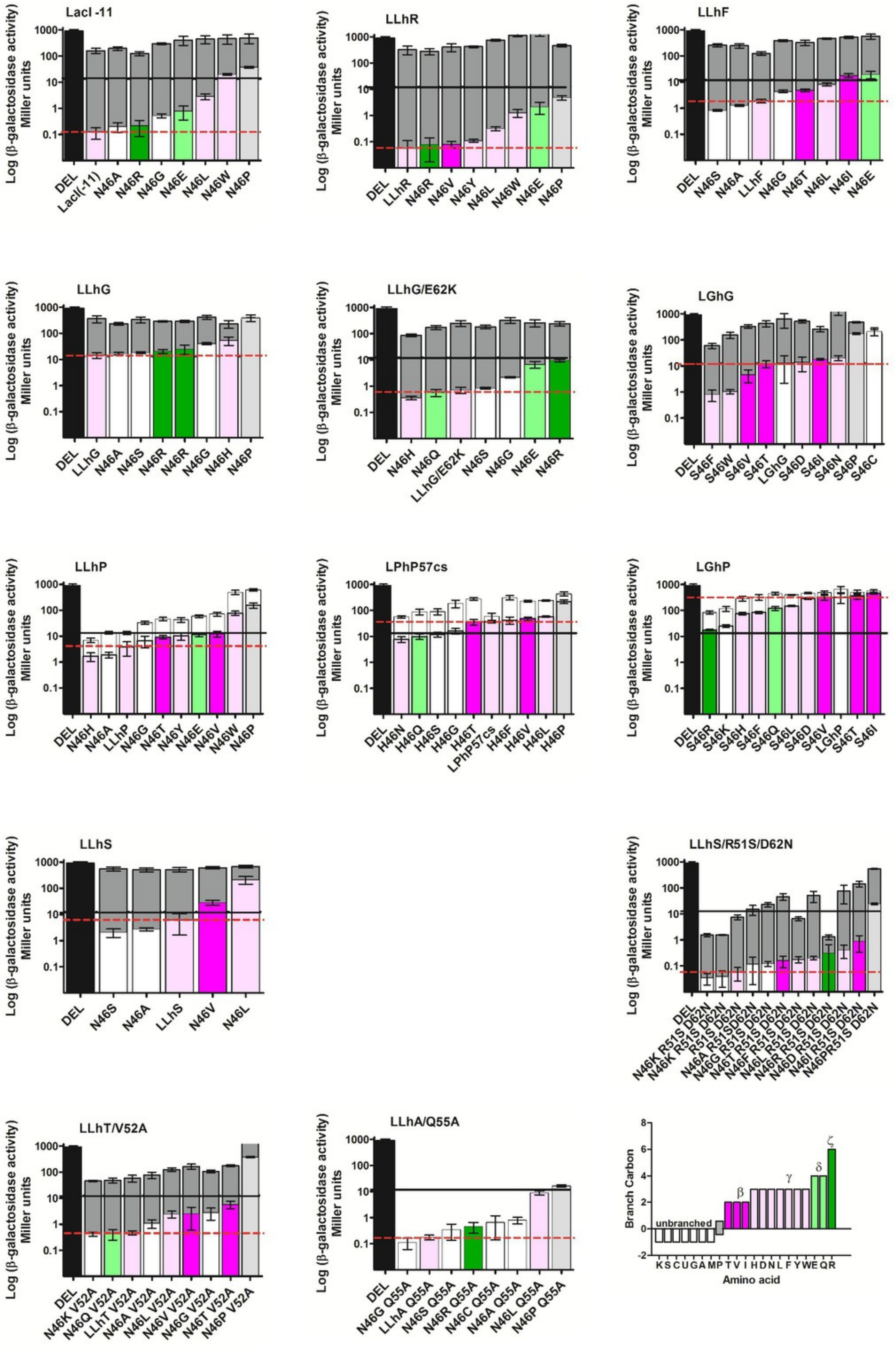

Figure S26: Physicochemical trends: Position 46, Side chain branching

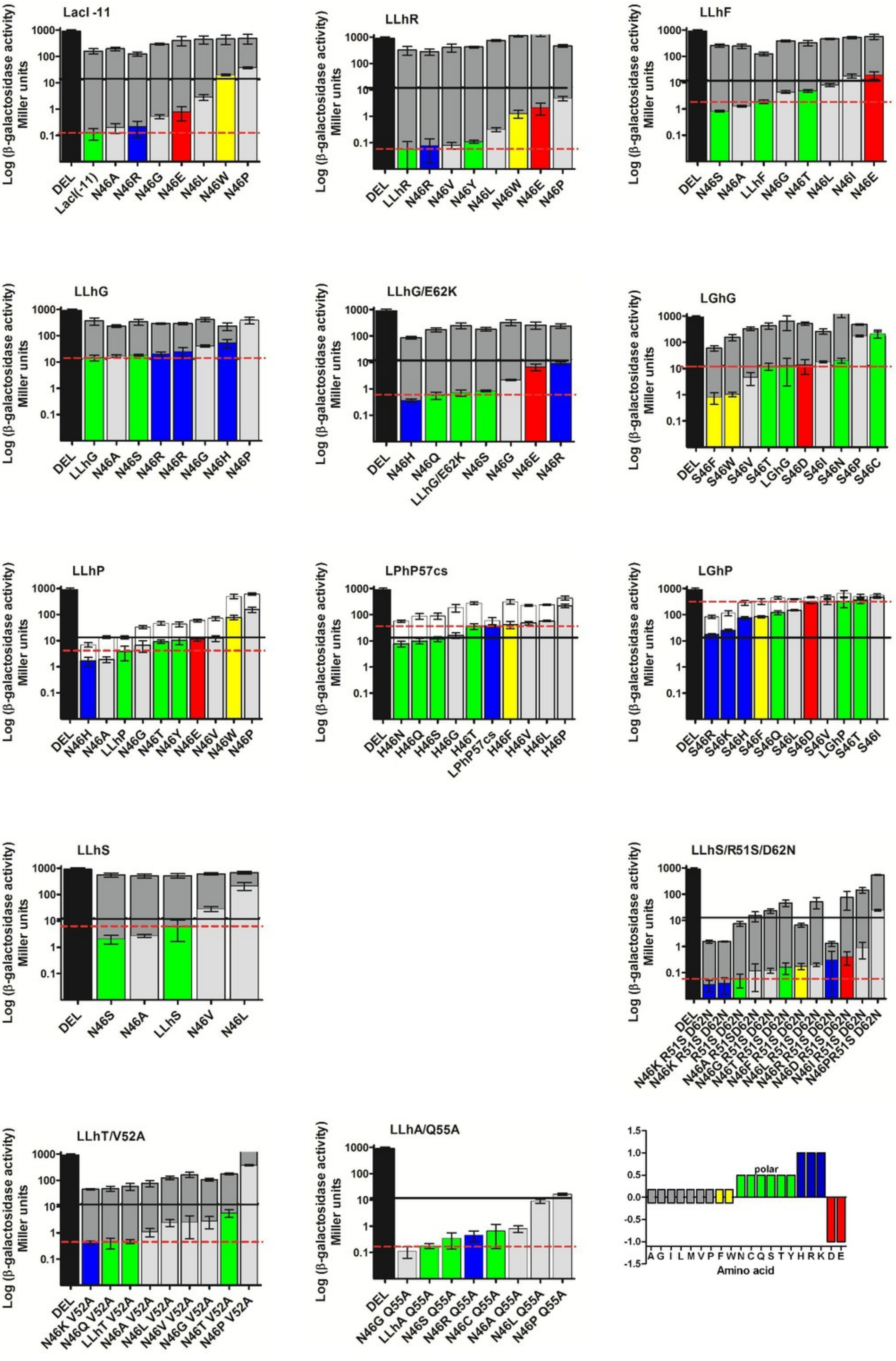

Figure S27: Physicochemical trends: Position 46, Charge/polarity/aromaticity

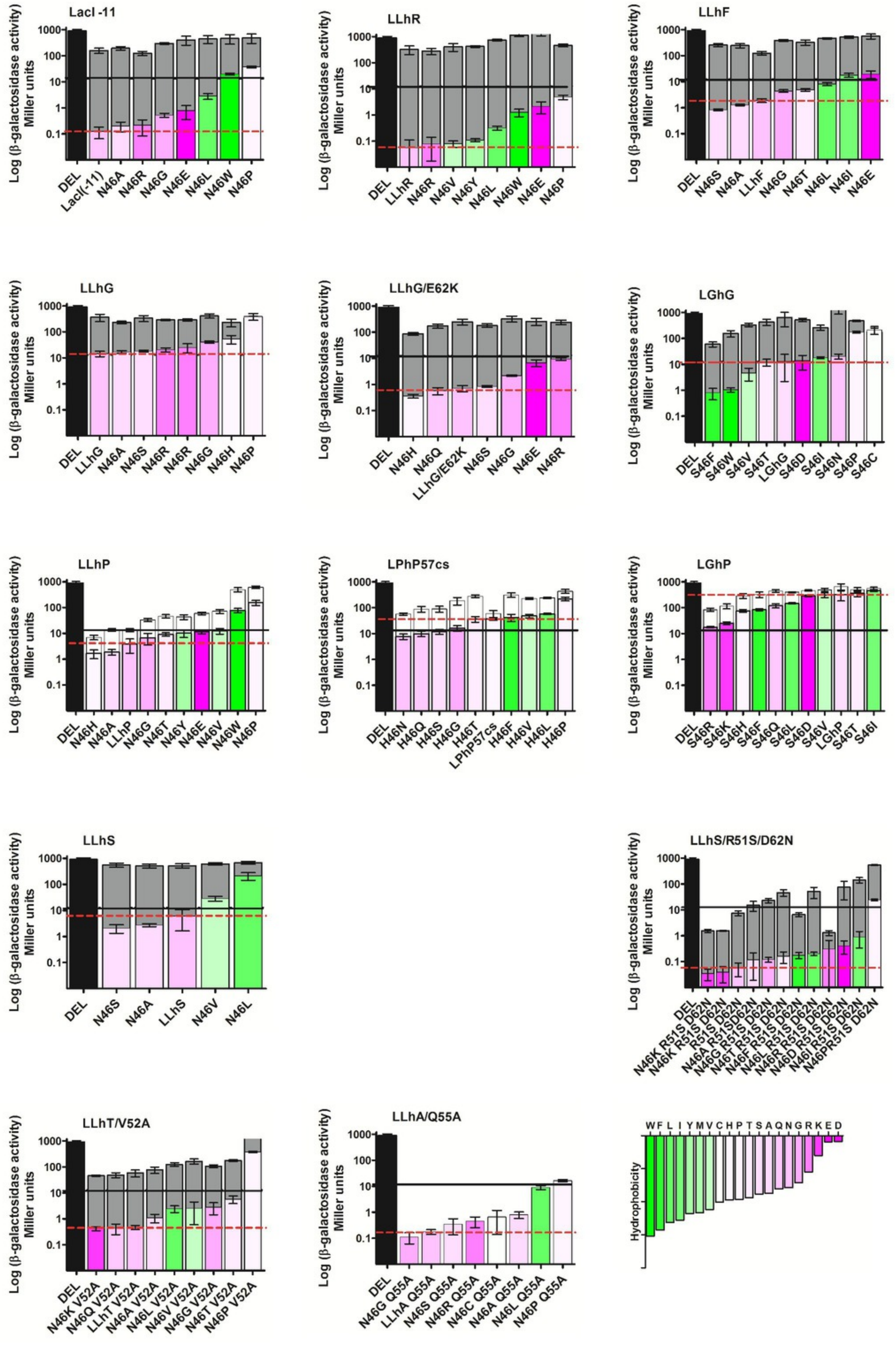

Figure S28: Physicochemical trends: Position 46, Hydrophobicity

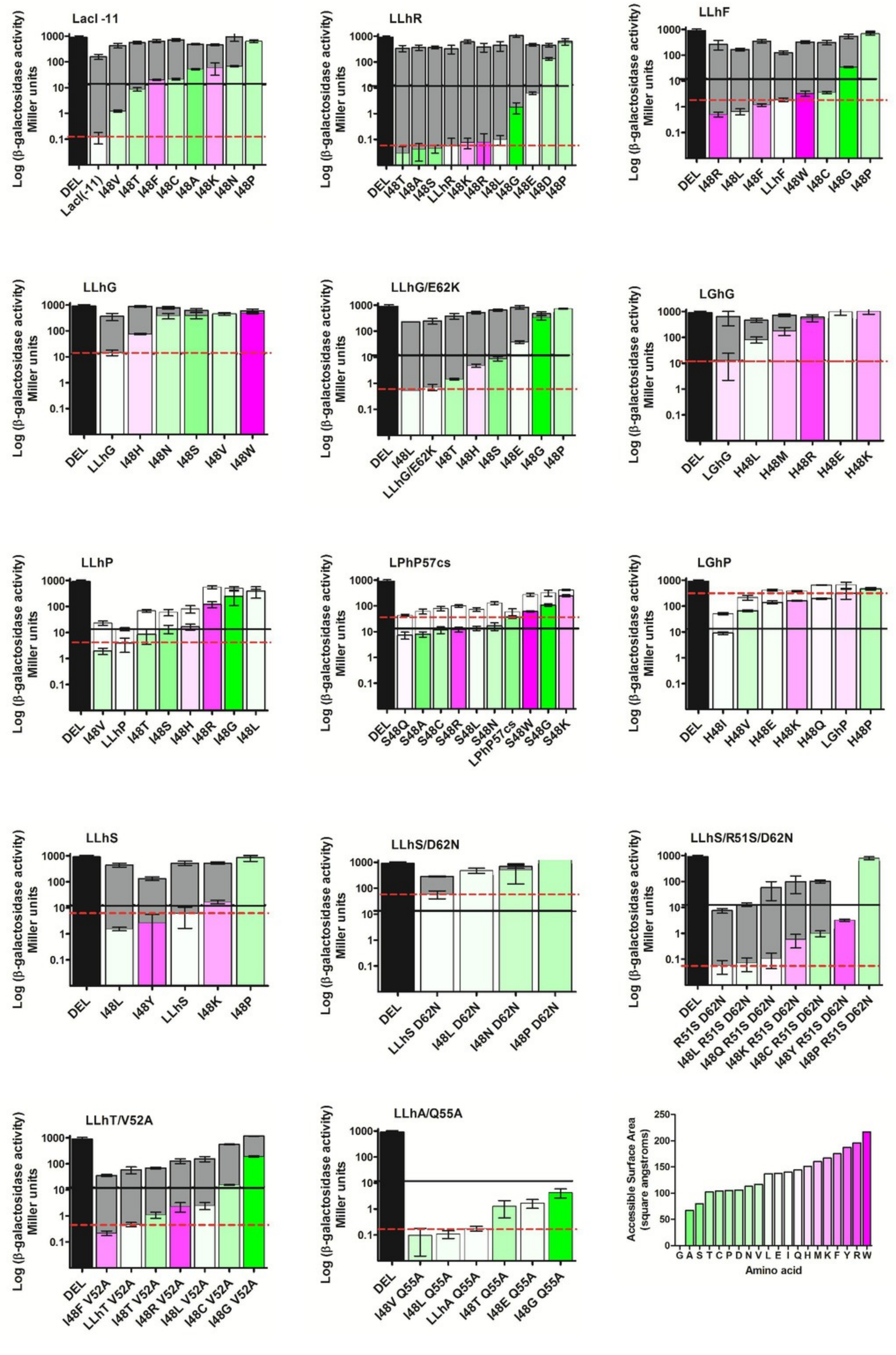

Figure S29: Physicochemical trends: Position 48, Accessible surface area

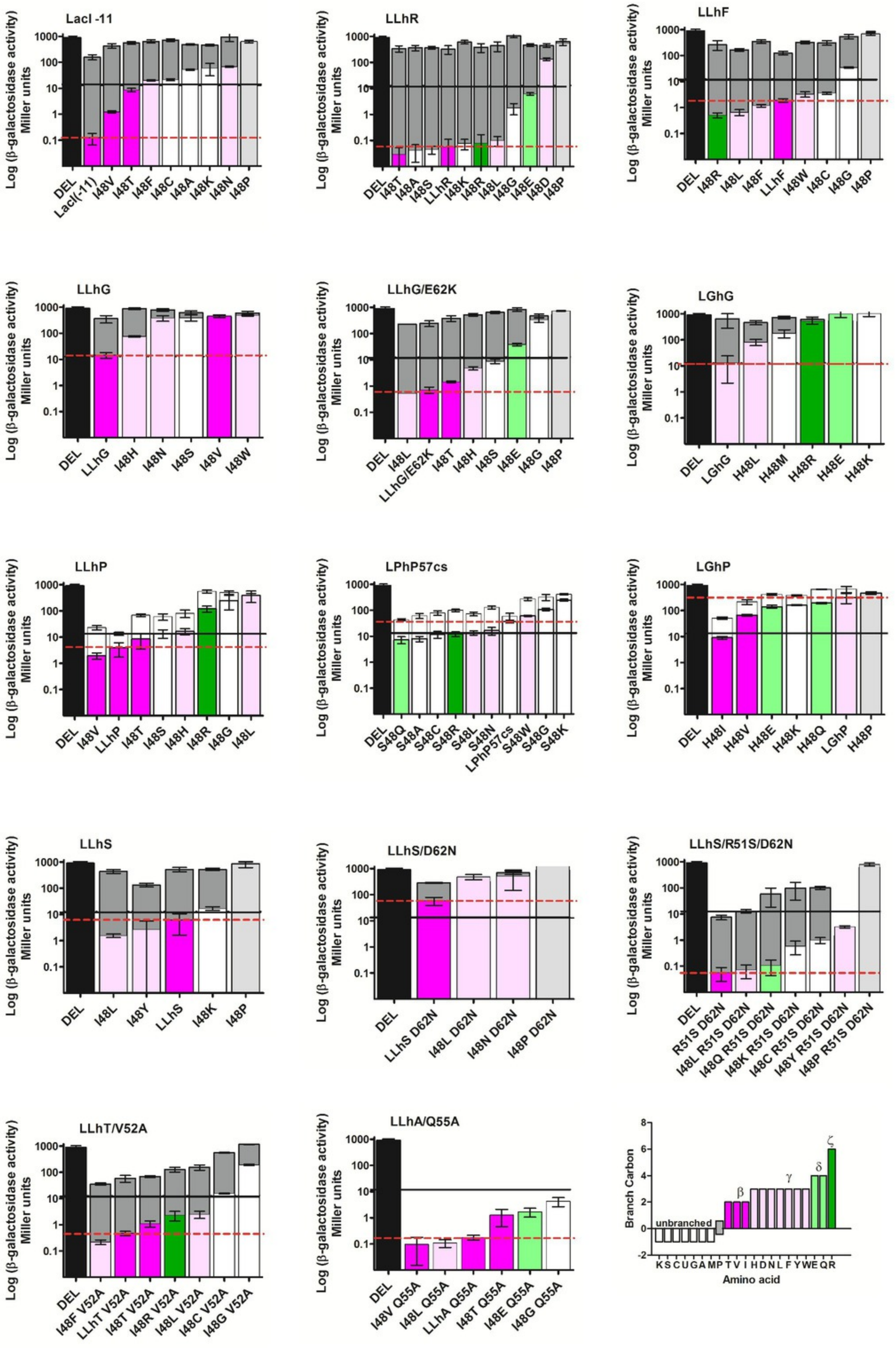

Figure S30: Physicochemical trends: Position 48, Side chain branching

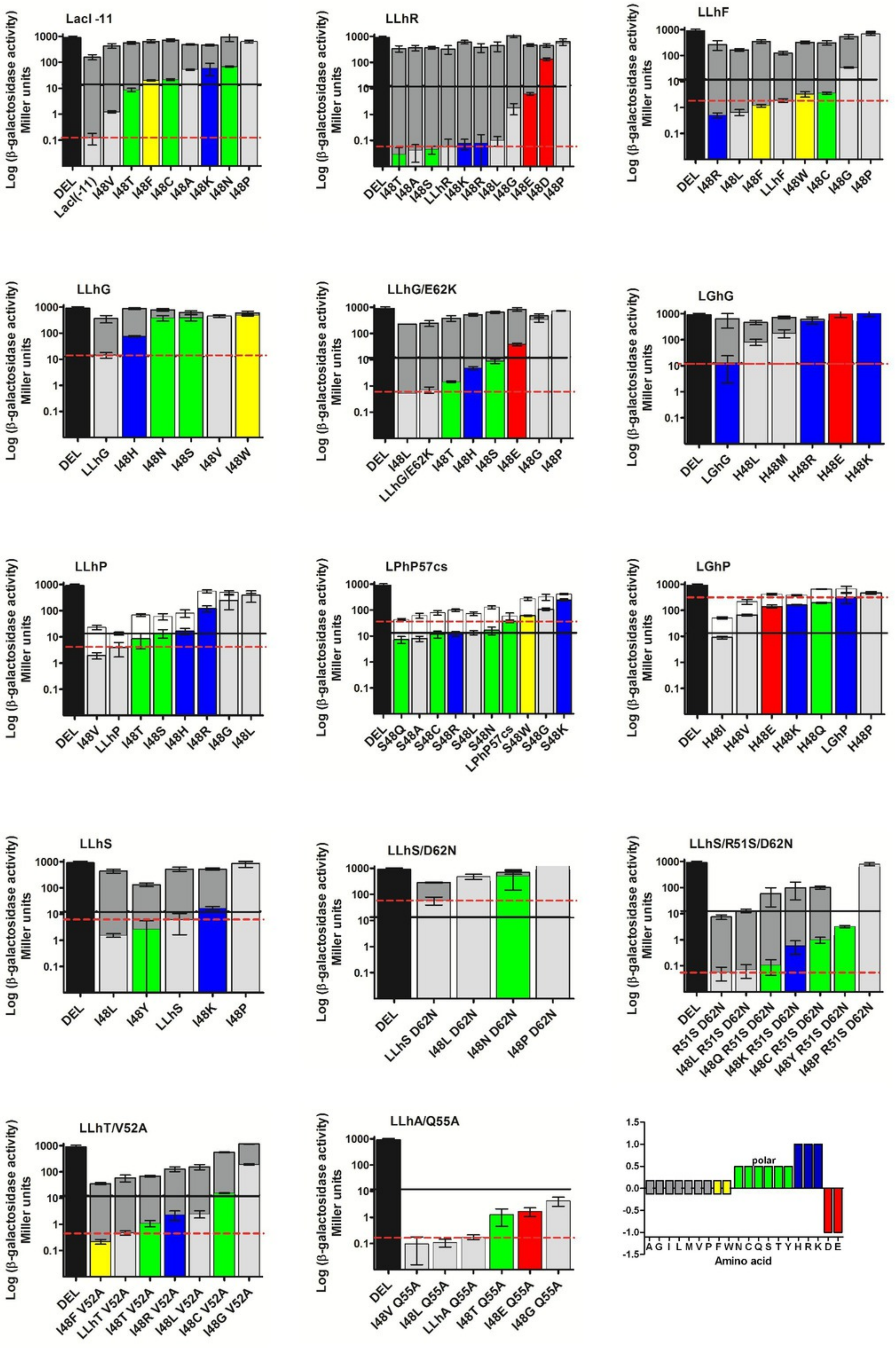

Figure S31: Physicochemical trends: Position 48, Charge/polarity/aromaticity

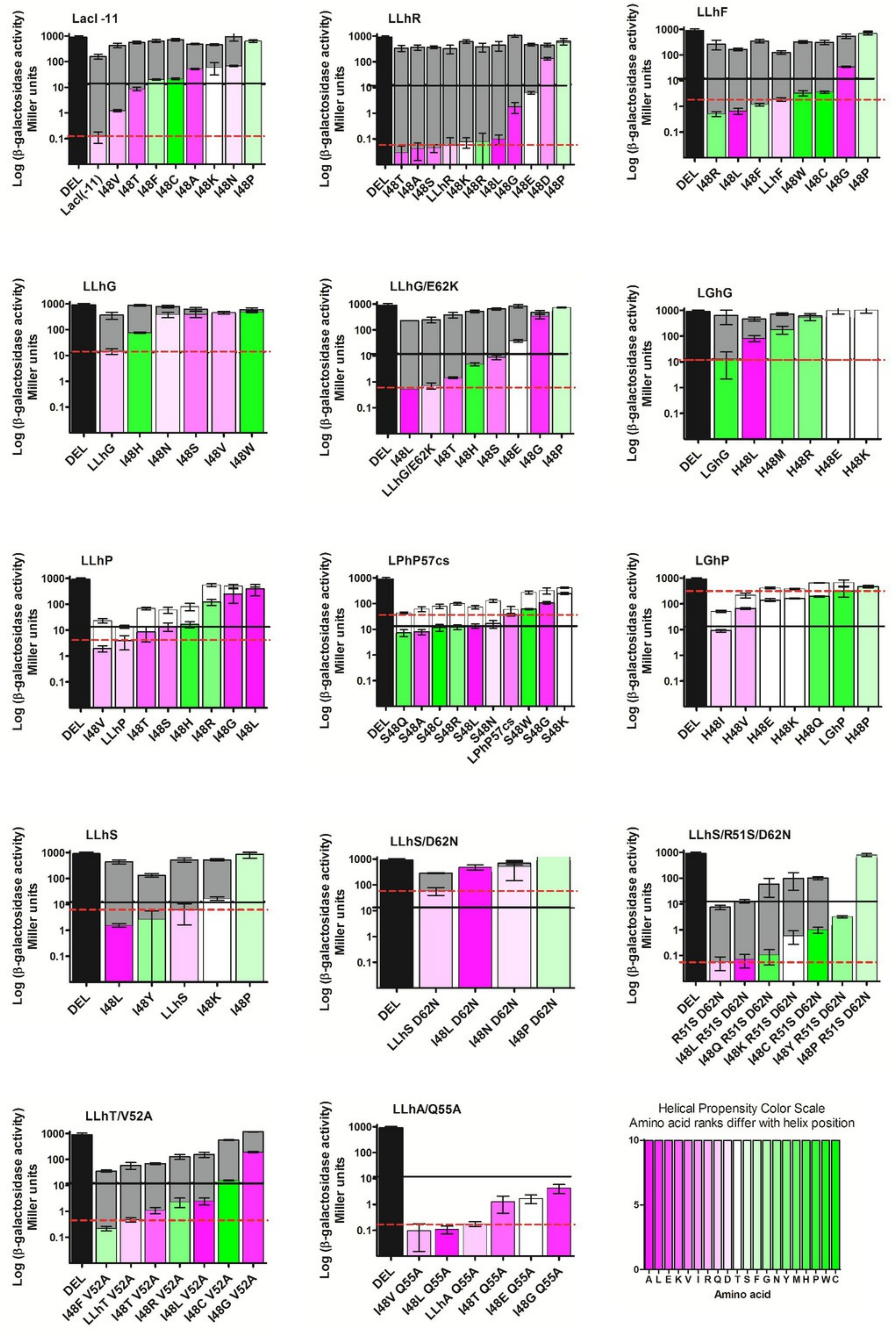

Figure S32: Physicochemical trends: Position 48, Helical propensity (N′ scale)

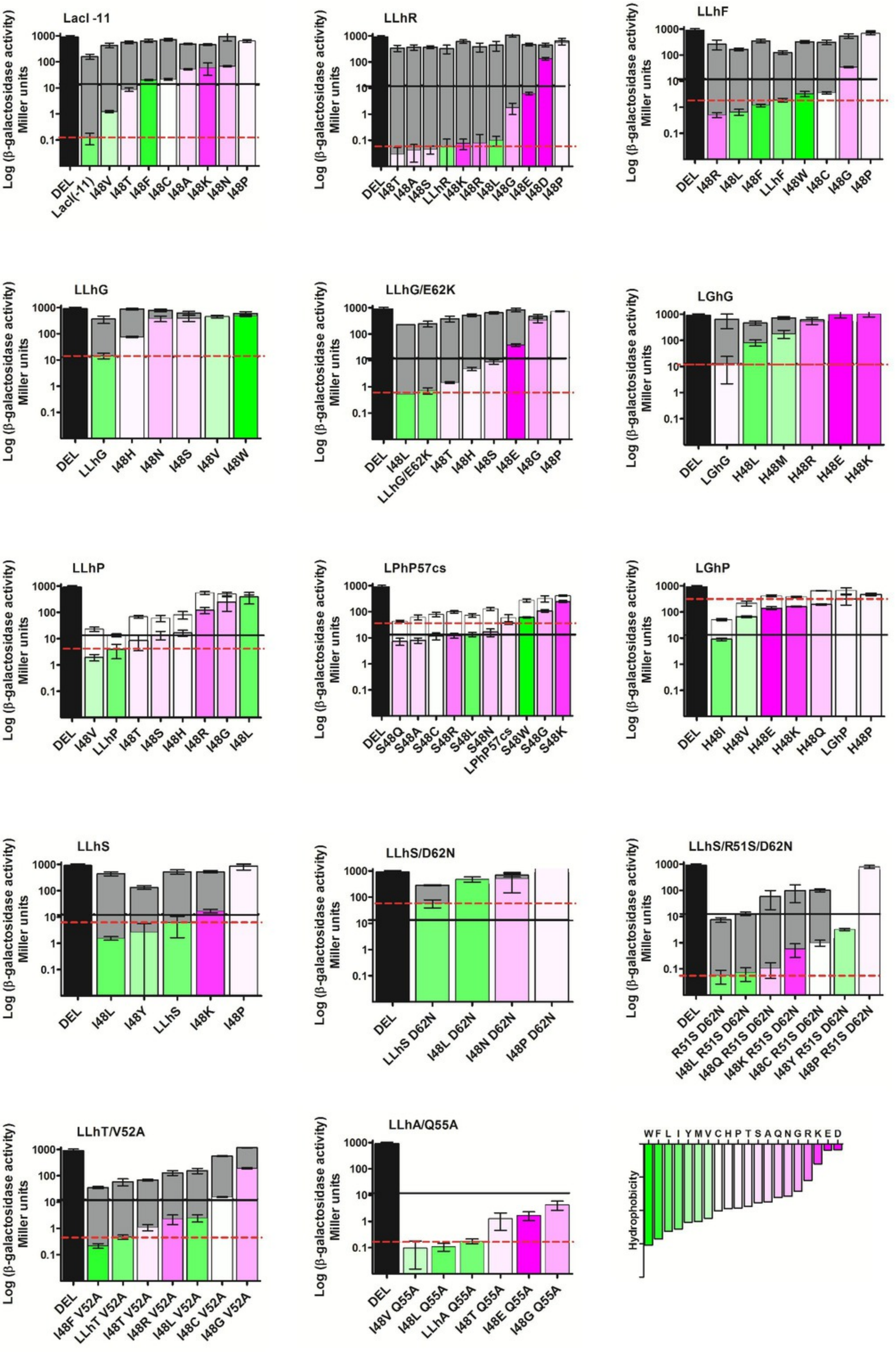

Figure S33: Physicochemical trends: Position 48, Hydrophobicity

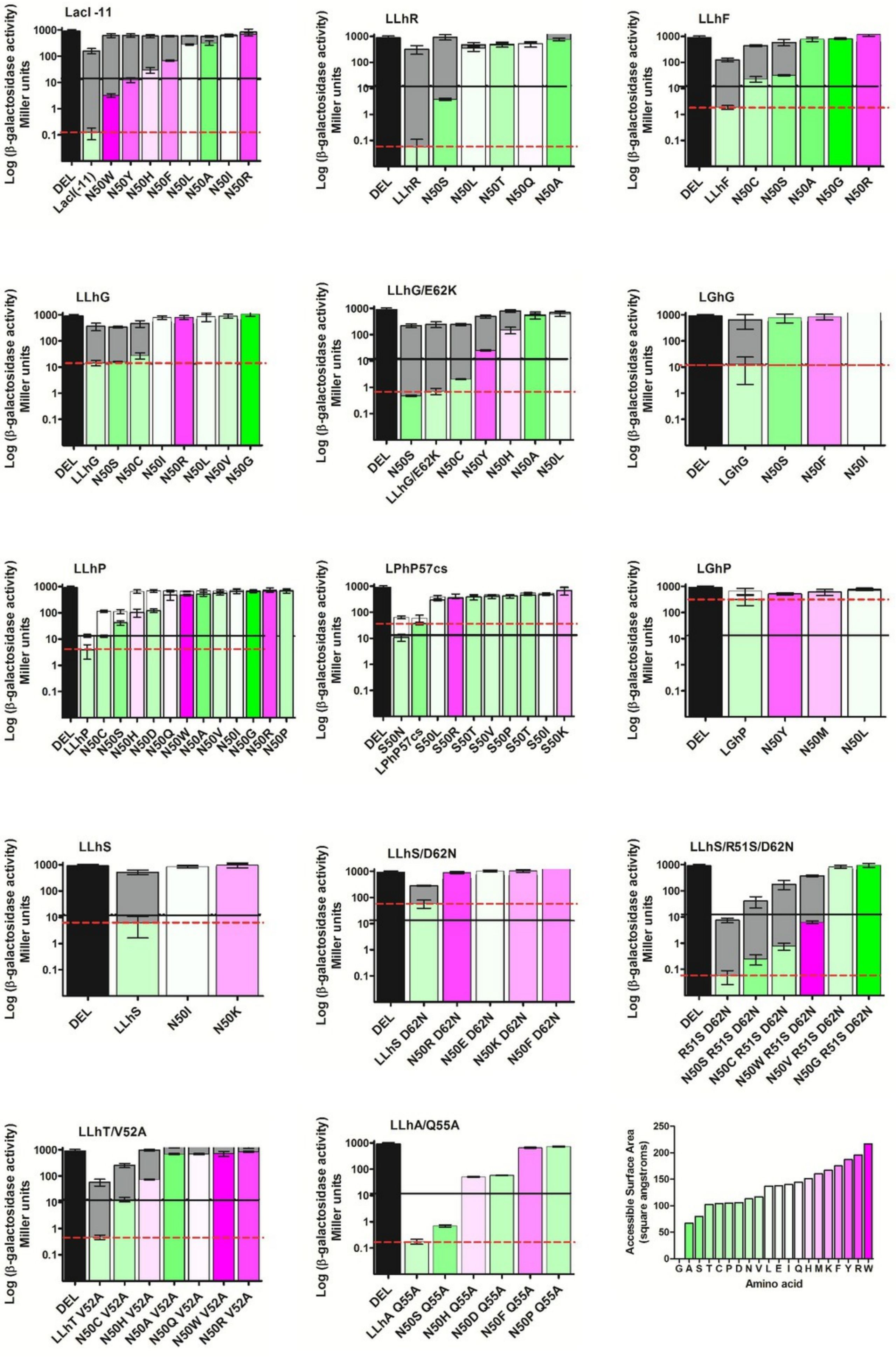

Figure S34: Physicochemical trends: Position 50, Accessible surface area

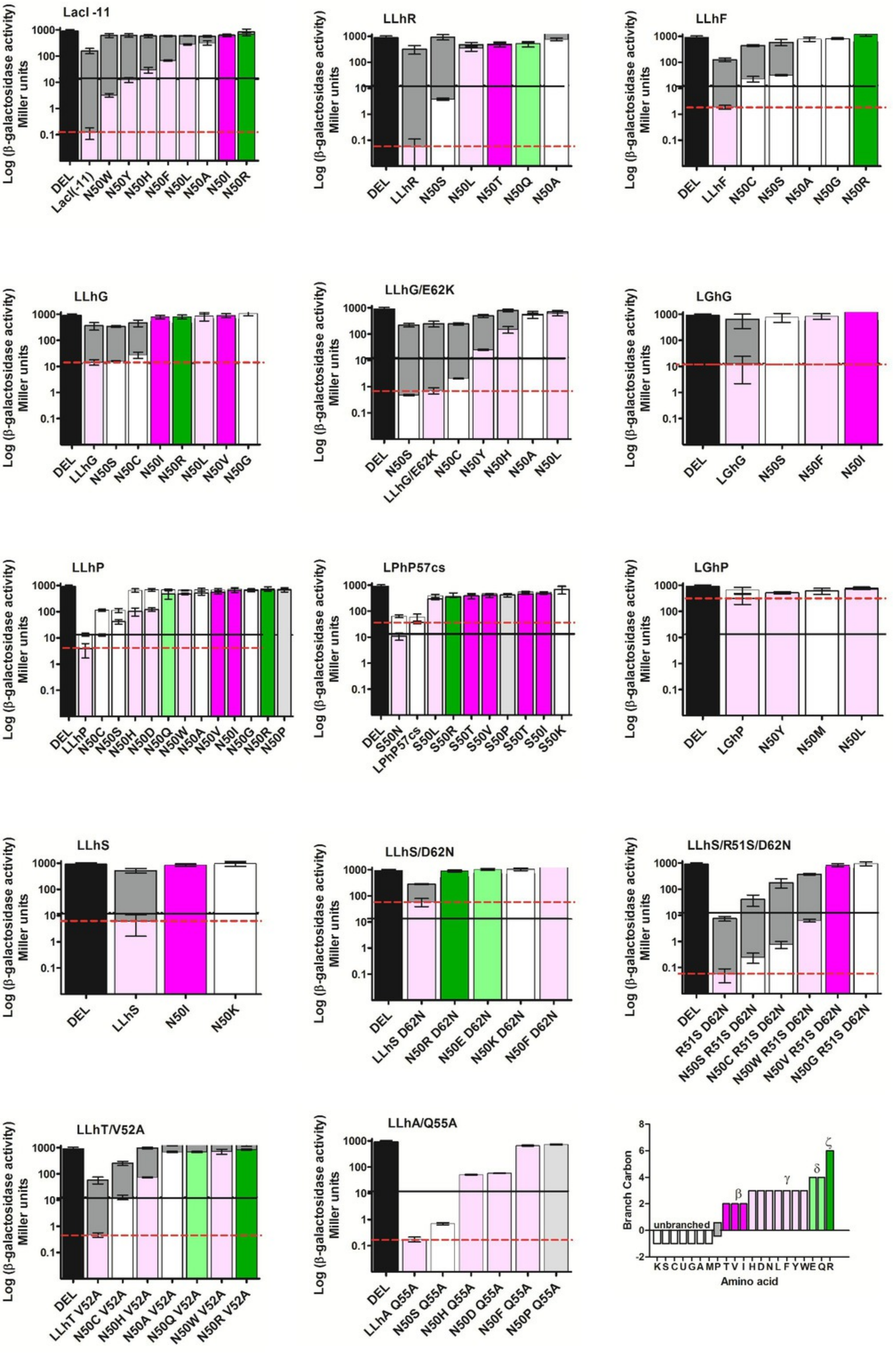

Figure S35: Physicochemical trends: Position 50, Side chain branching

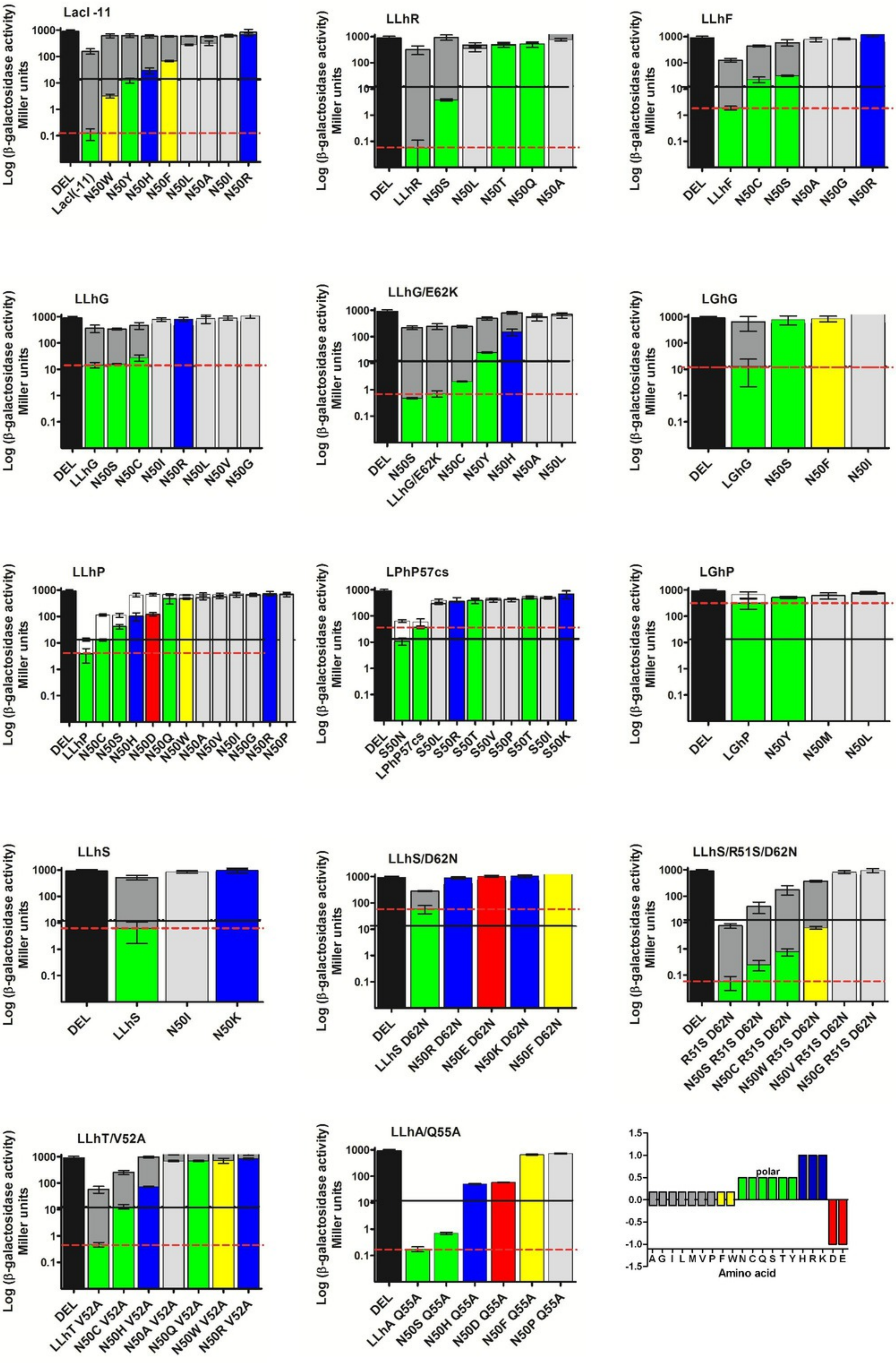

Figure S36: Physicochemical trends: Position 50, Charge/polarity/aromaticity

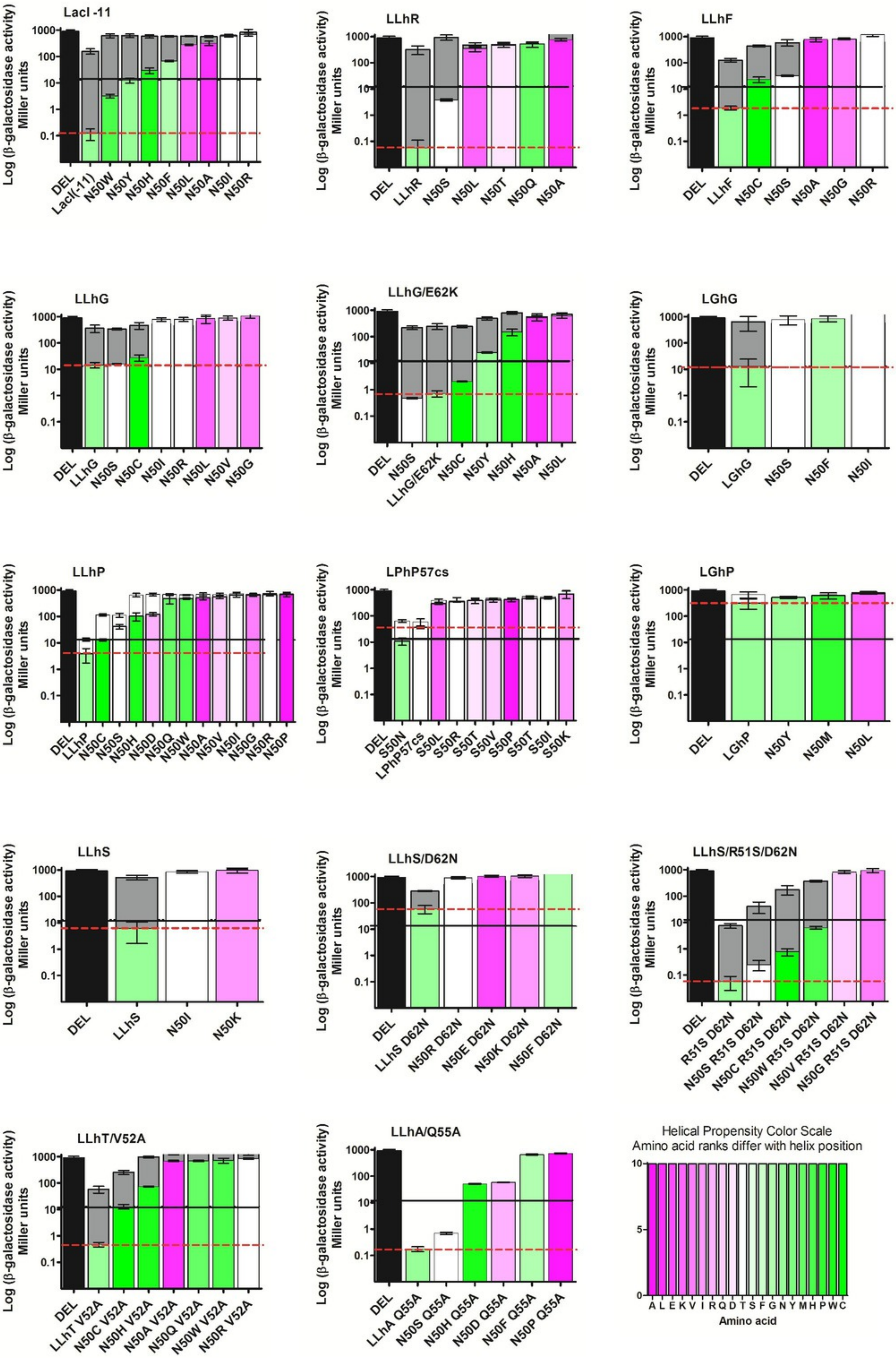

Figure S37: Physicochemical trends: Position 50, Helical propensity (N1 scale)

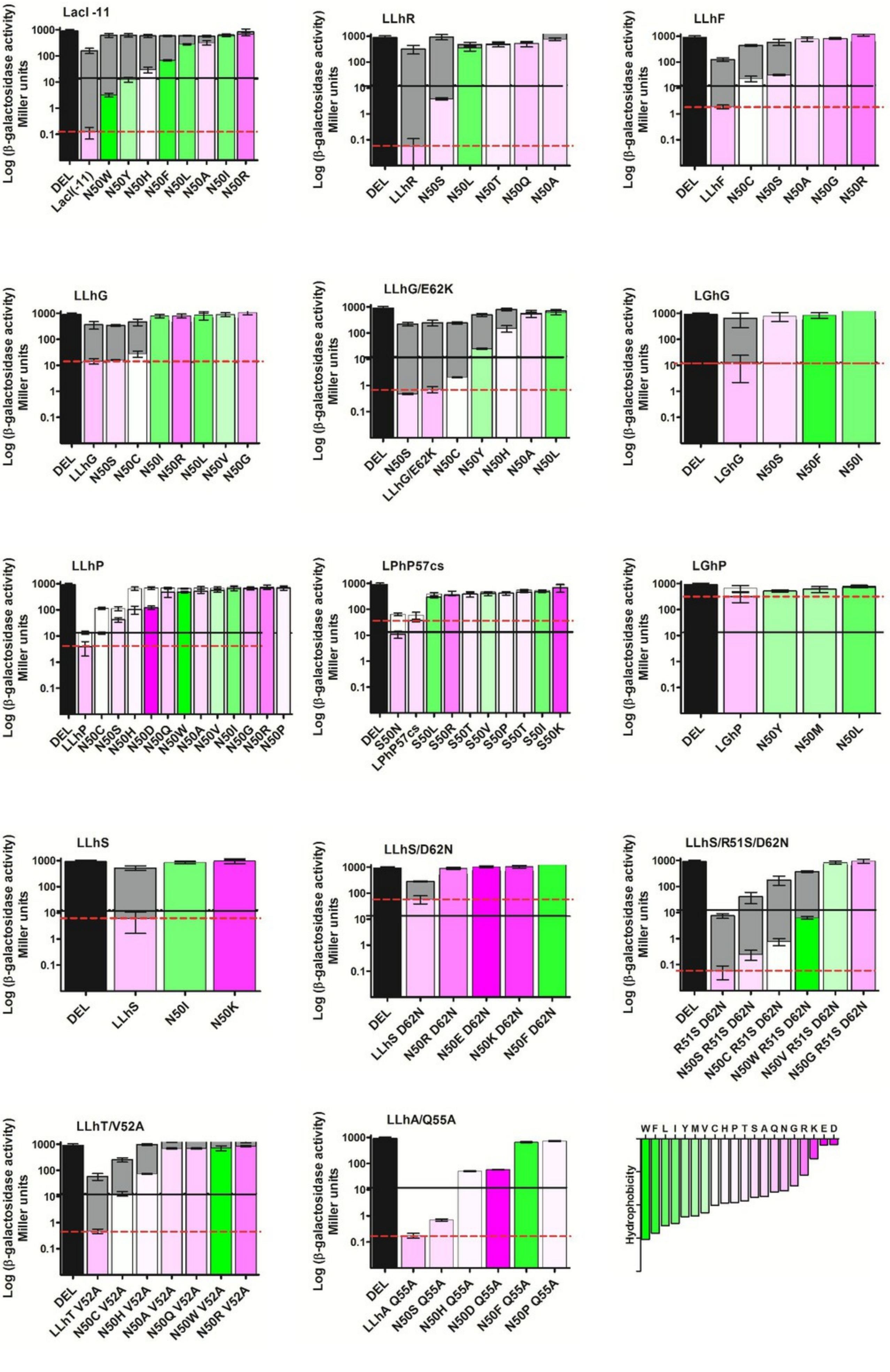

Figure S38: Physicochemical trends: Position 50, Hydrophobicity

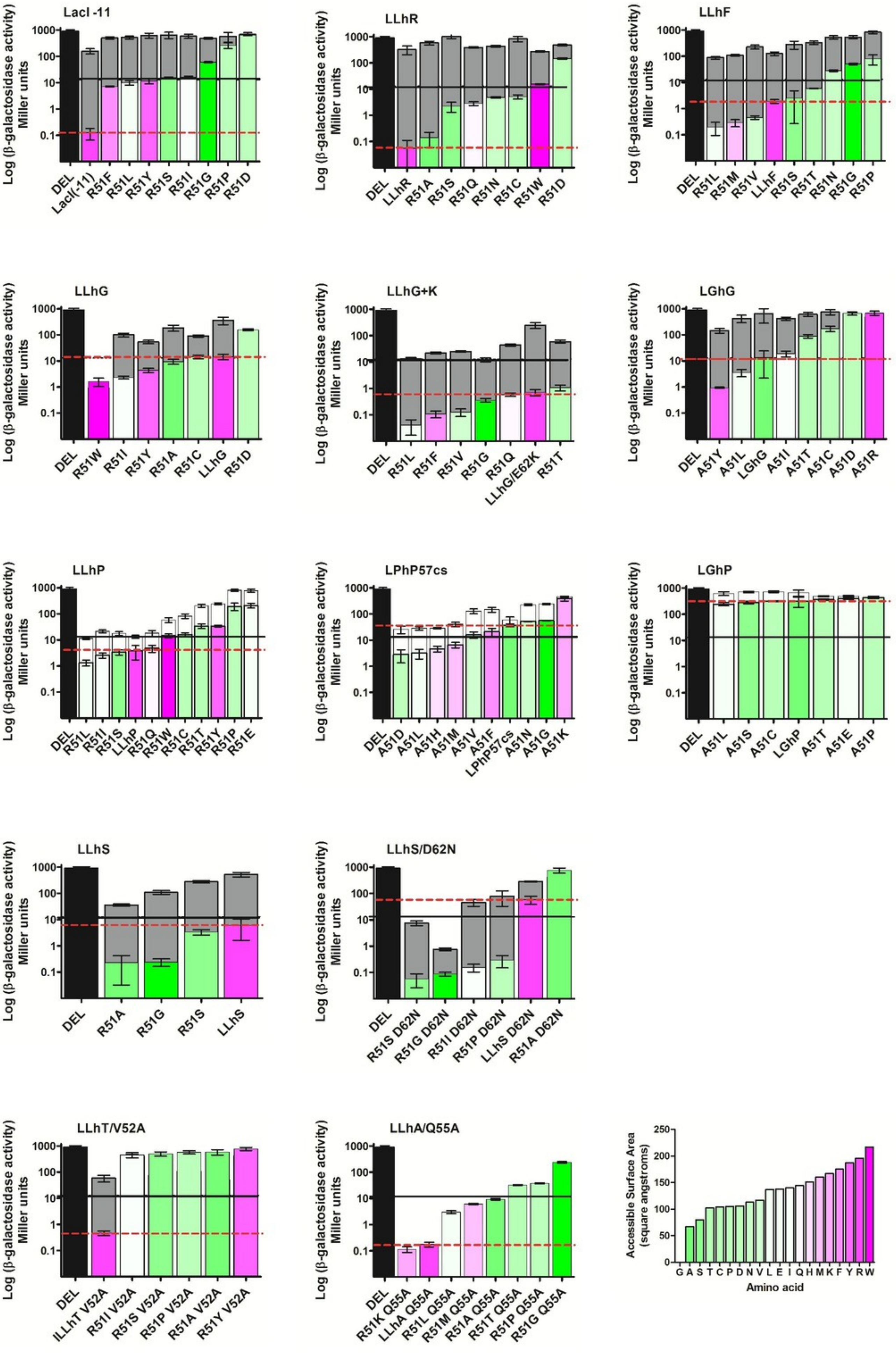

Figure S39: Physicochemical trends: Position 51, Accessible surface area

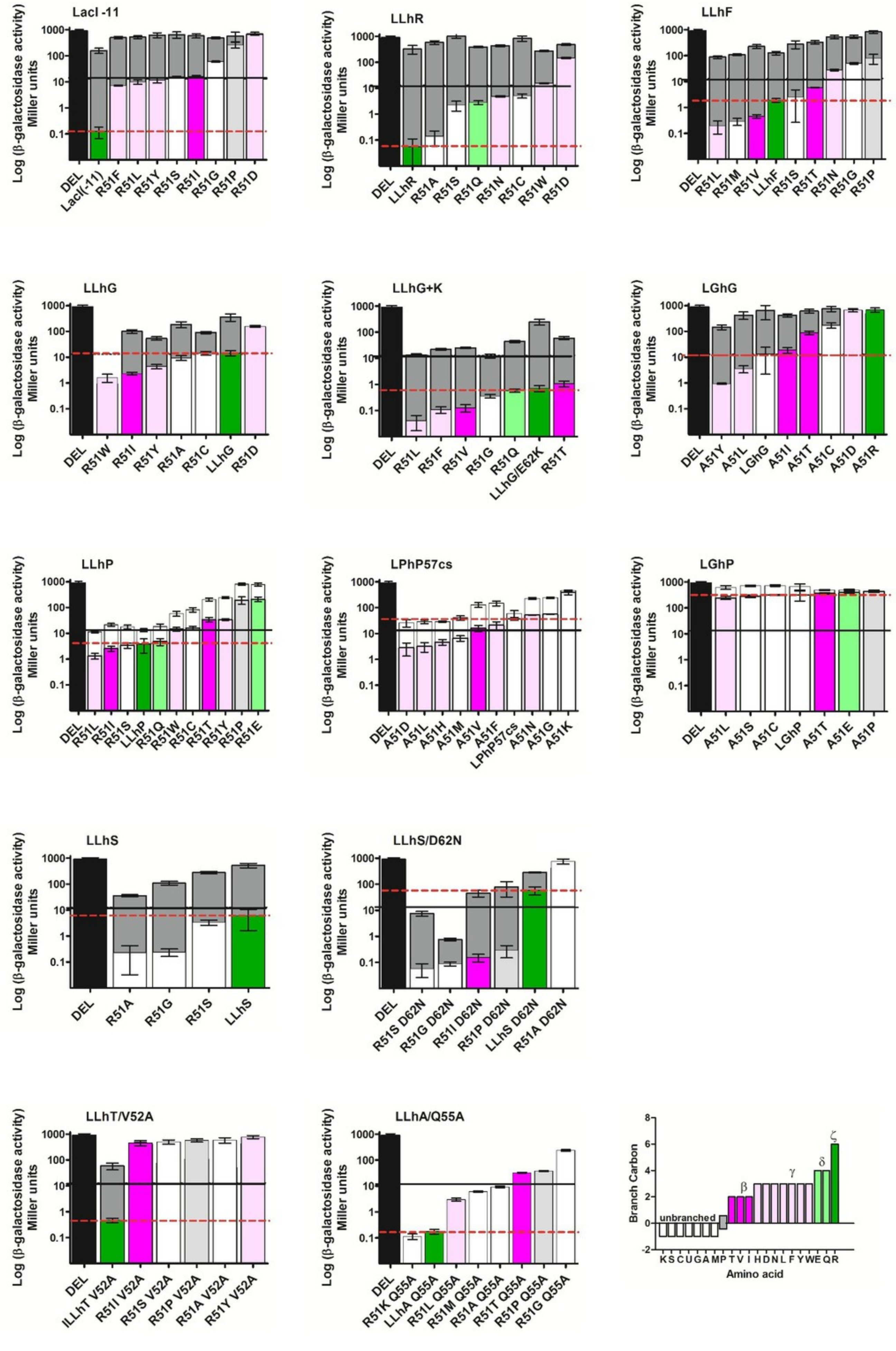

Figure S40: Physicochemical trends: Position 51, Side chain branching

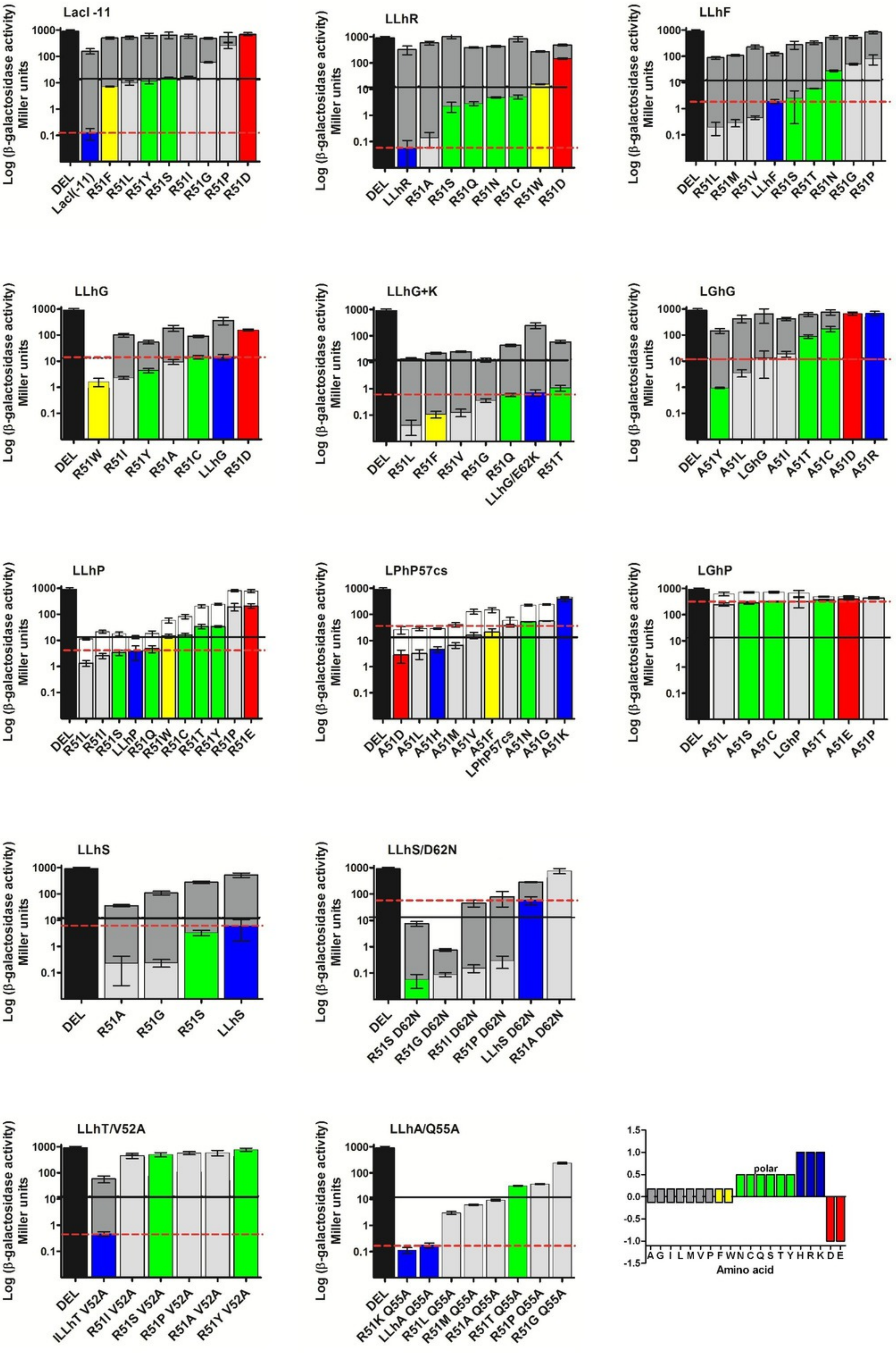

Figure S41: Physicochemical trends: Position 51, Charge/polarity/aromaticity

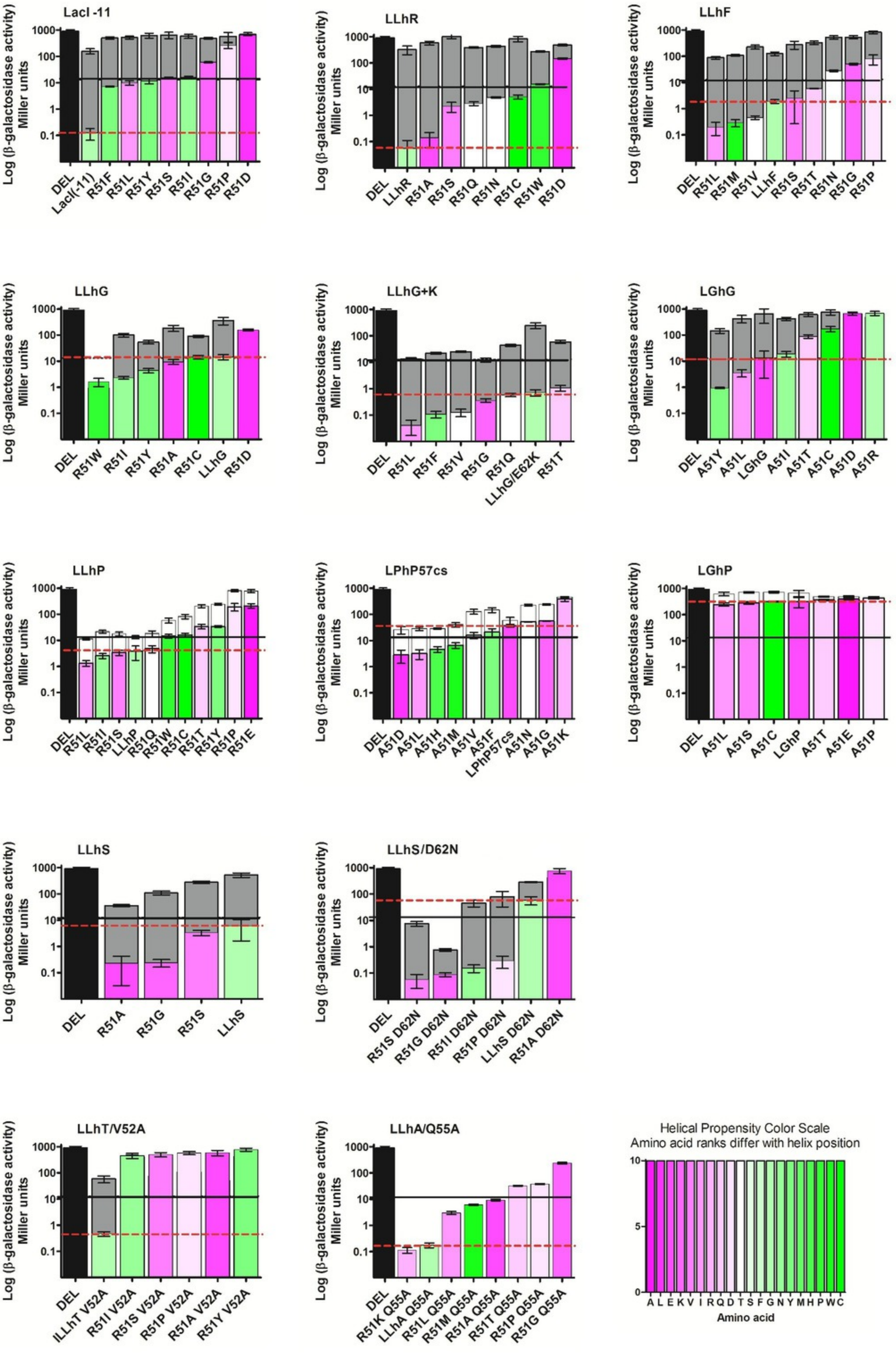

Figure S42: Physicochemical trends: Position 51, Helical propensity (N2 scale)

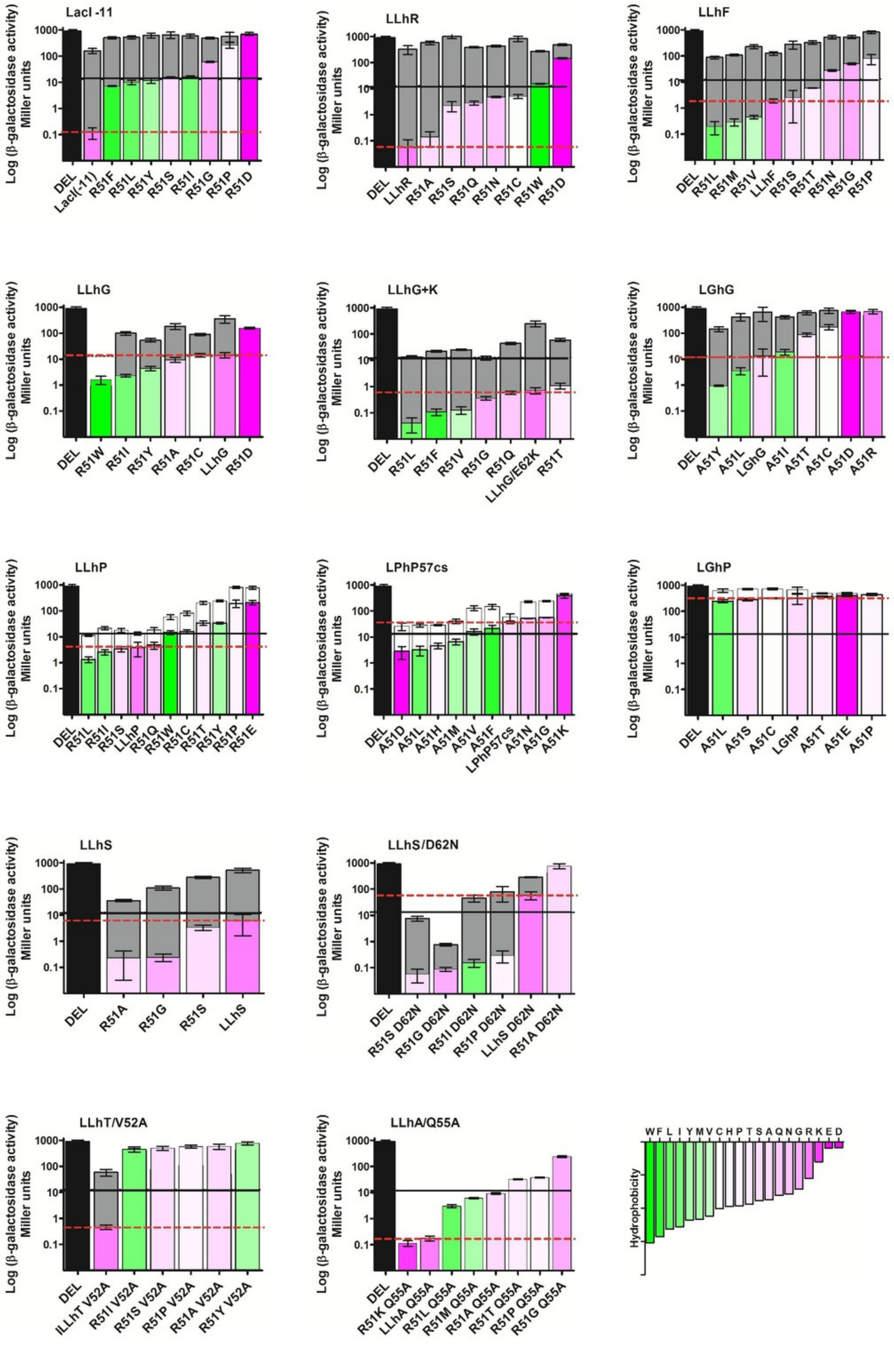

Figure S43: Physicochemical trends: Position 51, Hydrophobicity

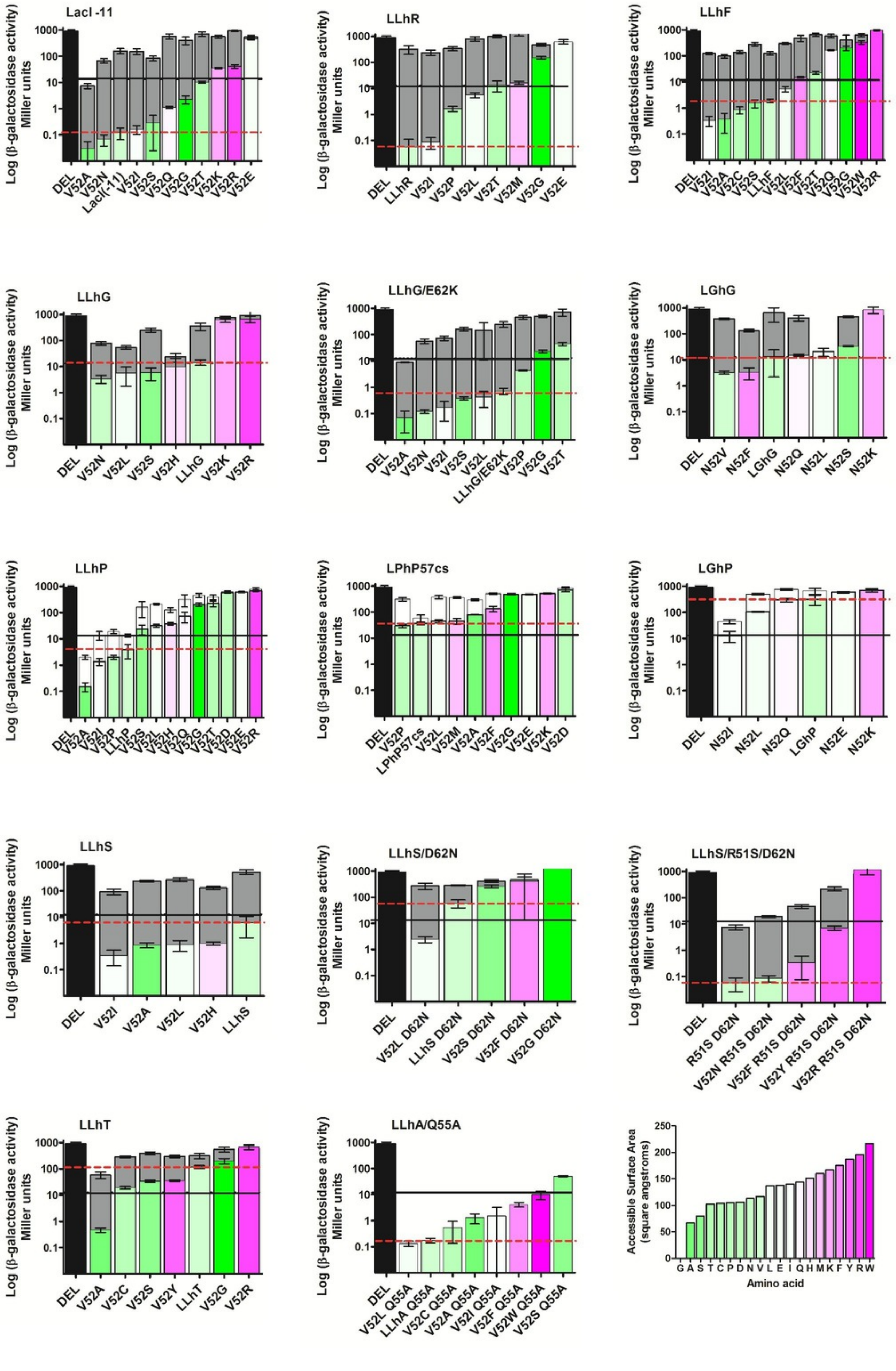

Figure S44: Physicochemical trends: Position 52, Accessible surface area

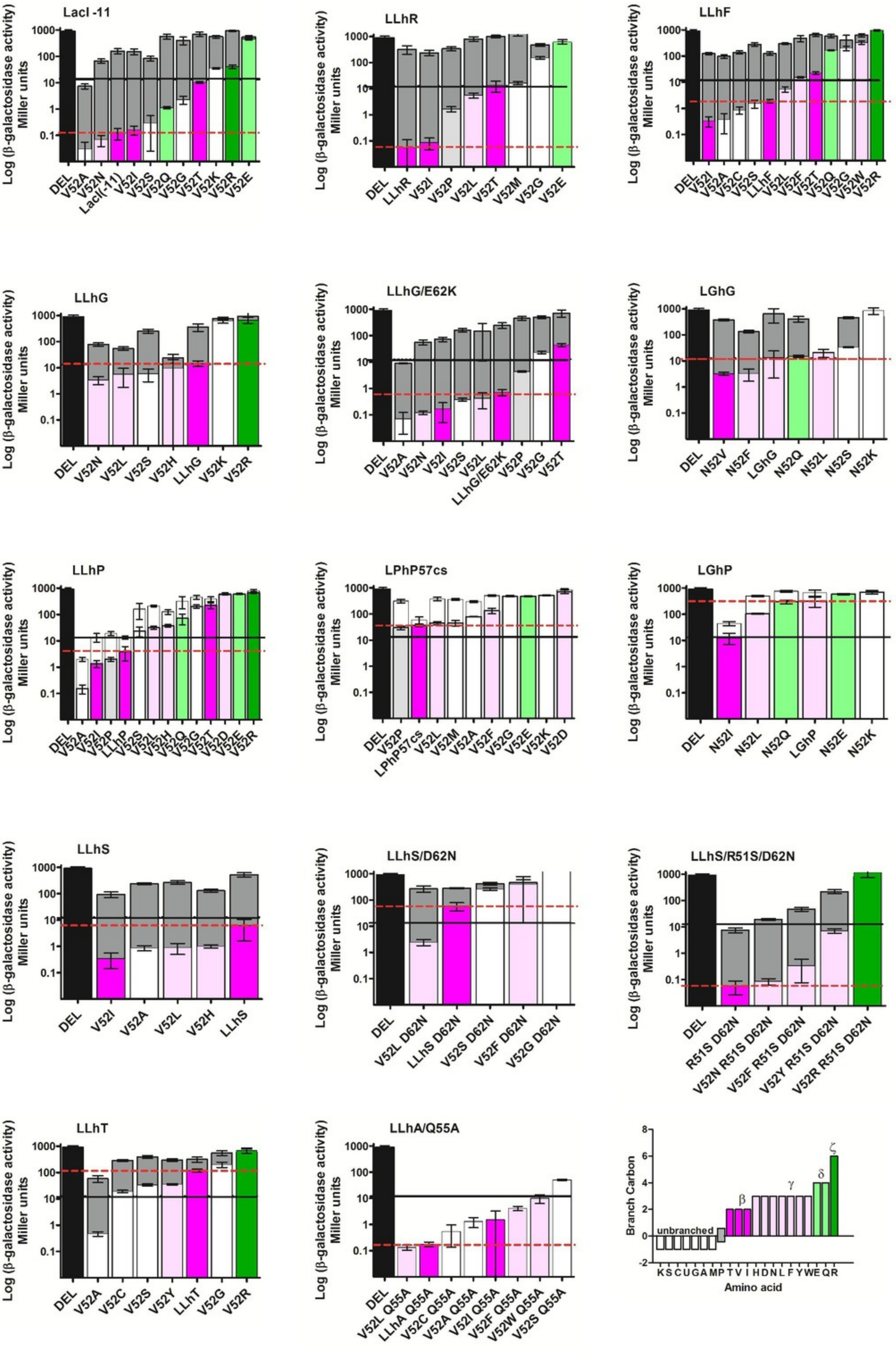

Figure S45: Physicochemical trends: Position 52, Side chain branching

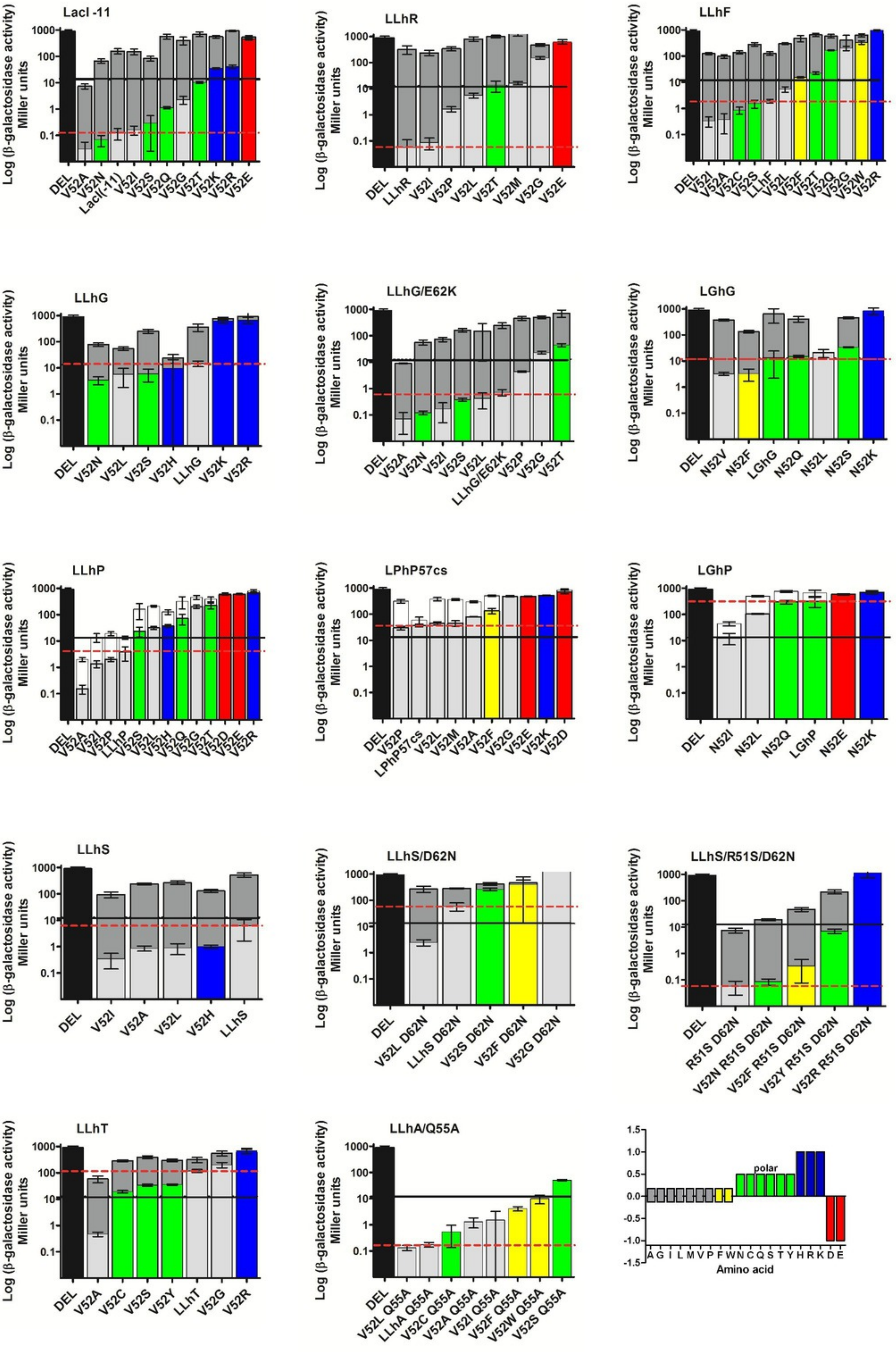

Figure S46: Physicochemical trends: Position 52, Charge/polarity/aromaticity

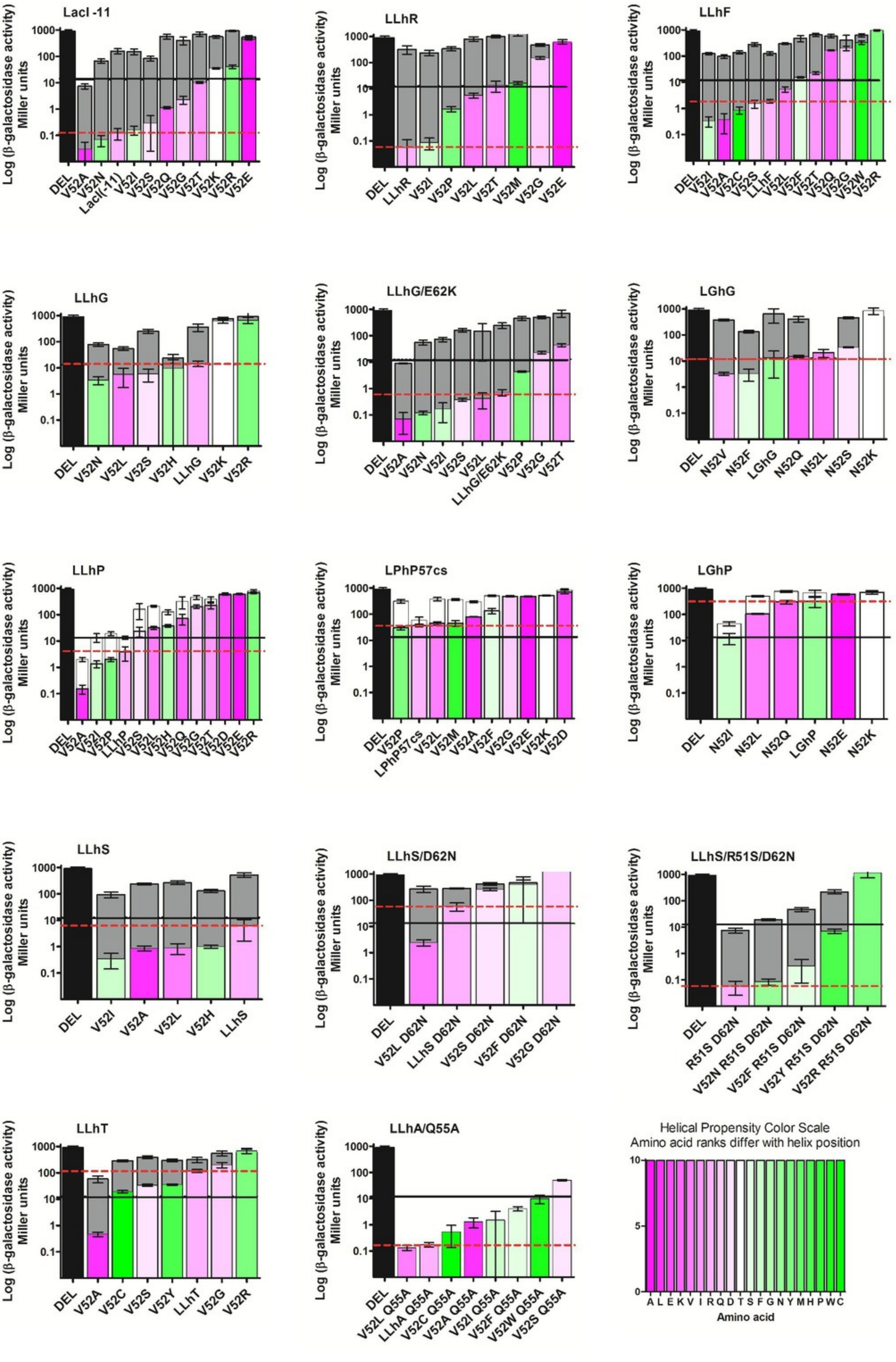

Figure S47: Physicochemical trends: Position 52, Helical propensity (N3 scale)

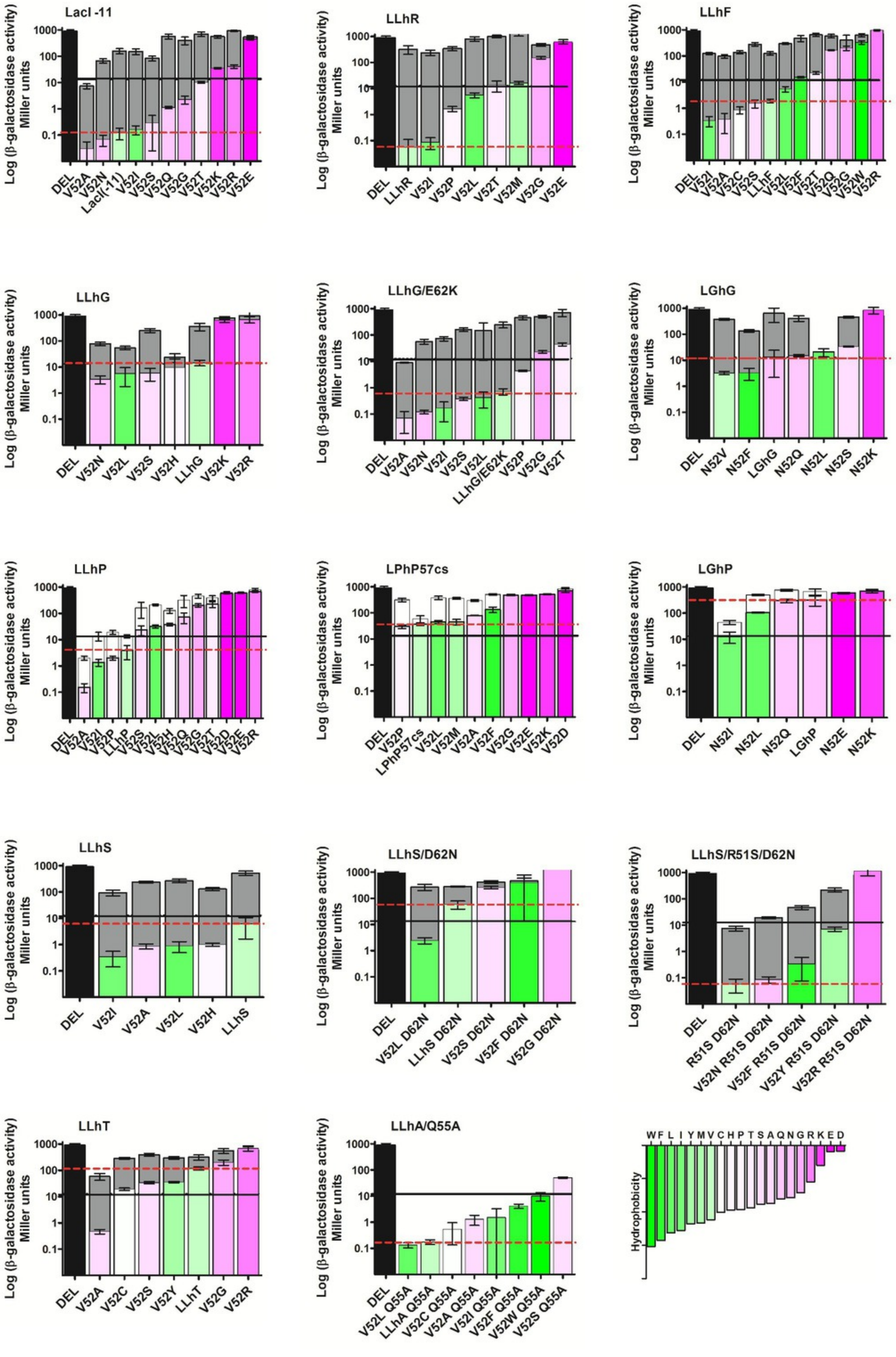

Figure S48: Physicochemical trends: Position 52, Hydrophobicity

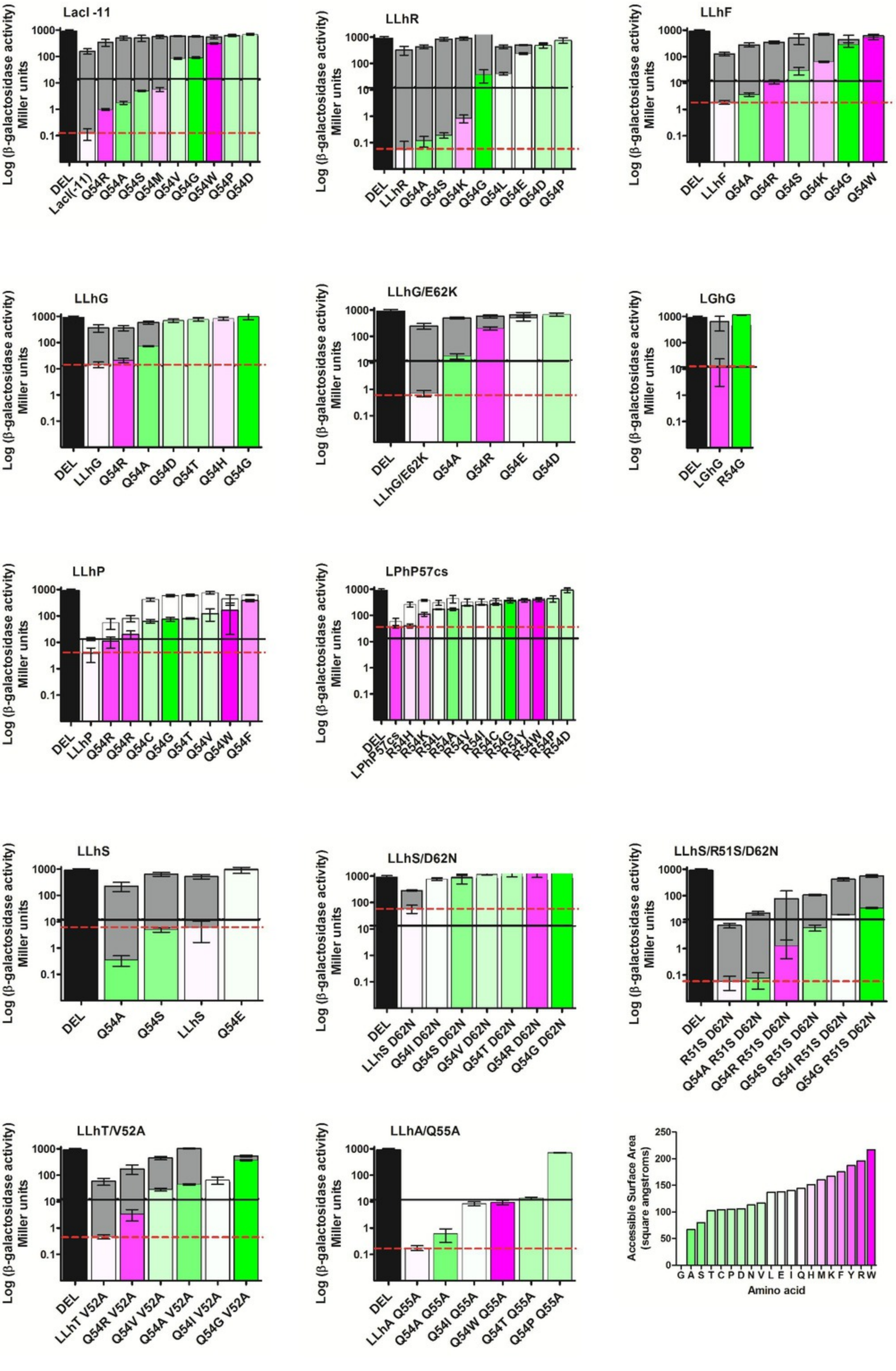

Figure S49: Physicochemical trends: Position 54, Accessible surface area

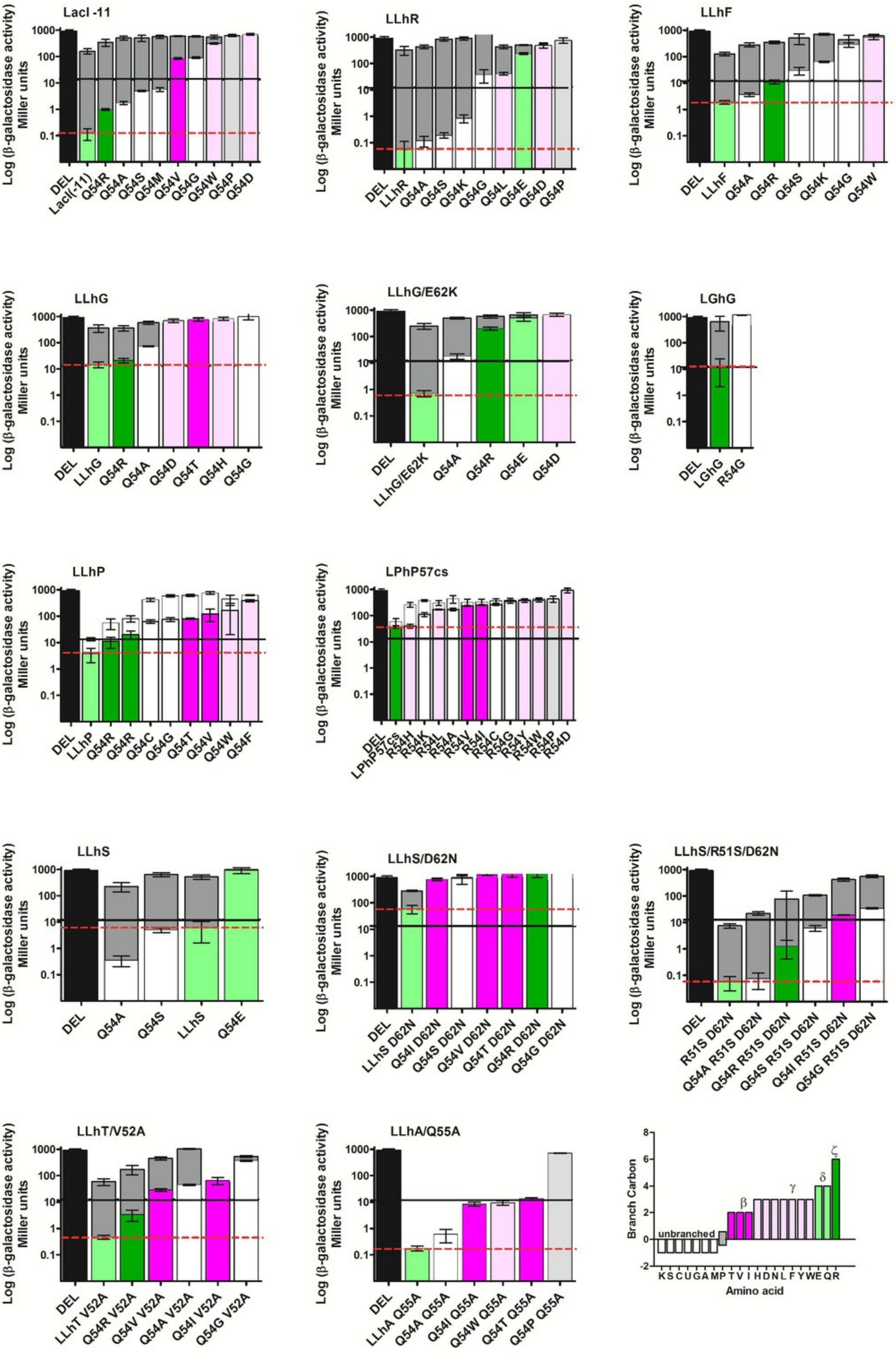

Figure S50: Physicochemical trends: Position 54, Side chain branching

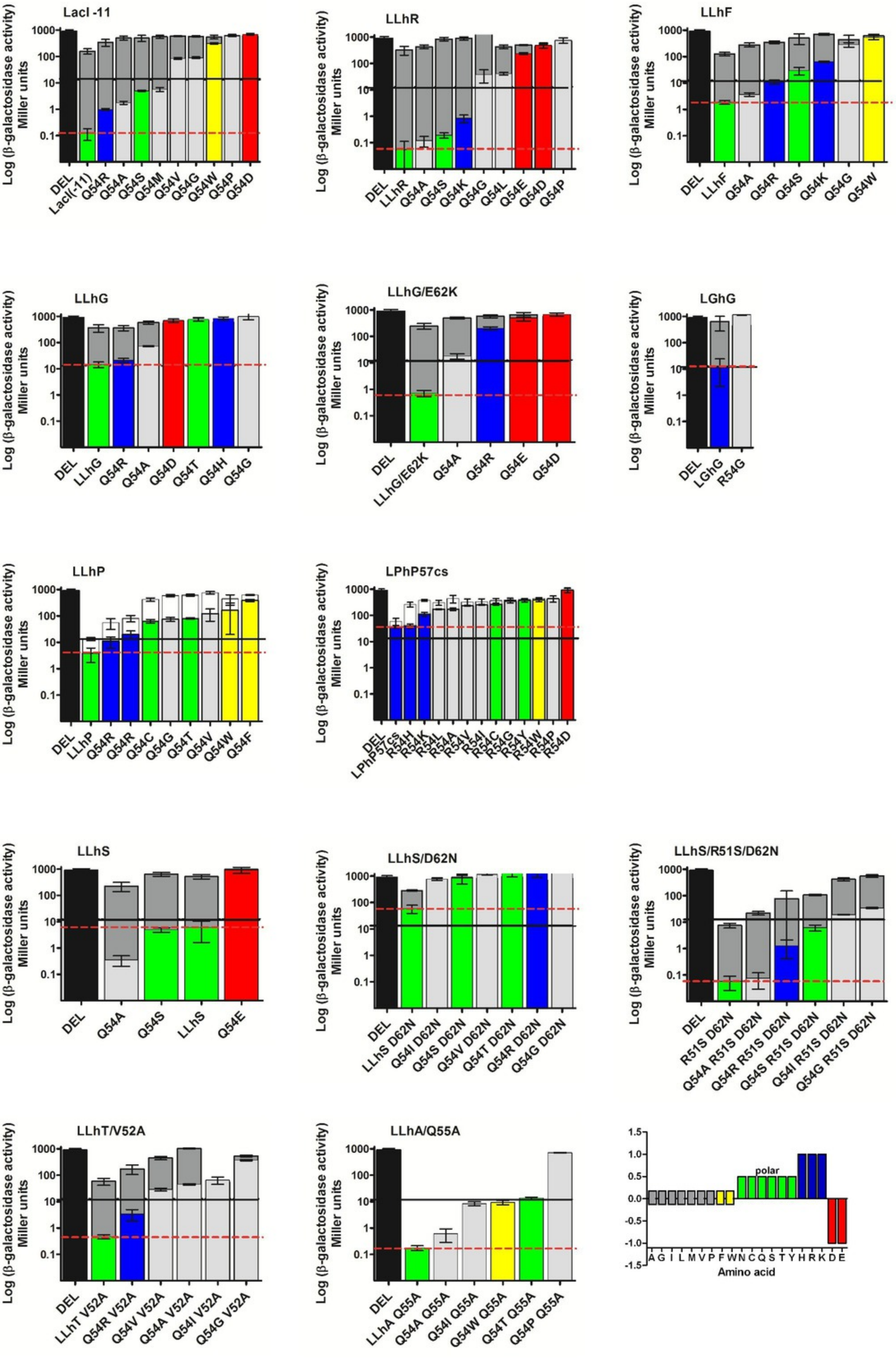

Figure S51: Physicochemical trends: Position 54, Charge/polarity/aromaticity

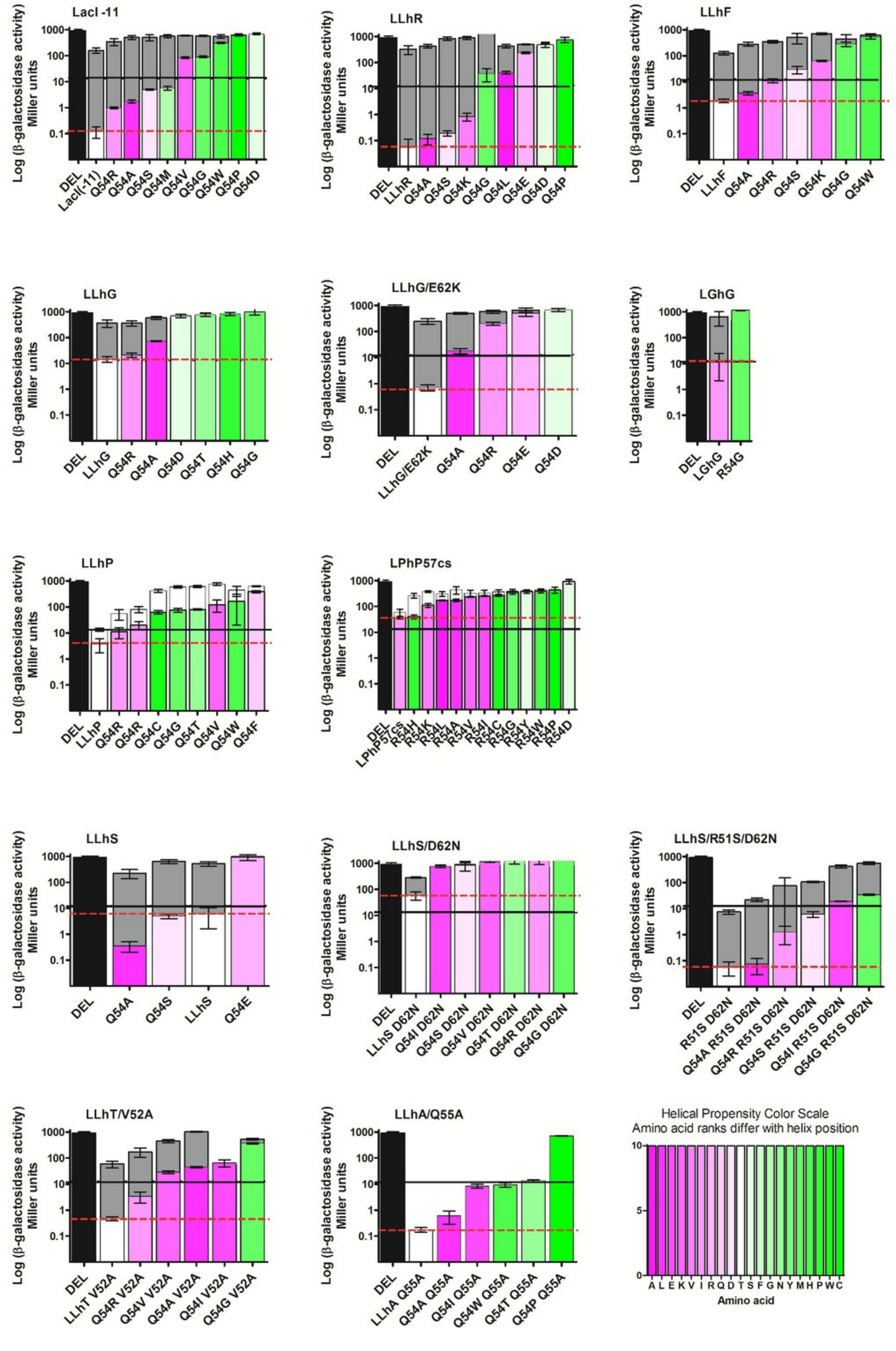

Figure S52: Physicochemical trends: Position 54, Helical propensity (C4 scale)

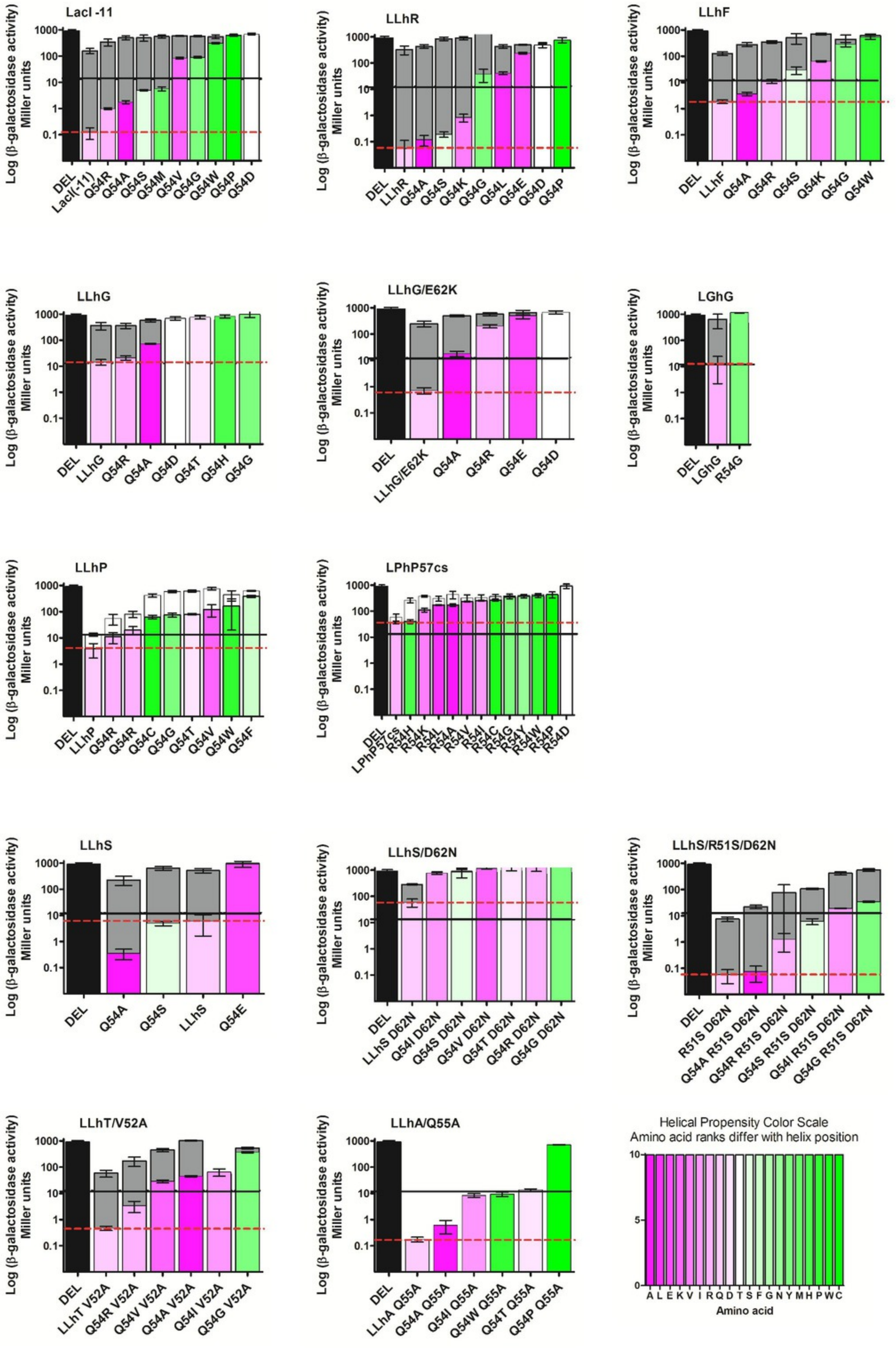

Figure S53: Physicochemical trends: Position 54, Helical propensity (Midpoint scale)

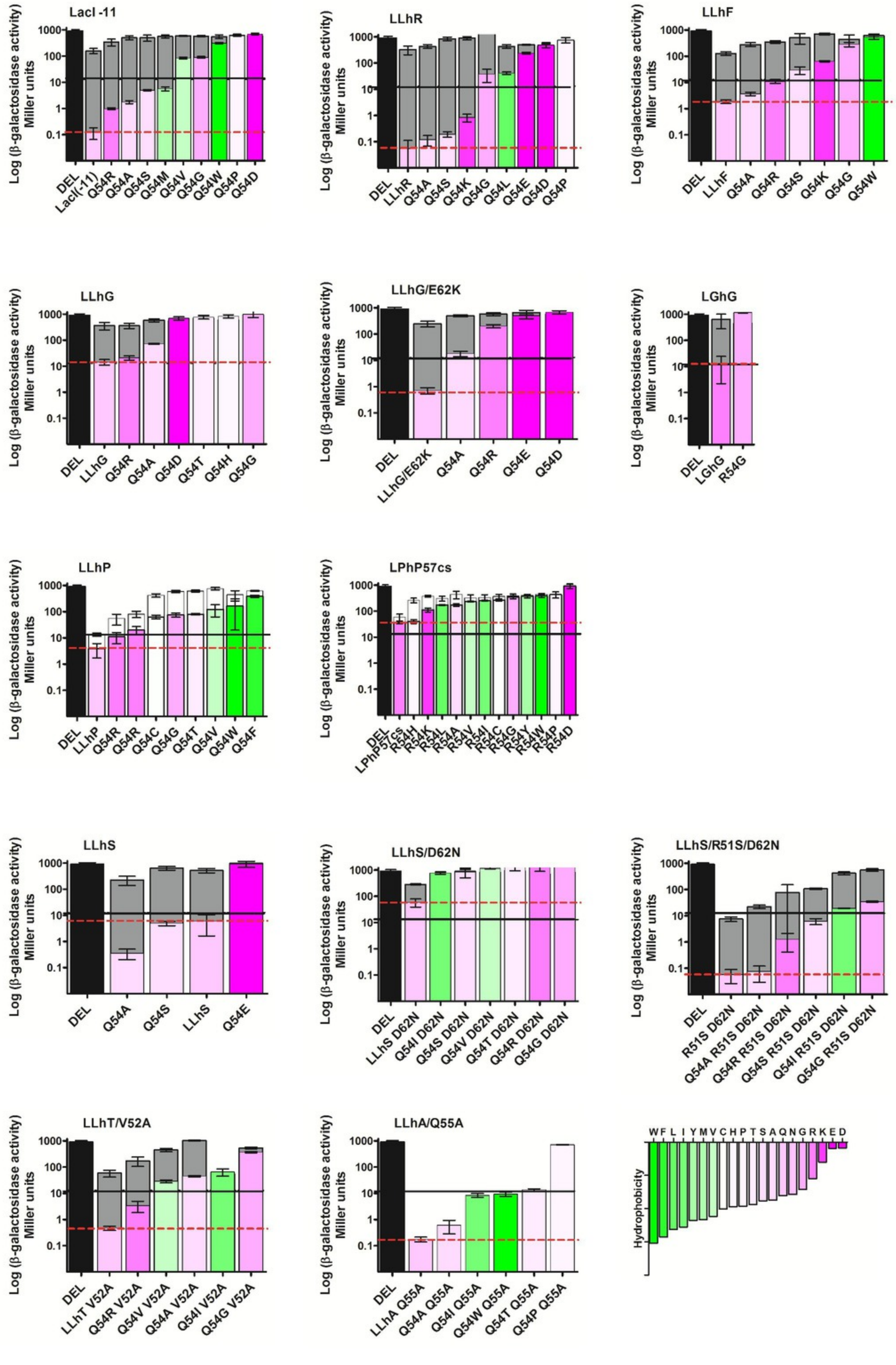

Figure S54: Physicochemical trends: Position 54, Hydrophobicity
