# Supplementary material for: Rheostats and Toggle Switches for Modulating Protein Function
Source: PLoS One. 2013 Dec 30;8(12):e83502. doi: 10.1371/journal.pone.0083502 (PMC3875437; doi:10.1371/journal.pone.0083502)
Supplement: Data S6 — Supporting figures. Figures S55–S72. Physico-chemical trends: positions 55–59. Repression assay data for each position were color-coded according to various physico-chemical scales (Table S20 in Data S2). For example, Figures S55–S57 show results for position 55 color-coded by accessible surface area of the free side chain [46], side chain branching, and charge/polarity/aromaticity. The relevant parameter can be determined from the legend in the lower right hand corner of each graph. Positions 50–58 have potential to participate in an alpha helix, and repression assay results were also compared to both average and position-specific helical propensities [47]. For simplicity, only one helix color scale is used as a legend, with magenta corresponding to high propensity and green corresponding to low propensity. Since wild-type LacI and PurR have different length helices [20], we compared multiple helical scales to results for each position. However, no scale showed good correlation with the functional assay among all chimeras. (PDF) [file pone.0083502.s006.pdf]

Supplemental Material for:
Rheostats and toggle switches for modulating protein function
File 6: Physicochemical trends: Positions 55-59

Meinhardt, Manley, Parente and Swint-Kruse

List of Figures

S55 Physicochemical trends: Position 55, Accessible surface area . . . 3
S56 Physicochemical trends: Position 55, Side chain branching . . . 4
S57 Physicochemical trends: Position 55, Charge/polarity/aromaticity . . . 5
S58 Physicochemical trends: Position 55, Helical propensity (C3 scale) . . . 6
S59 Physicochemical trends: Position 55, Helical propensity (C4 scale) . . . 7
S60 Physicochemical trends: Position 55, Hydrophobicity . . . 8
S61 Physicochemical trends: Position 58, Accessible surface area . . . 9
S62 Physicochemical trends: Position 58, Side chain branching . . . 10
S63 Physicochemical trends: Position 58, Charge/polarity/aromaticity . . . 11
S64 Physicochemical trends: Position 58, Helical propensity (C1 scale) . . . 12
S65 Physicochemical trends: Position 58, Helical propensity C_{cap} scale) . . . 13
S66 Physicochemical trends: Position 58, Hydrophobicity . . . 14
S67 Physicochemical trends: Position 59, Accessible surface area . . . 15
S68 Physicochemical trends: Position 59, Side chain branching . . . 16
S69 Physicochemical trends: Position 59, Charge/polarity/aromaticity . . . 17
S70 Physicochemical trends: Position 59, Helical propensity (C' scale) . . . 18
S71 Physicochemical trends: Position 59, Helical propensity (C_{cap} scale) . . . 19
S72 Physicochemical trends: Position 59, Hydrophobicity . . . 20

Figures S55-S72. Physico-chemical trends: positions 55-59. Repression assay data for each position were color-coded according to various physico-chemical scales. For example, Figures S55-S57 show results for position 55 color-coded by accessible surface area of the free side chain [46], side chain branching, and charge/polarity/aromaticity. The relevant parameter can be determined from the legend in the lower right hand corner of each graph. Positions 50-58 have potential to participate in an alpha helix, and repression assay results were also compared to both average and position-specific helical propensities [47]. For simplicity, only one helix color scale is used as a legend, with magenta corresponding to high propensity and green corresponding to low propensity. Since wild-type LacI and PurR have different length helices [20], we compared multiple helical scales to results for each position. However, no scale showed good correlation with the functional assay among all chimeras.

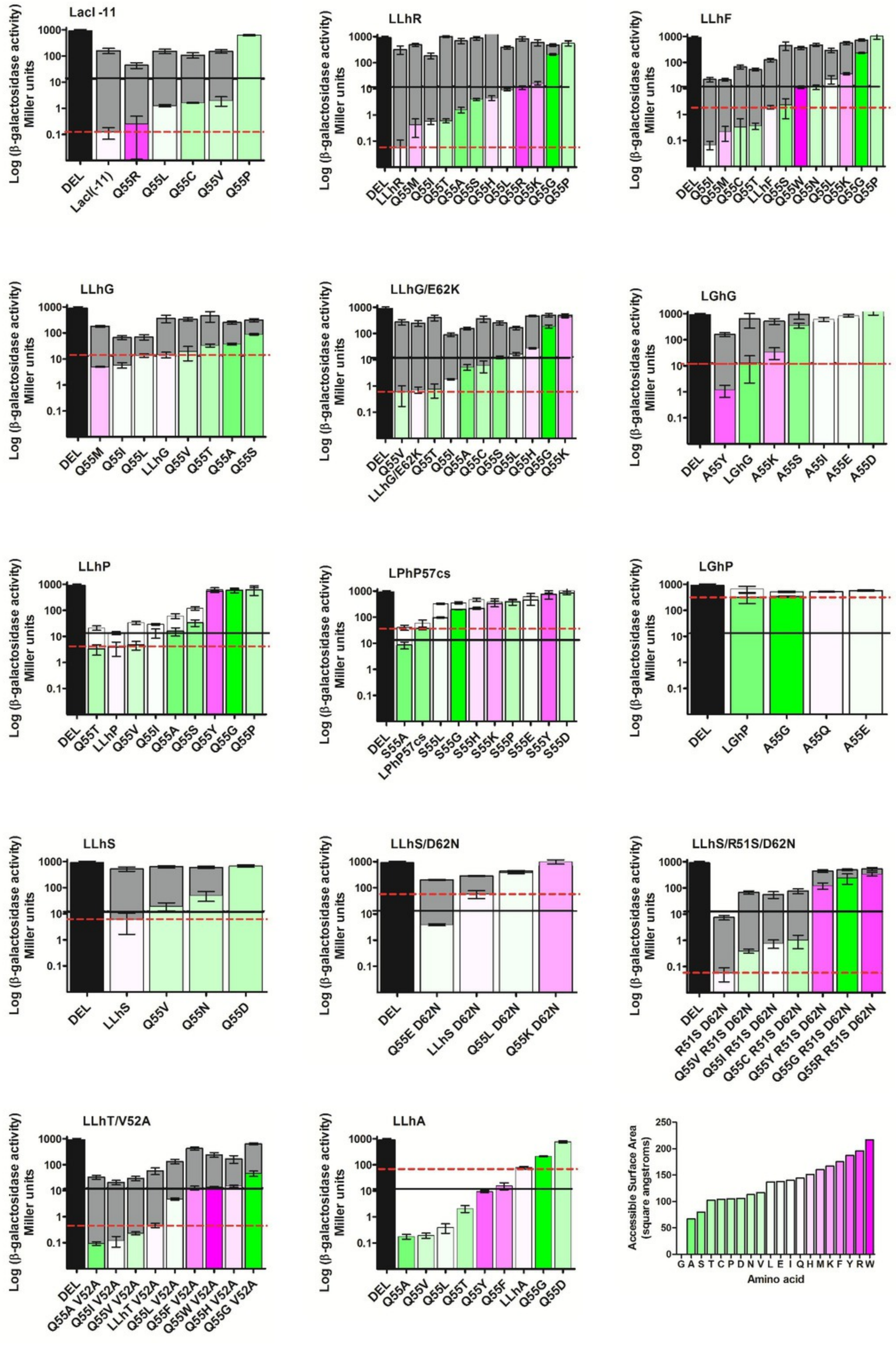

Figure S55: Physicochemical trends: Position 55, Accessible surface area

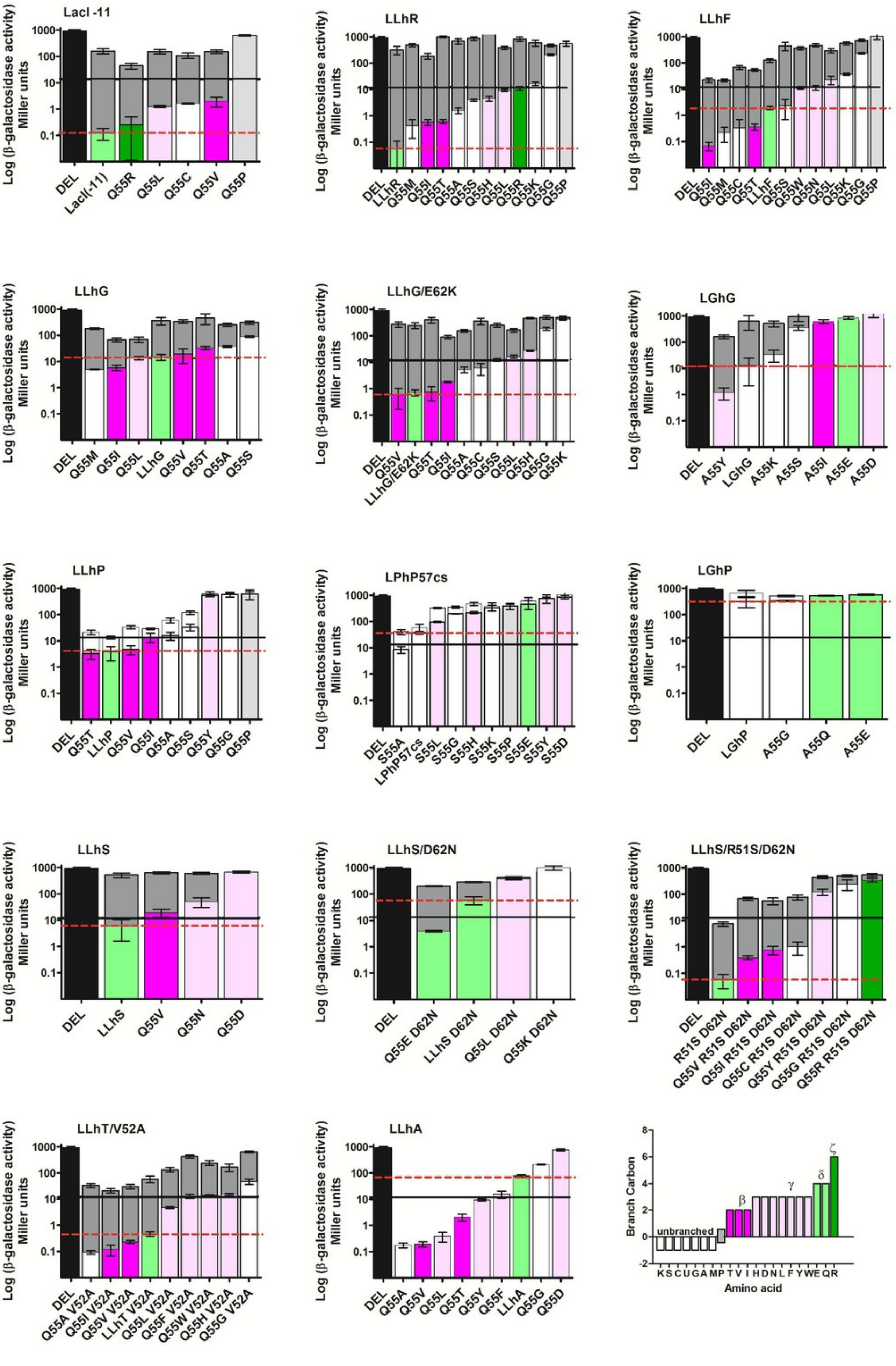

Figure S56: Physicochemical trends: Position 55, Side chain branching

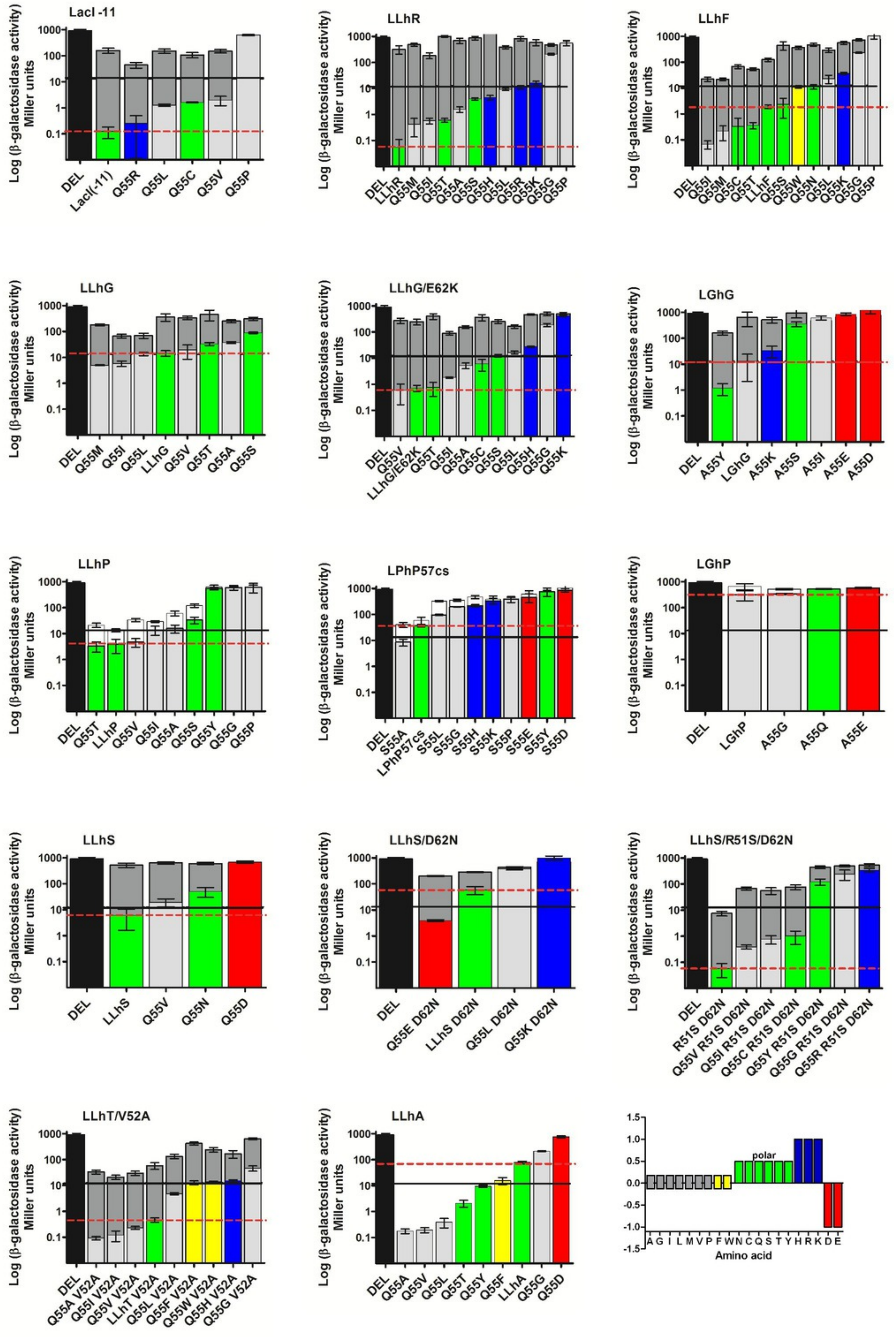

Figure S57: Physicochemical trends: Position 55, Charge/polarity/aromaticity

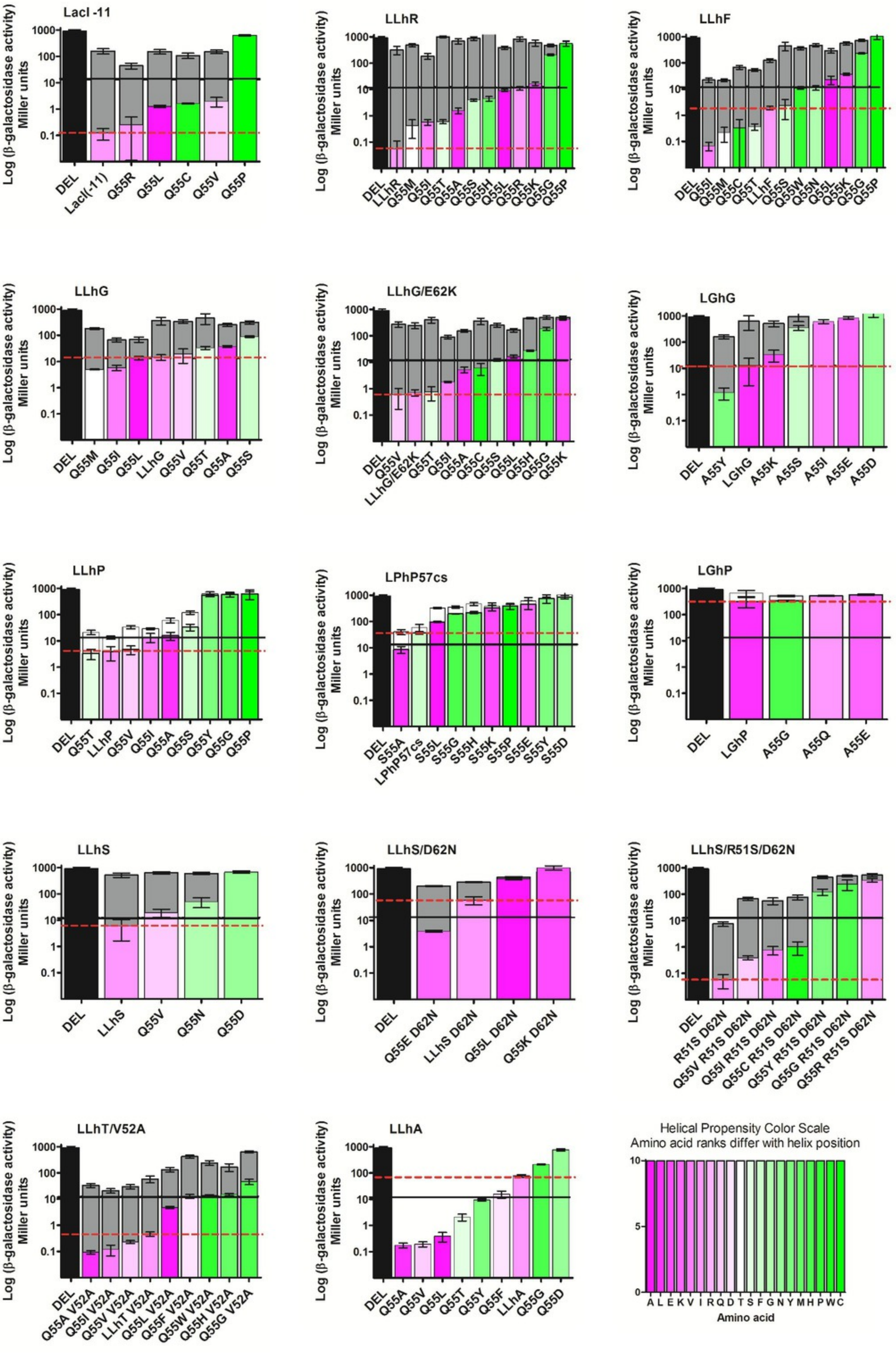

Figure S58: Physicochemical trends: Position 55, Helical propensity (C3 scale)

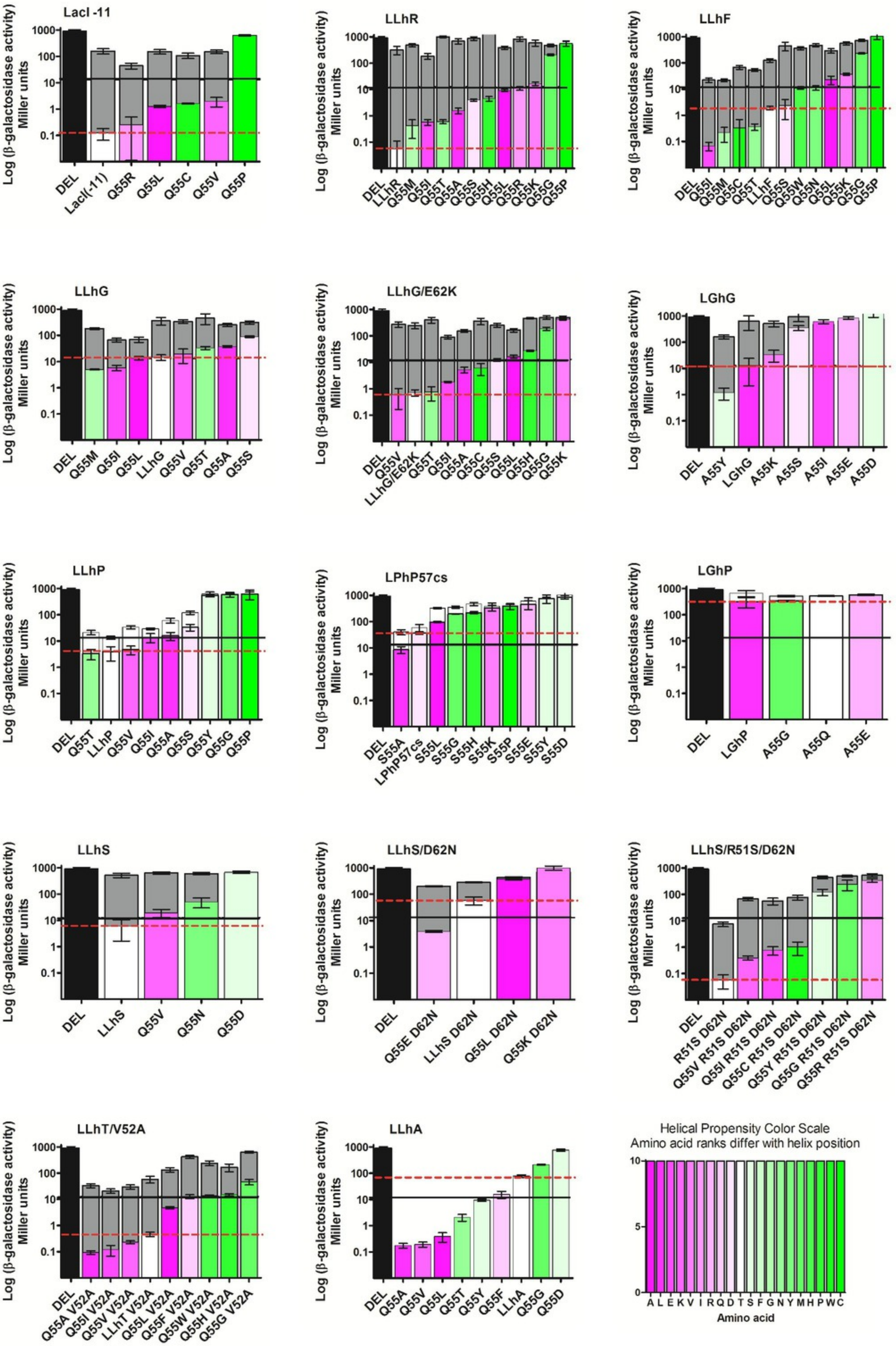

Figure S59: Physicochemical trends: Position 55, Helical propensity (C4 scale)

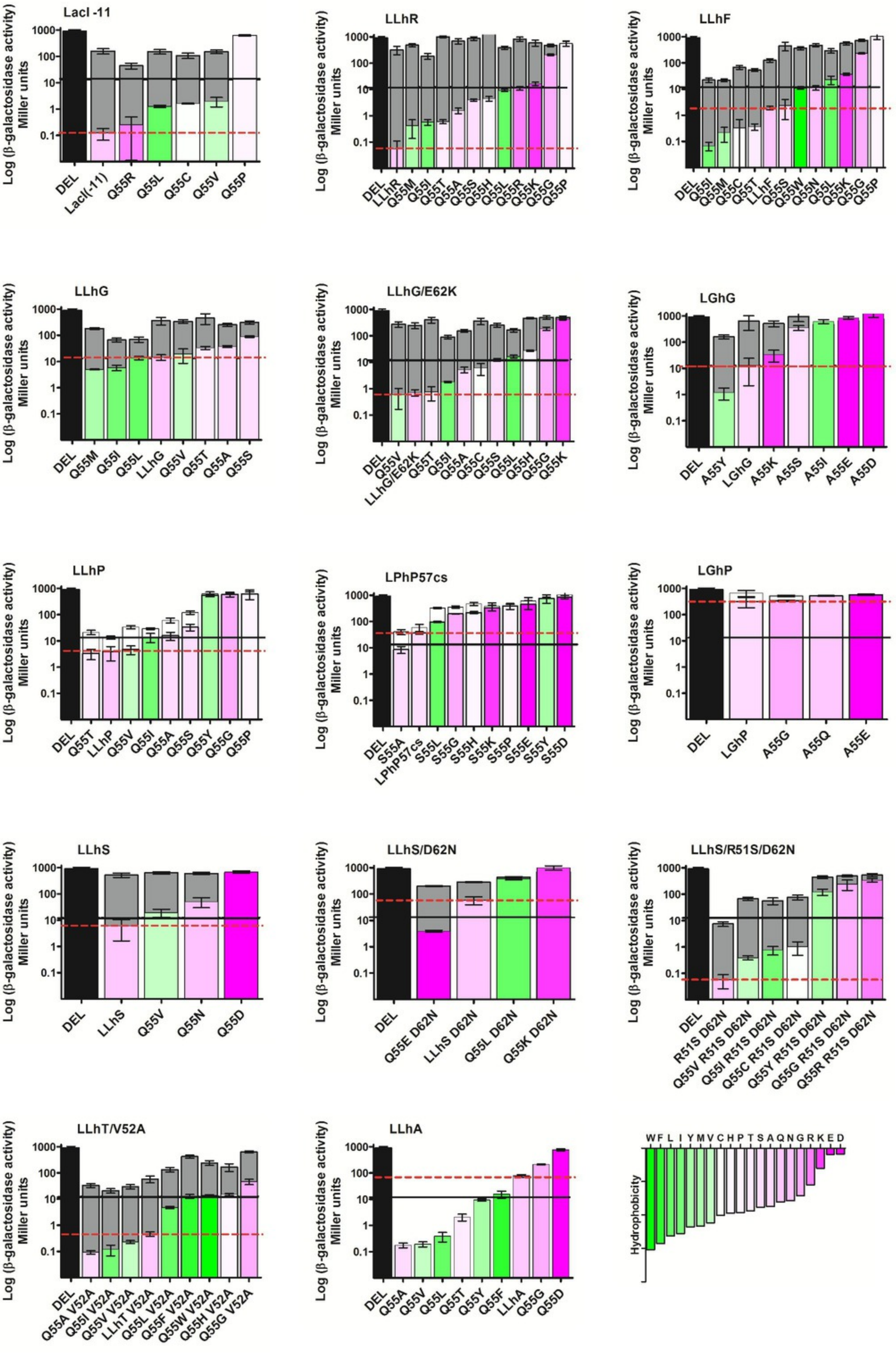

Figure S60: Physicochemical trends: Position 55, Hydrophobicity

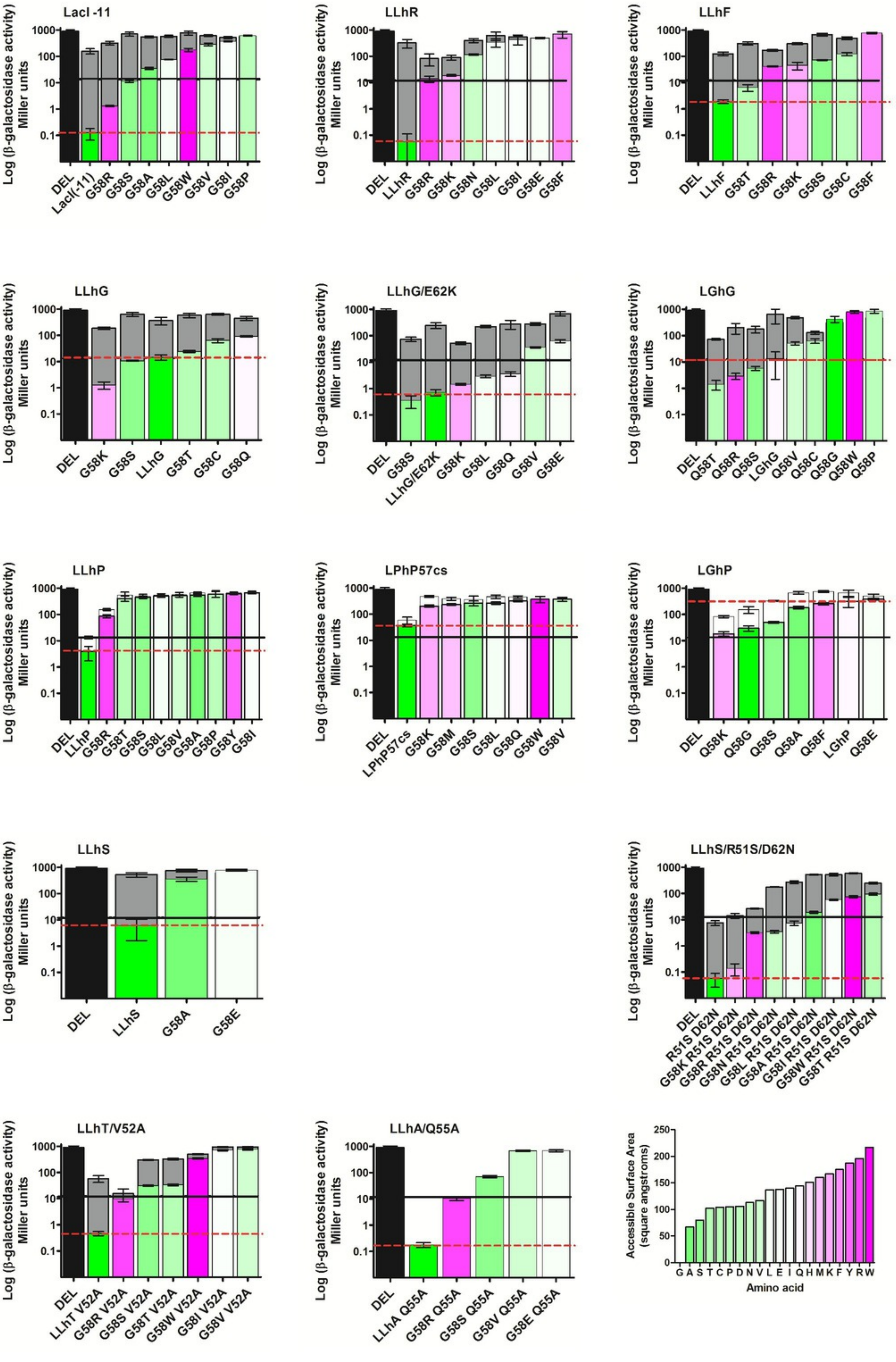

Figure S61: Physicochemical trends: Position 58, Accessible surface area

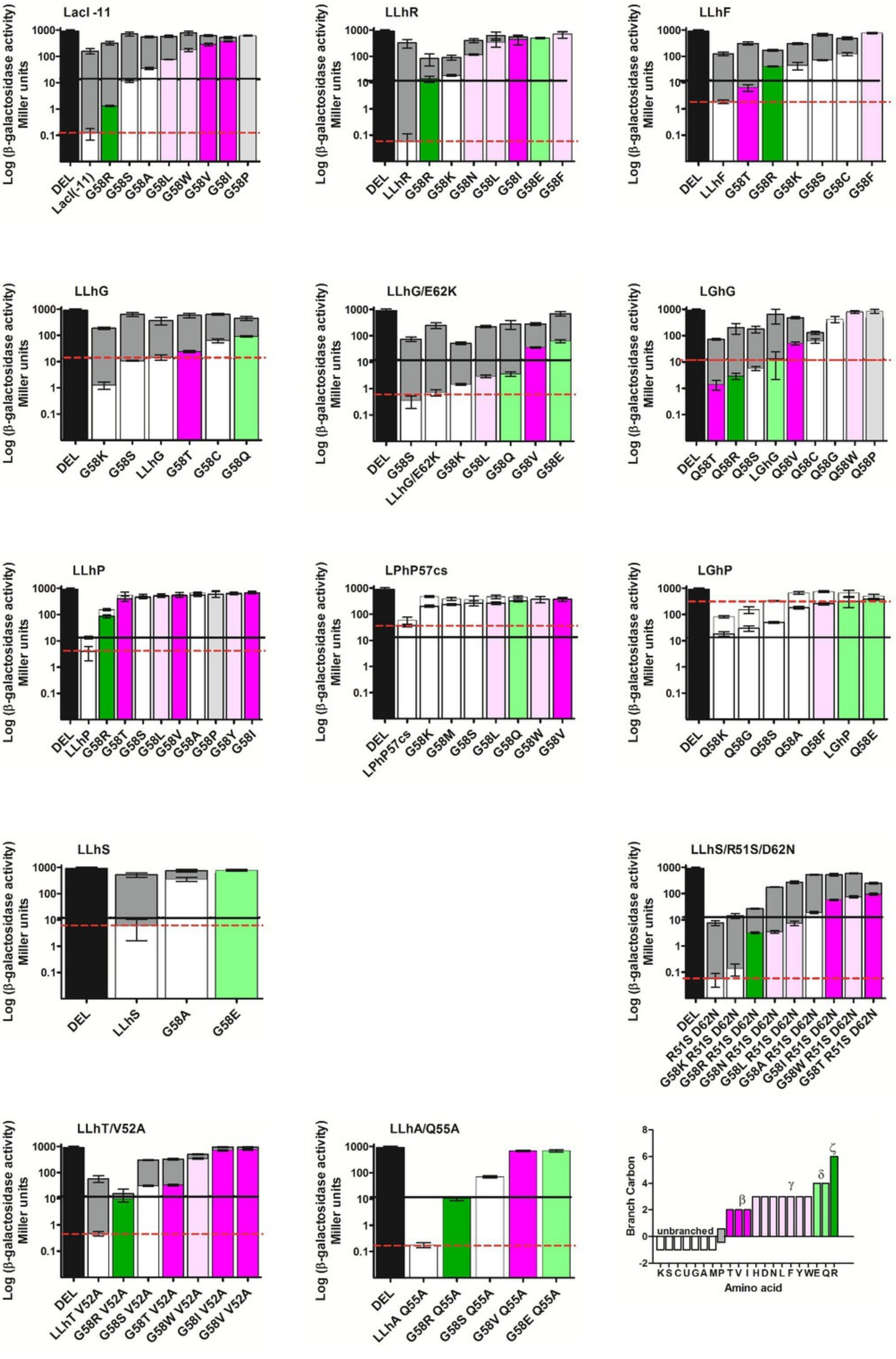

Figure S62: Physicochemical trends: Position 58, Side chain branching

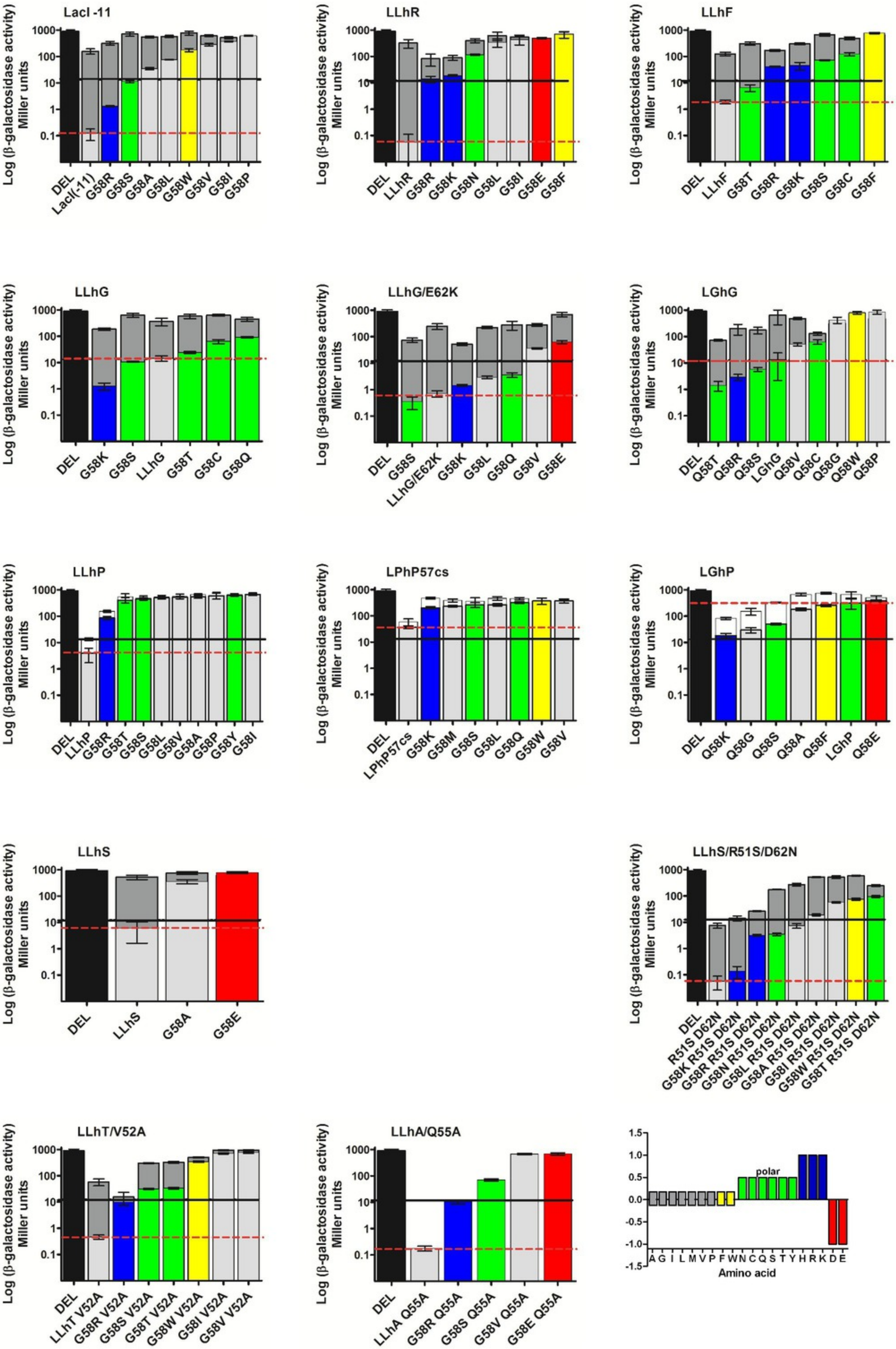

Figure S63: Physicochemical trends: Position 58, Charge/polarity/aromaticity

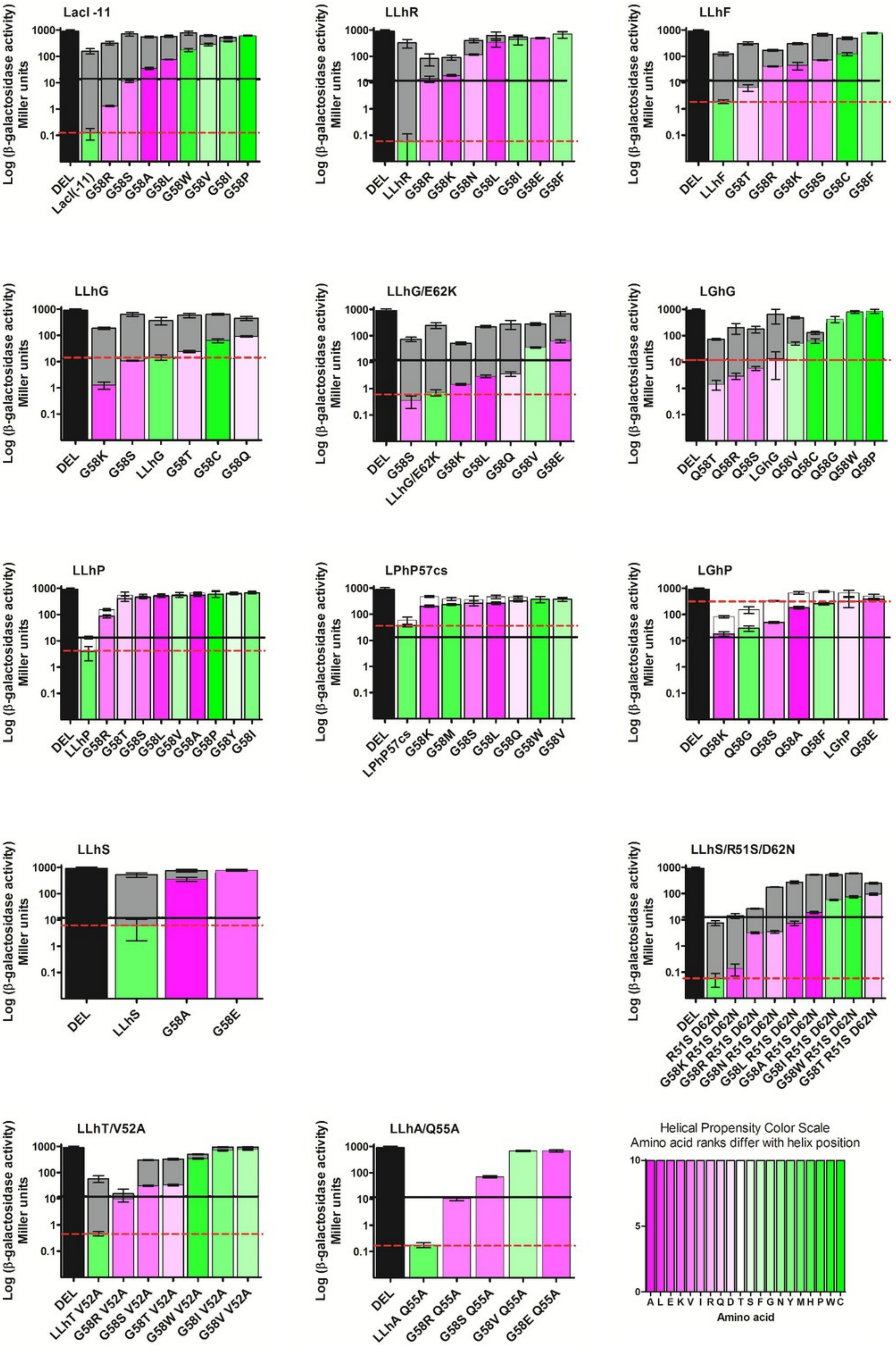

Figure S64: Physicochemical trends: Position 58, Helical propensity (C1 scale)

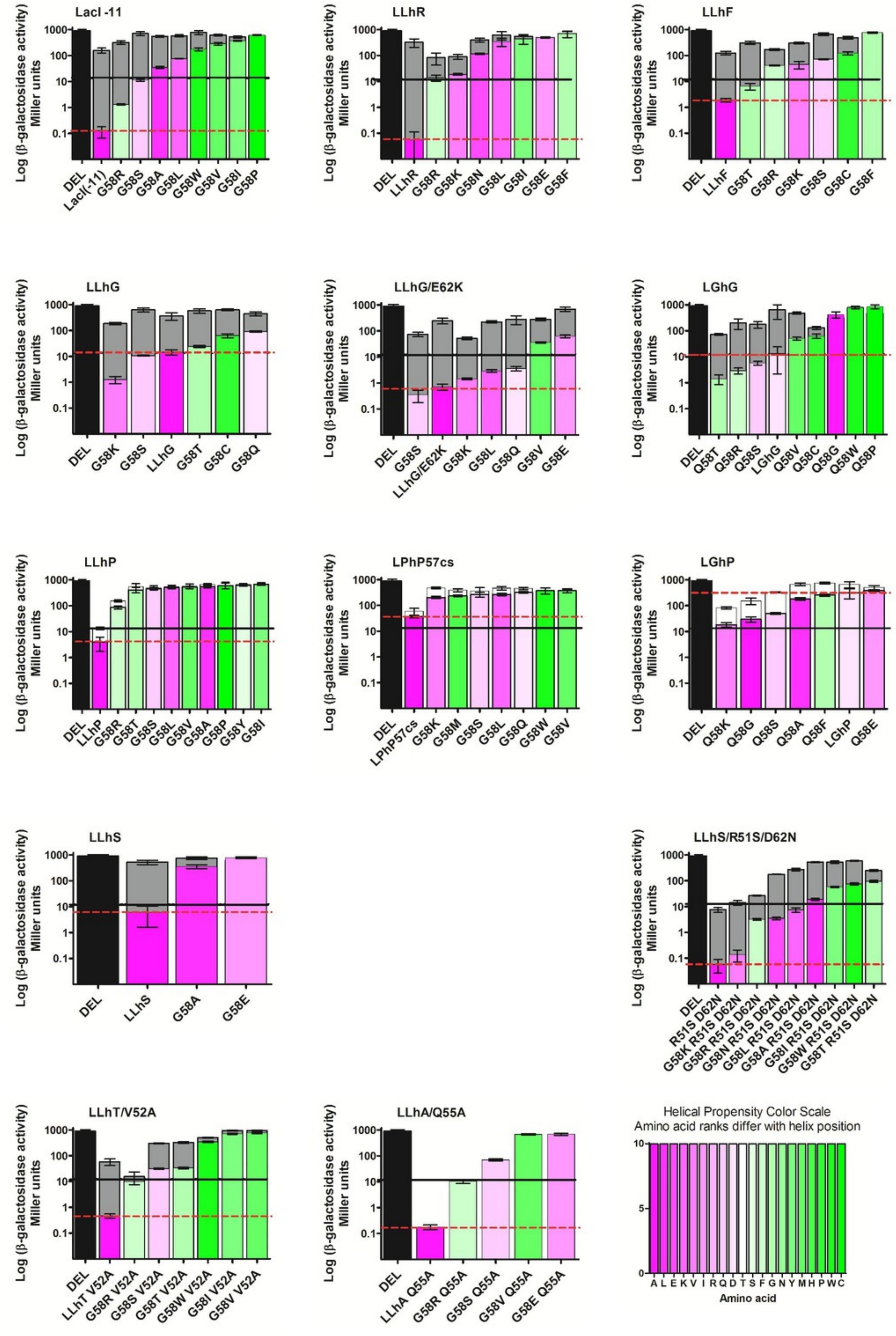

Figure S65: Physicochemical trends: Position 58, Helical propensity C_{cap} scale)

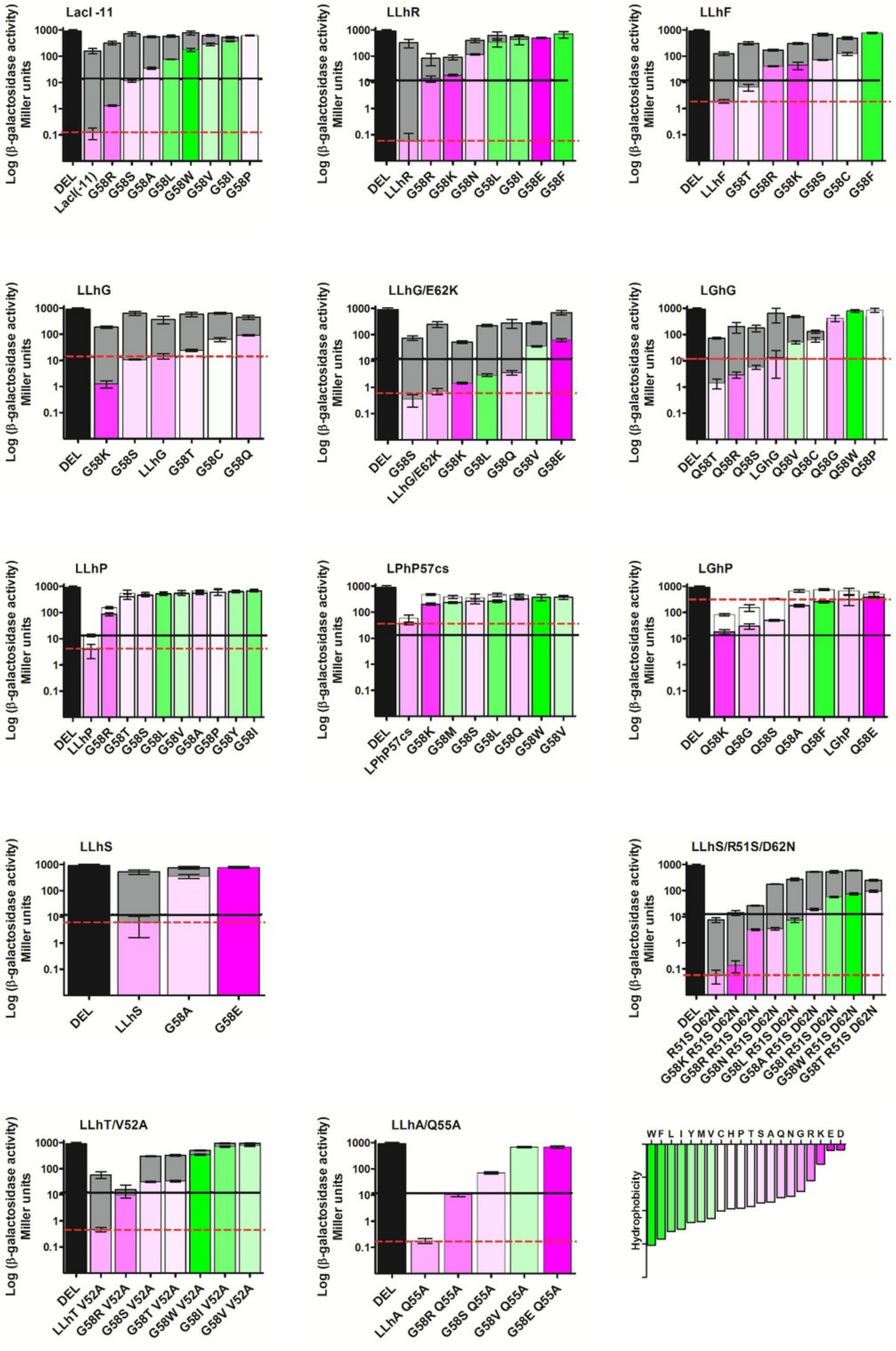

Figure S66: Physicochemical trends: Position 58, Hydrophobicity

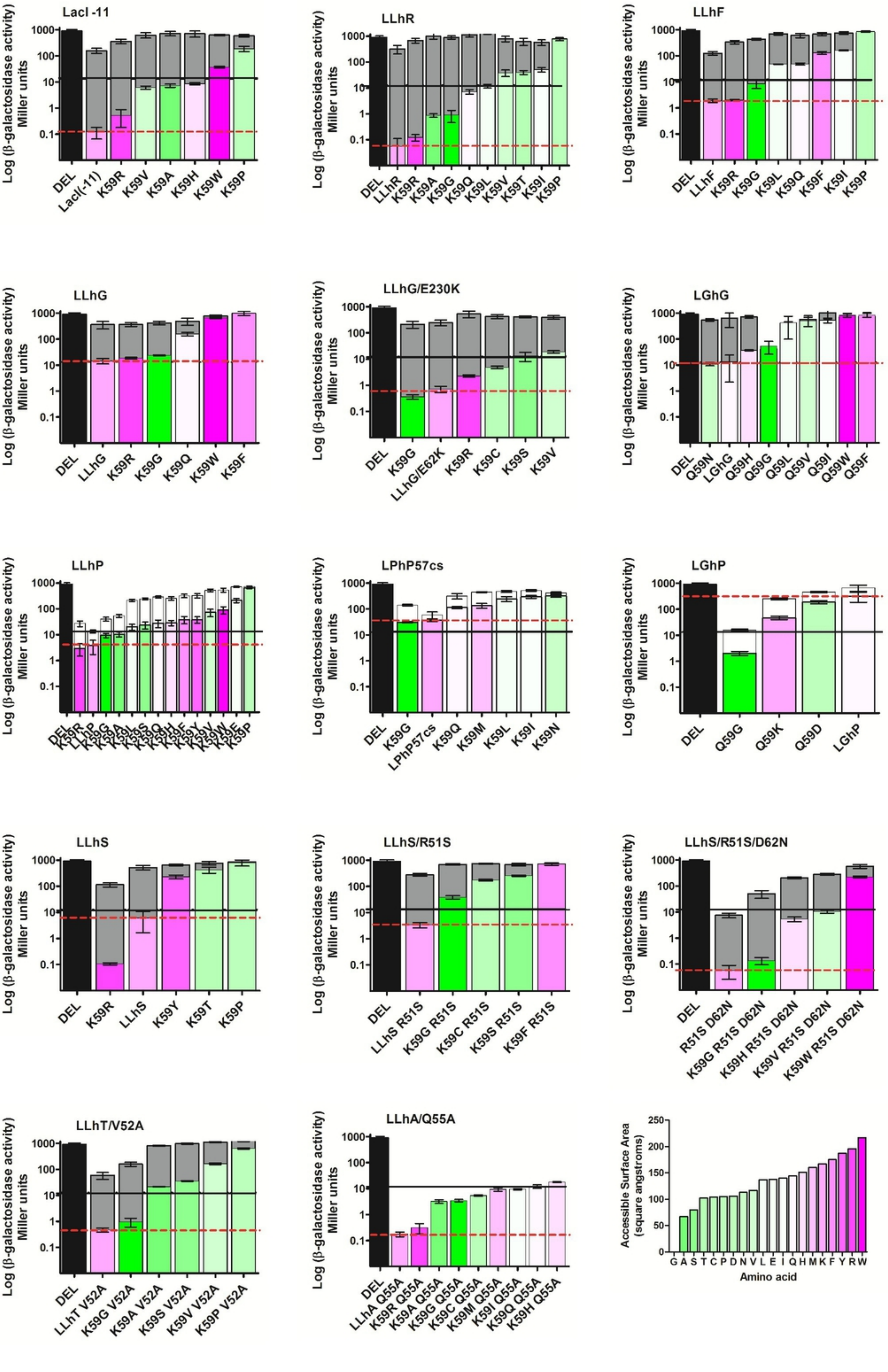

Figure S67: Physicochemical trends: Position 59, Accessible surface area

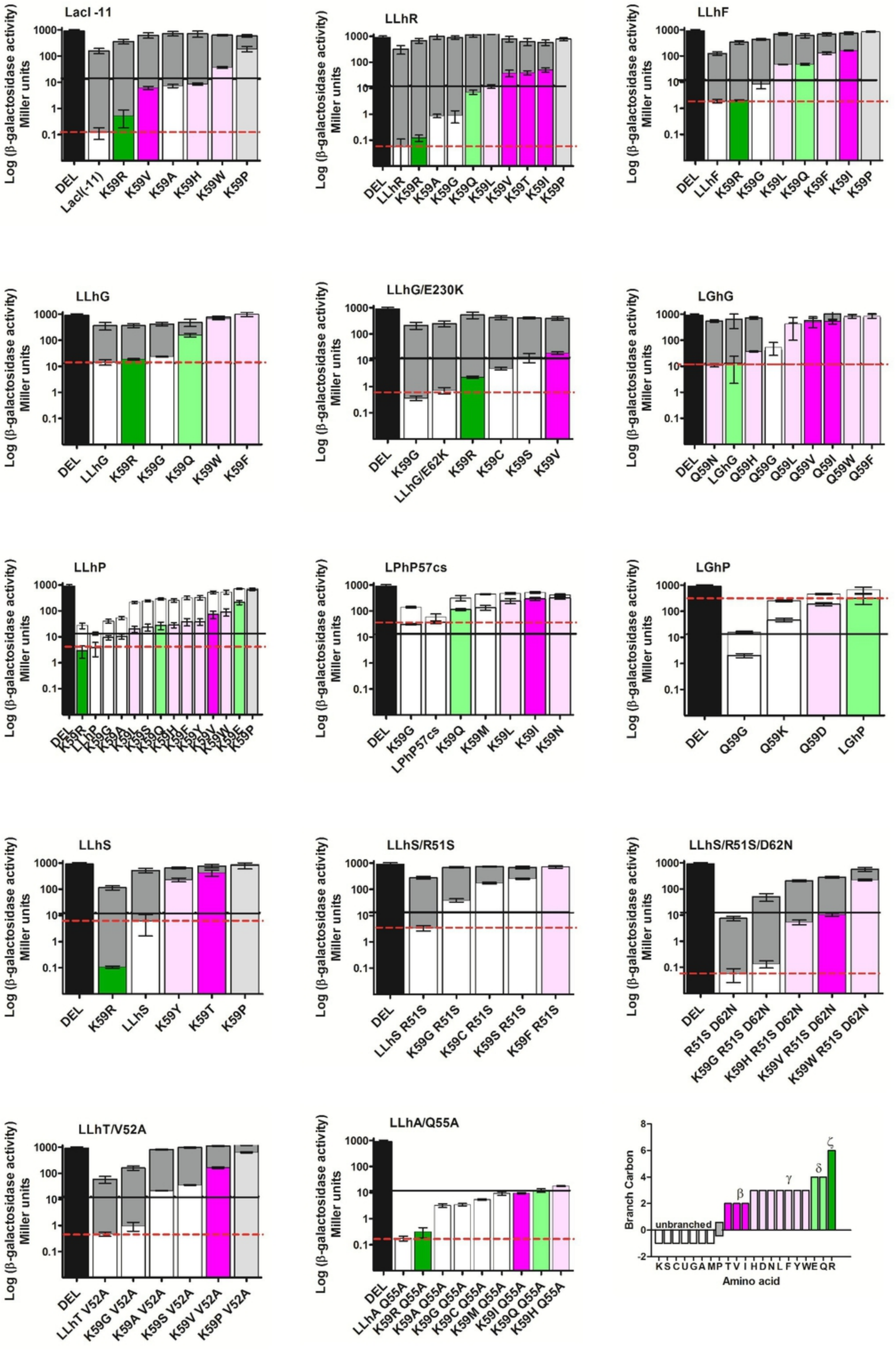

Figure S68: Physicochemical trends: Position 59, Side chain branching

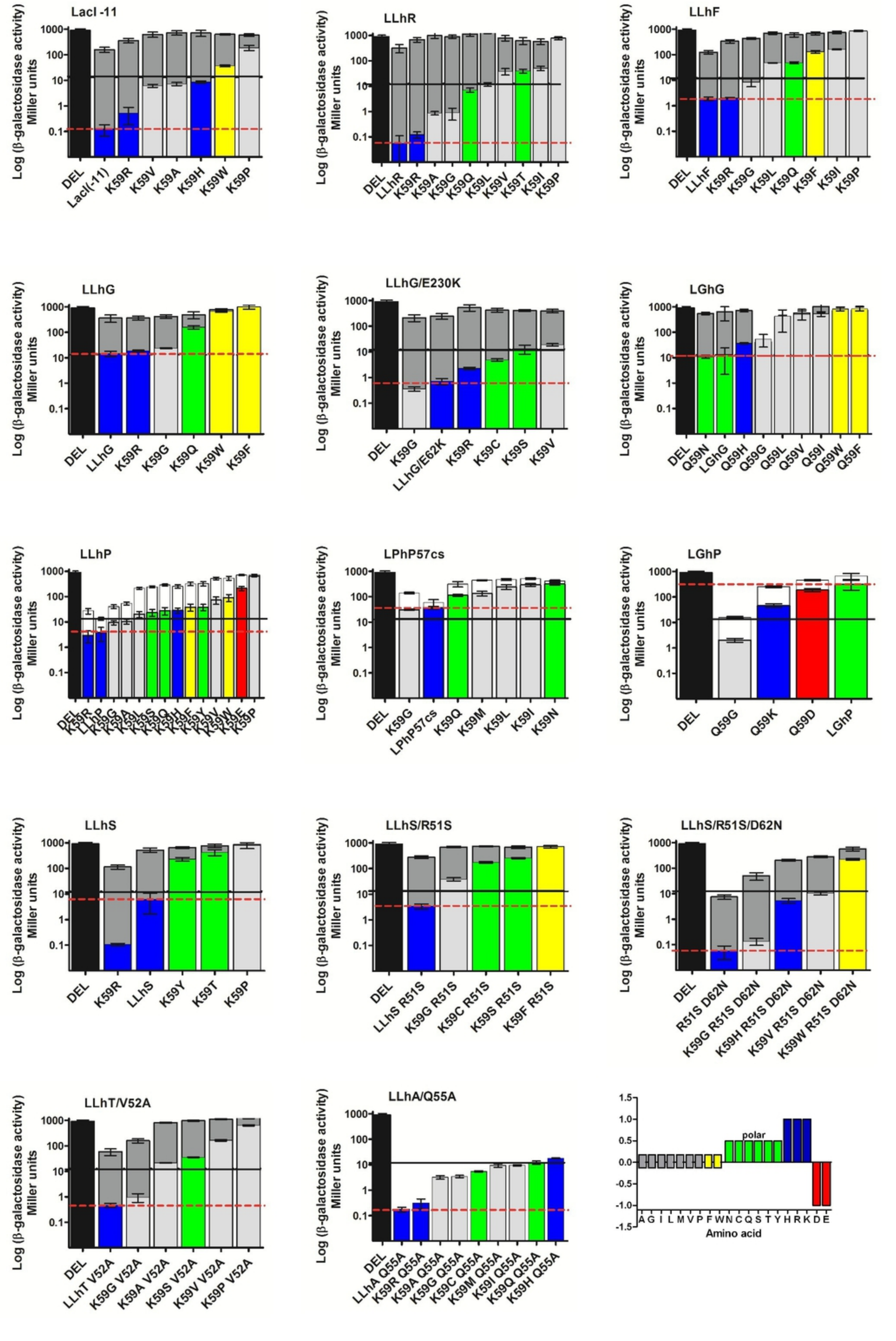

Figure S69: Physicochemical trends: Position 59, Charge/polarity/aromaticity

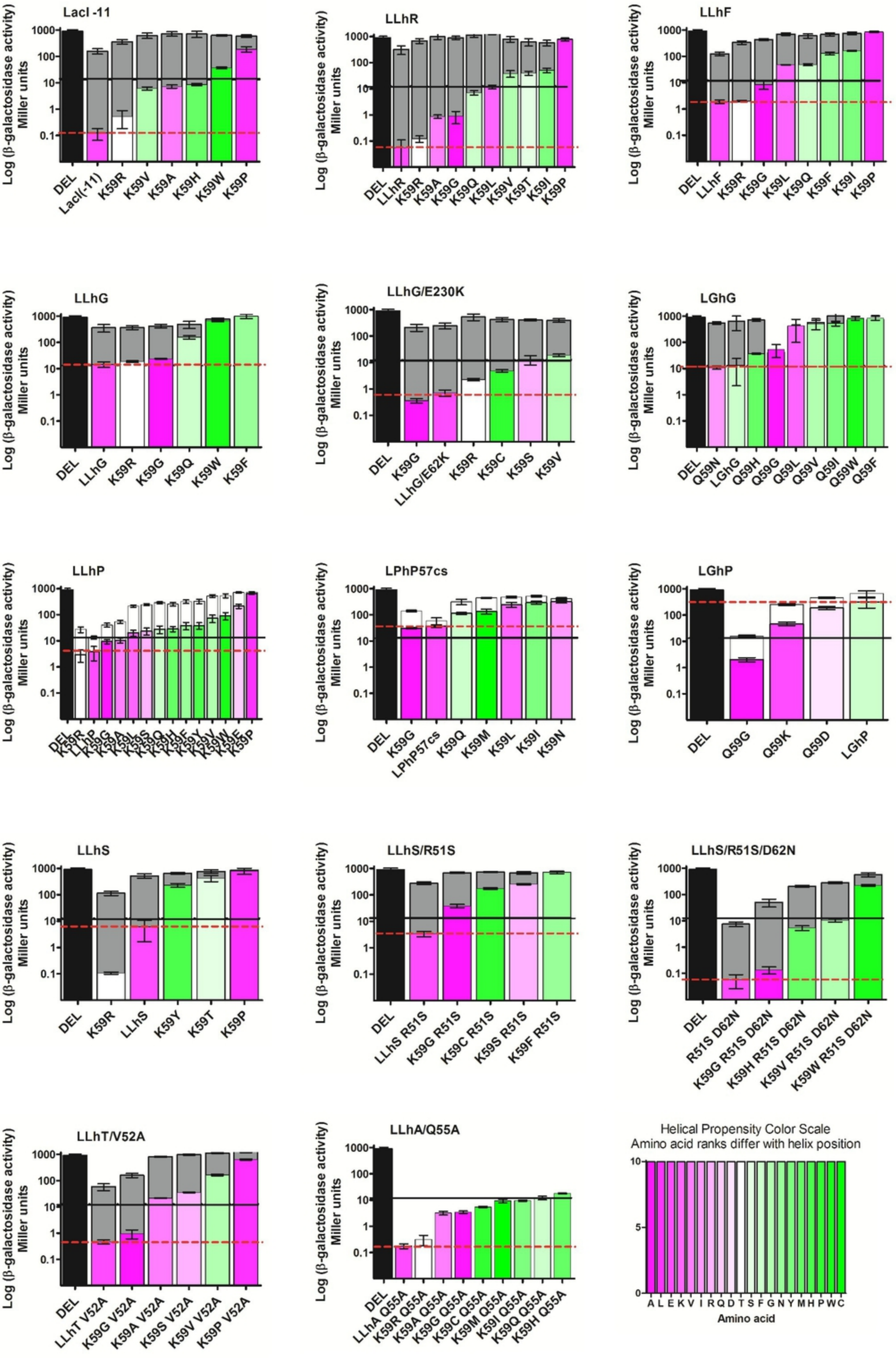

Figure S70: Physicochemical trends: Position 59, Helical propensity (C′ scale)

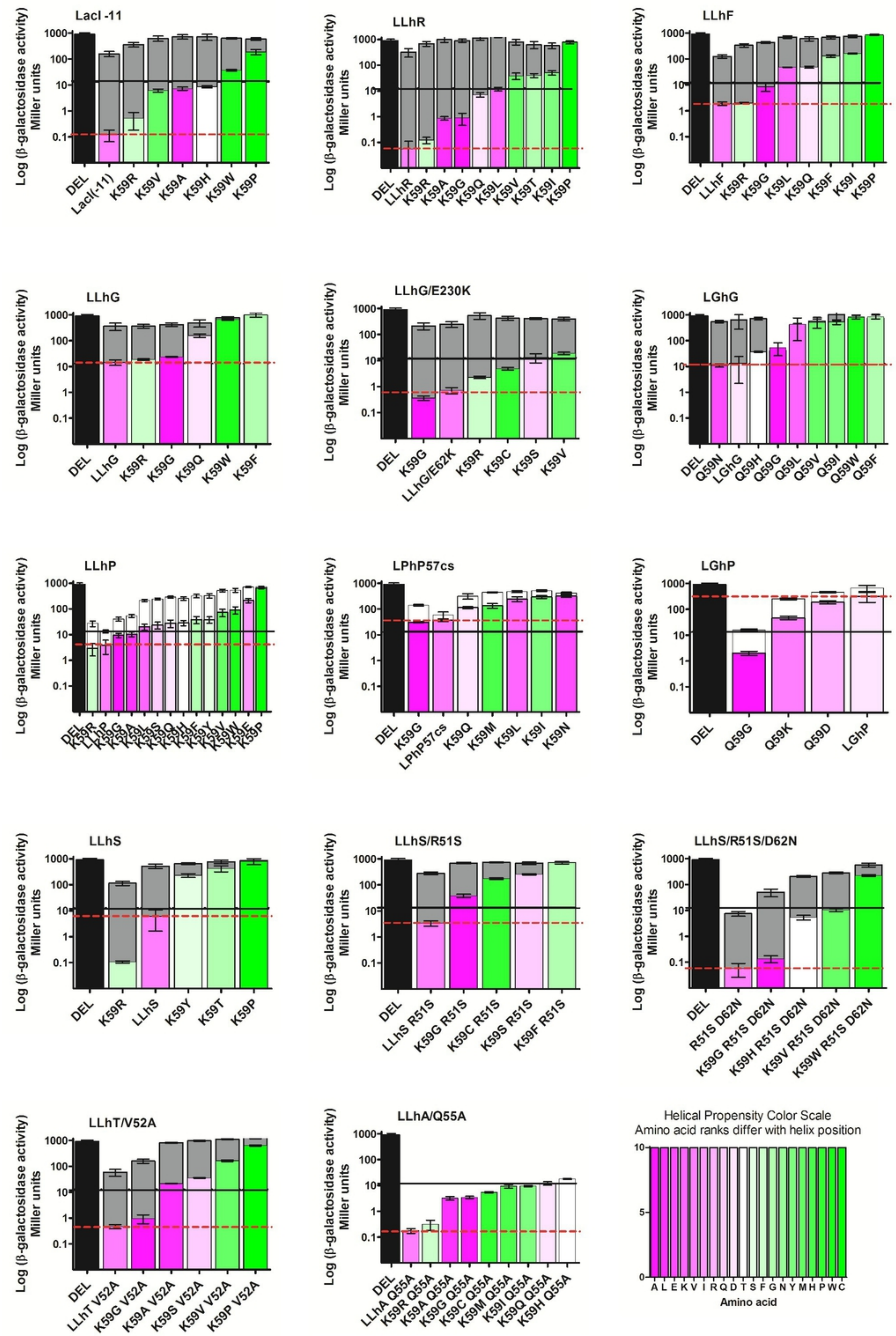

Figure S71: Physicochemical trends: Position 59, Helical propensity (C$_{cap}$ scale)

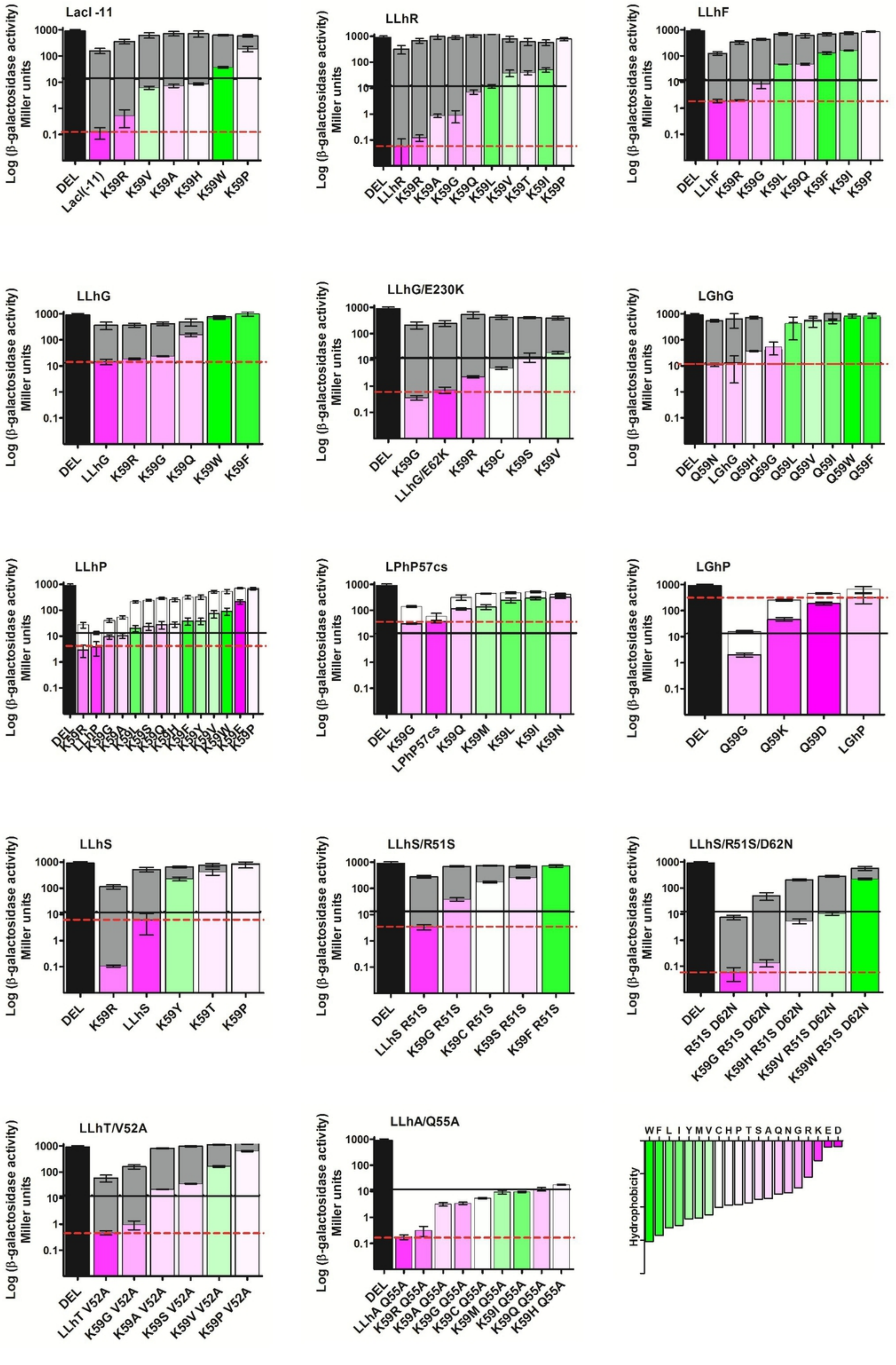

Figure S72: Physicochemical trends: Position 59, Hydrophobicity
