# Supplementary material for: Rheostats and Toggle Switches for Modulating Protein Function
Source: PLoS One. 2013 Dec 30;8(12):e83502. doi: 10.1371/journal.pone.0083502 (PMC3875437; doi:10.1371/journal.pone.0083502)
Supplement: Data S7 — Supporting figures. Figures S73–S87. Physico-chemical trends: positions 60–62. Repression assay data for each position were color-coded according to various physico-chemical scales (Table S20 in Data S2). For example, Figures S73–S75 show results for position 60 color-coded by accessible surface area of the free side chain [46], side chain branching, and charge/polarity/aromaticity. The relevant parameter can be determined from the legend in the lower right hand corner of each graph. (Note that the legend is placed in a middle location for position 62, in order to place results for LLhT and LLhT/V52A in close proximity). (PDF) [file pone.0083502.s007.pdf]

Supplemental Material for:
Rheostats and toggle switches for modulating protein function
File 7: Physicochemical trends: Positions 60-62

Meinhardt, Manley, Parente and Swint-Kruse

List of Figures

S73 Physicochemical trends: Position 60, Accessible surface area . . . 3
S74 Physicochemical trends: Position 60, Side chain branching . . . 4
S75 Physicochemical trends: Position 60, Charge/polarity/aromaticity . . . 5
S76 Physicochemical trends: Position 60, Helical propensity (C′ scale) . . . 6
S77 Physicochemical trends: Position 60, Helical propensity (C″ scale) . . . 7
S78 Physicochemical trends: Position 60, Hydrophobicity . . . 8
S79 Physicochemical trends: Position 61, Accessible surface area . . . 9
S80 Physicochemical trends: Position 61, Side chain branching . . . 10
S81 Physicochemical trends: Position 61, Charge/polarity/aromaticity . . . 11
S82 Physicochemical trends: Position 61, Helical propensity (C″ scale) . . . 12
S83 Physicochemical trends: Position 61, Hydrophobicity . . . 13
S84 Physicochemical trends: Position 62, Accessible surface area . . . 14
S85 Physicochemical trends: Position 62, Side chain branching . . . 15
S86 Physicochemical trends: Position 62, Charge/polarity/aromaticity . . . 16
S87 Physicochemical trends: Position 62, Hydrophobicity . . . 17

Figures S73-S87. Physico-chemical trends: positions 60-62. Repression assay data for each position were color-coded according to various physico-chemical scales. For example, Figures S73-S75 show results for position 60 color-coded by accessible surface area of the free side chain [46], side chain branching, and charge/polarity/aromaticity. The relevant parameter can be determined from the legend in the lower right hand corner of each graph. (Note that the legend is placed in a middle location for position 62, in order to place results for LLhT and LLhT/V52A in close proximity.)

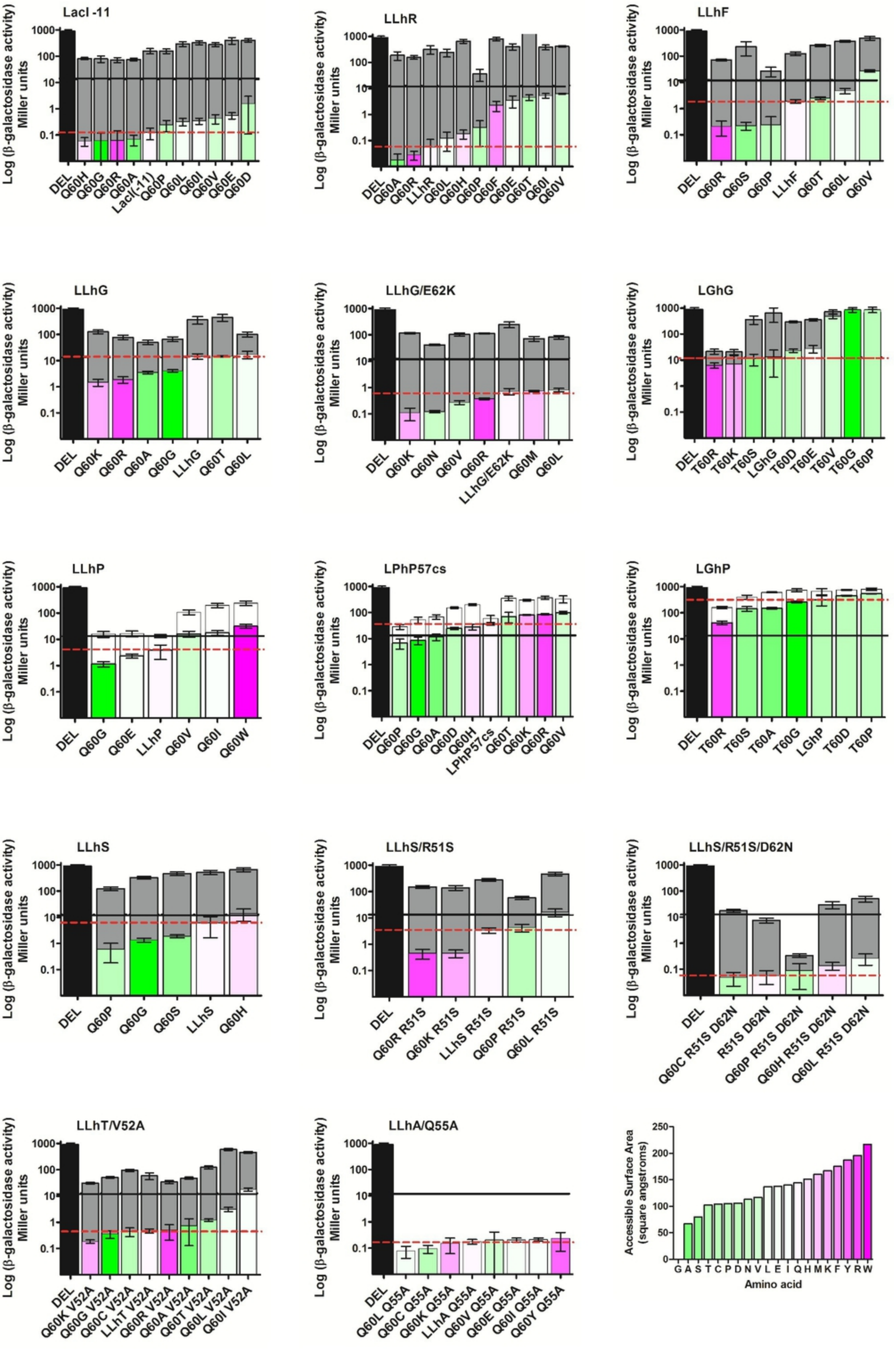

Figure S73: Physicochemical trends: Position 60, Accessible surface area

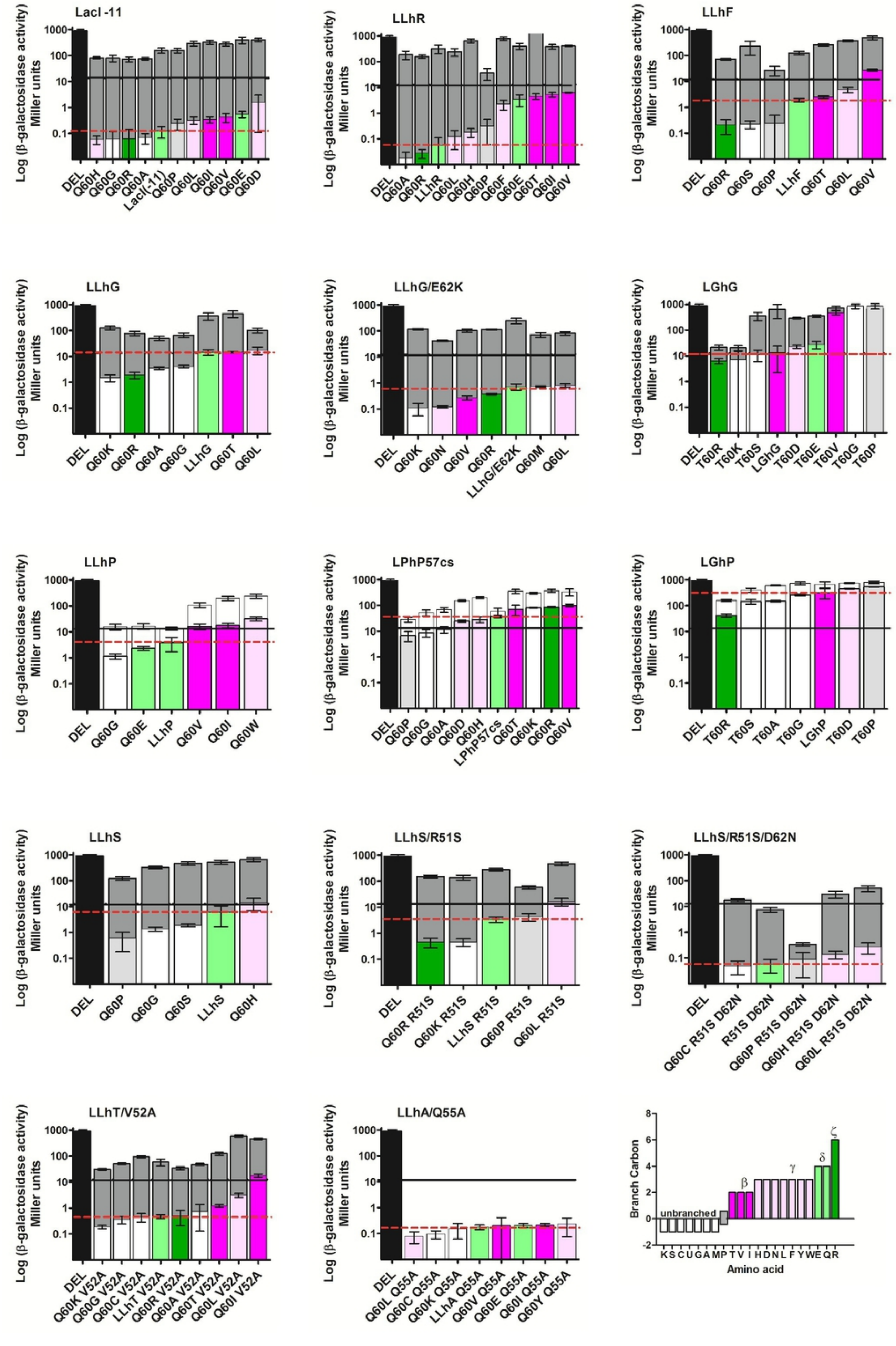

Figure S74: Physicochemical trends: Position 60, Side chain branching

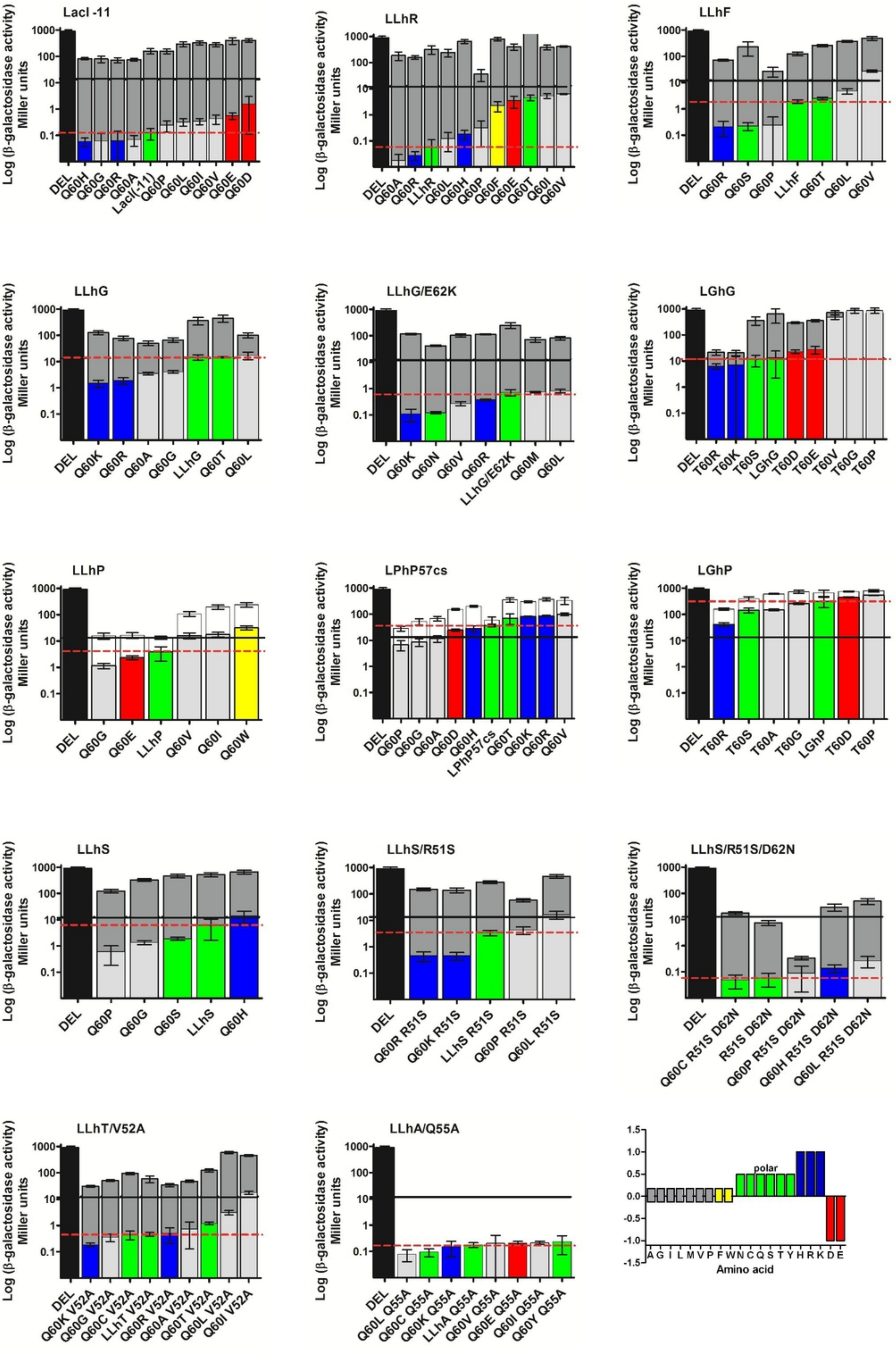

Figure S75: Physicochemical trends: Position 60, Charge/polarity/aromaticity

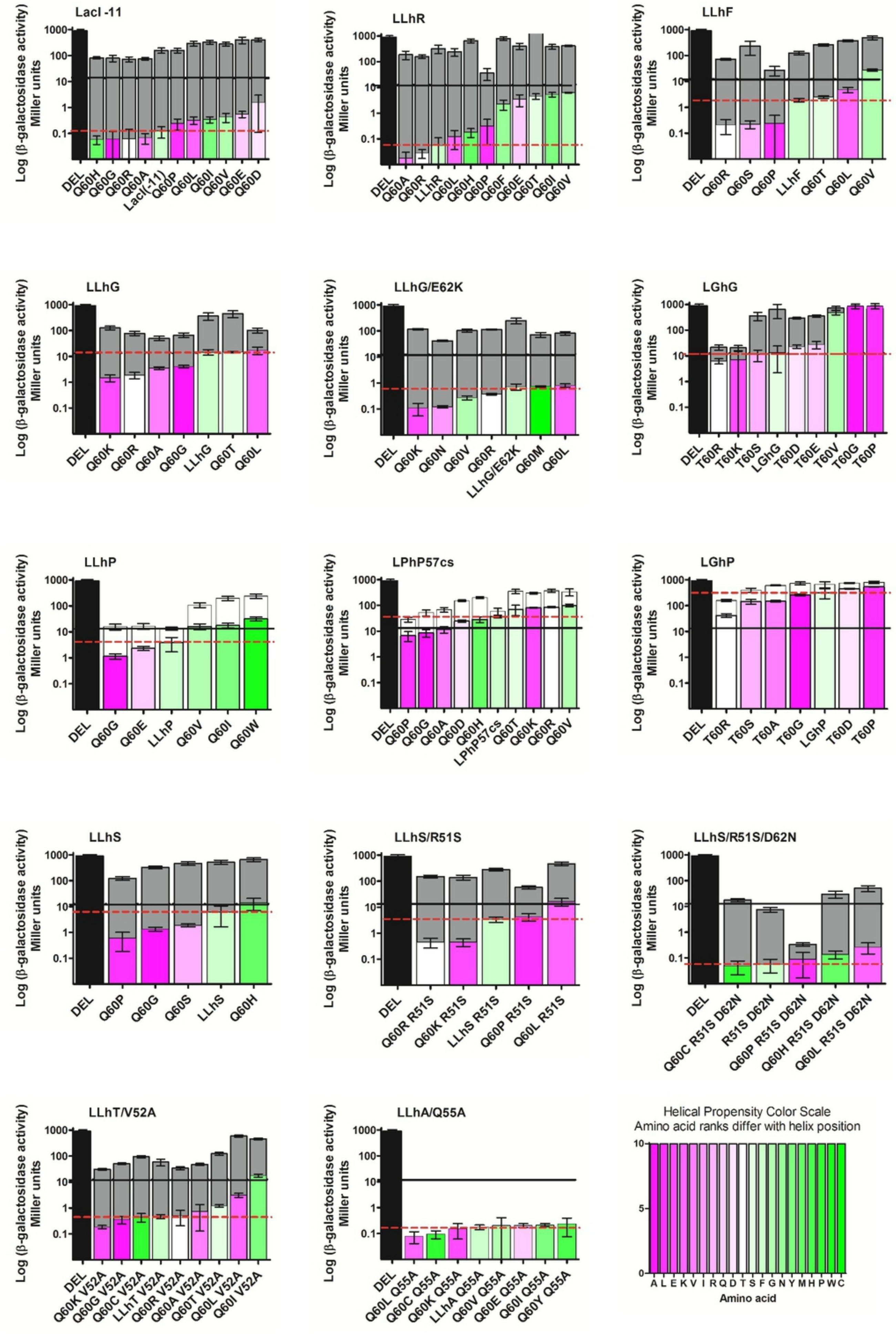

Figure S76: Physicochemical trends: Position 60, Helical propensity (C′ scale)

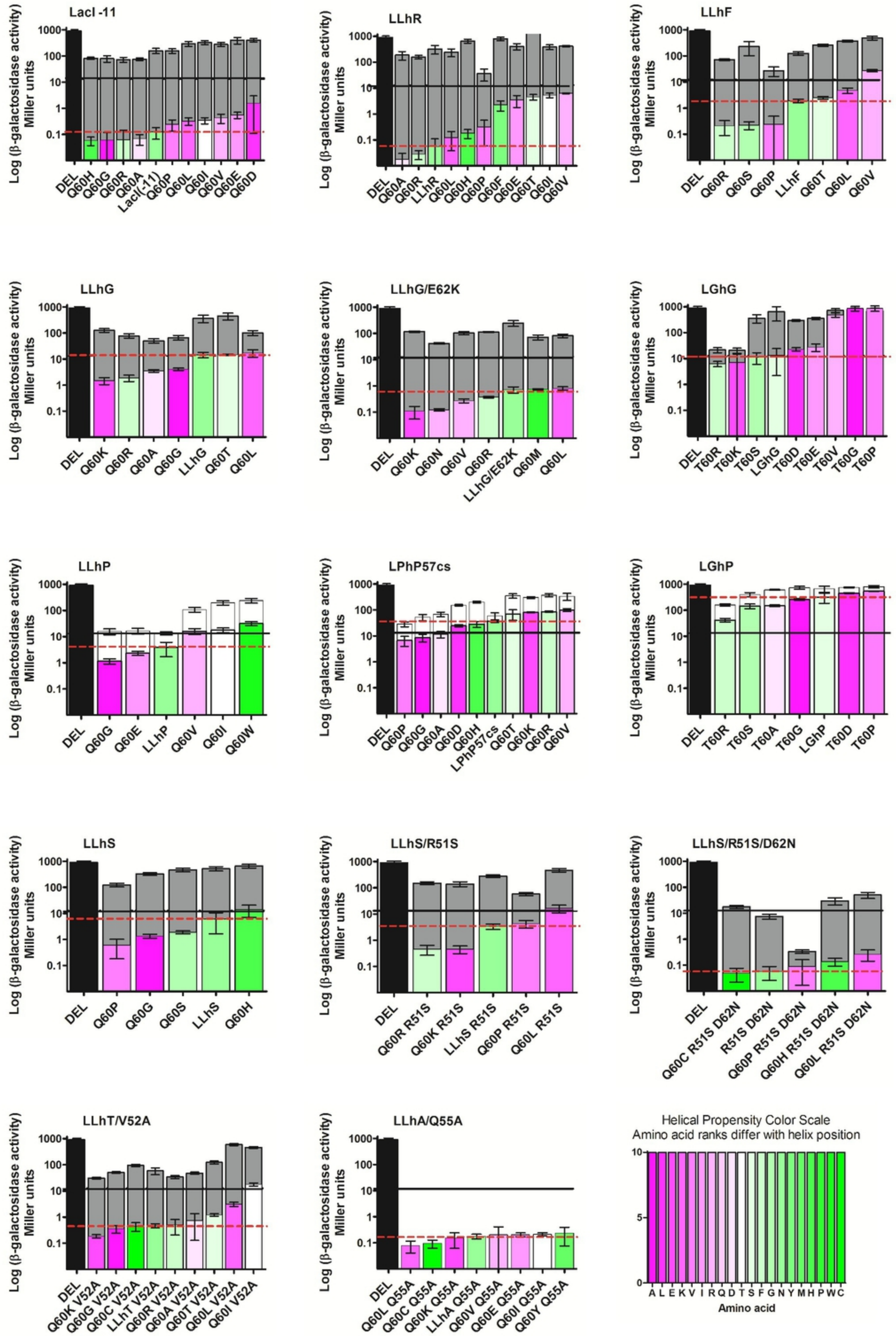

Figure S77: Physicochemical trends: Position 60, Helical propensity (C′′ scale)

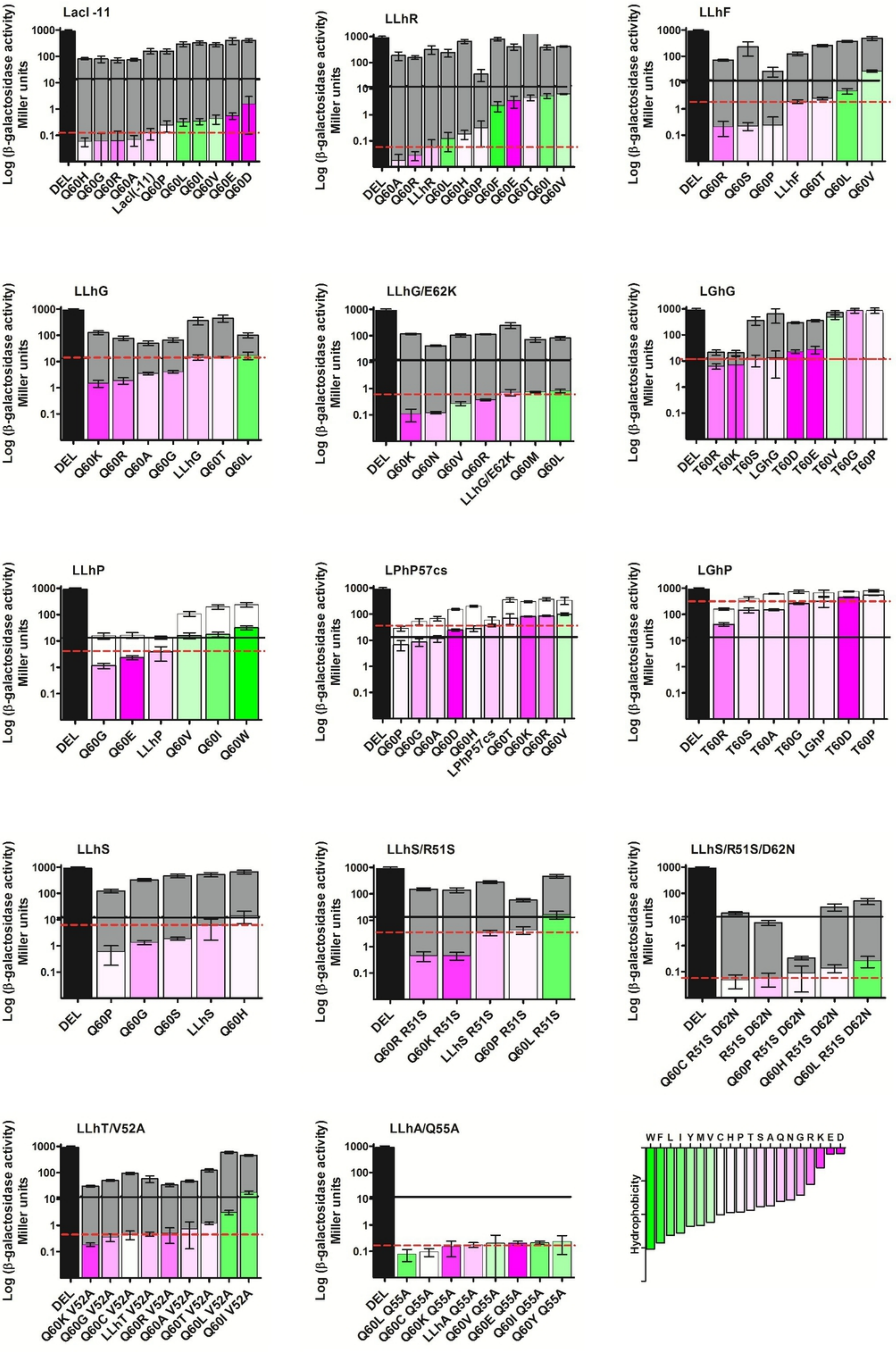

Figure S78: Physicochemical trends: Position 60, Hydrophobicity

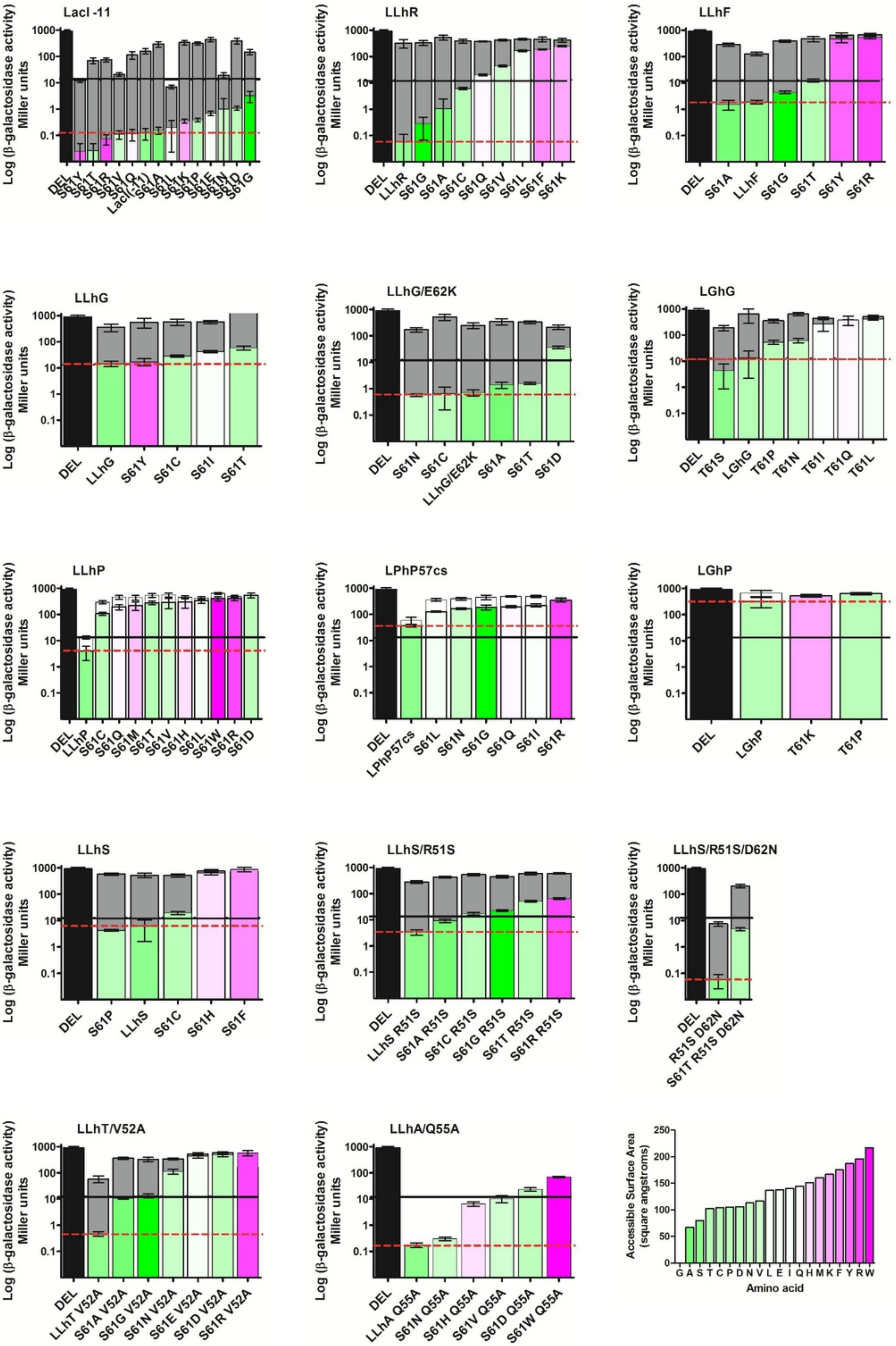

Figure S79: Physicochemical trends: Position 61, Accessible surface area

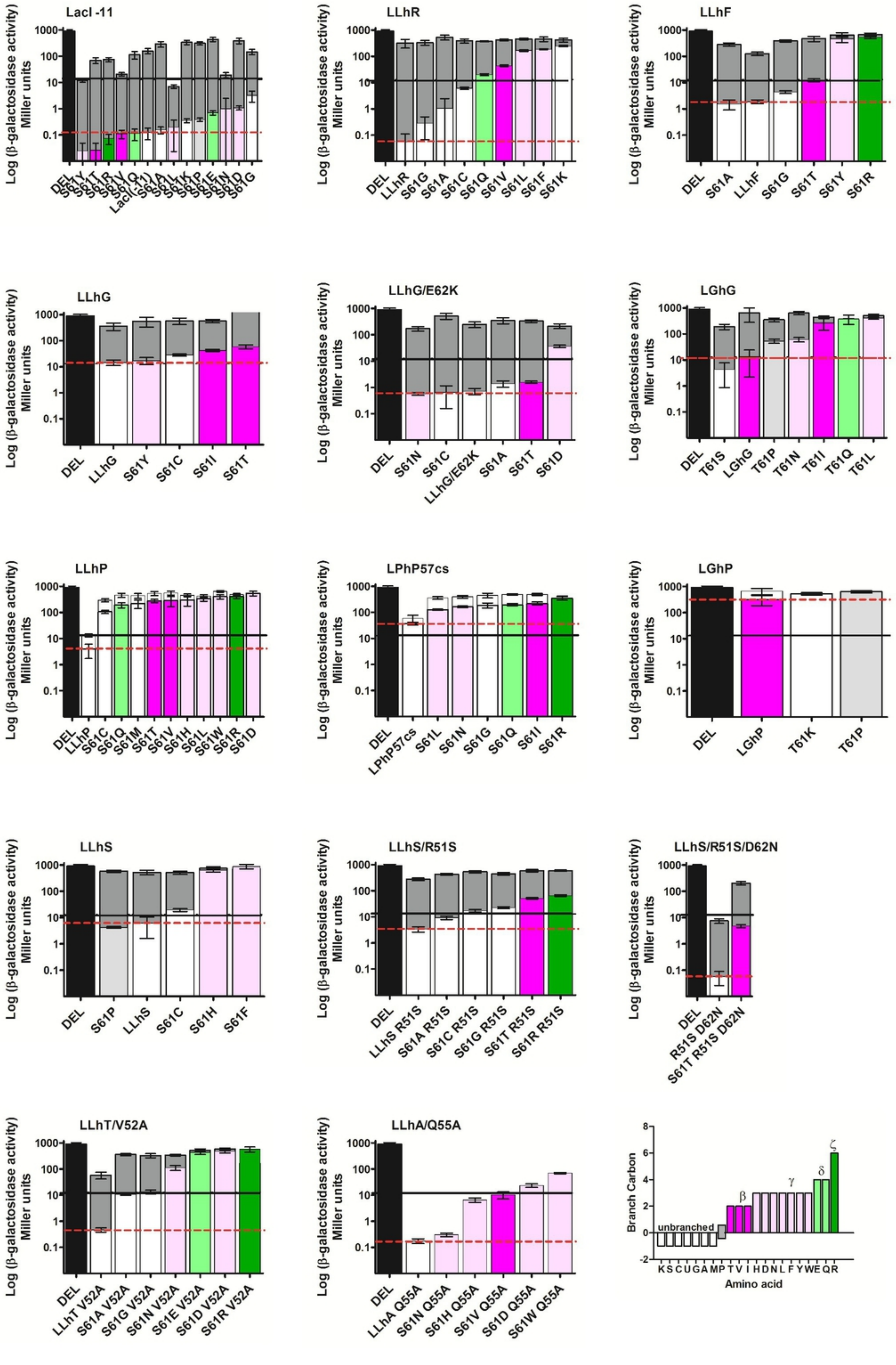

Figure S80: Physicochemical trends: Position 61, Side chain branching

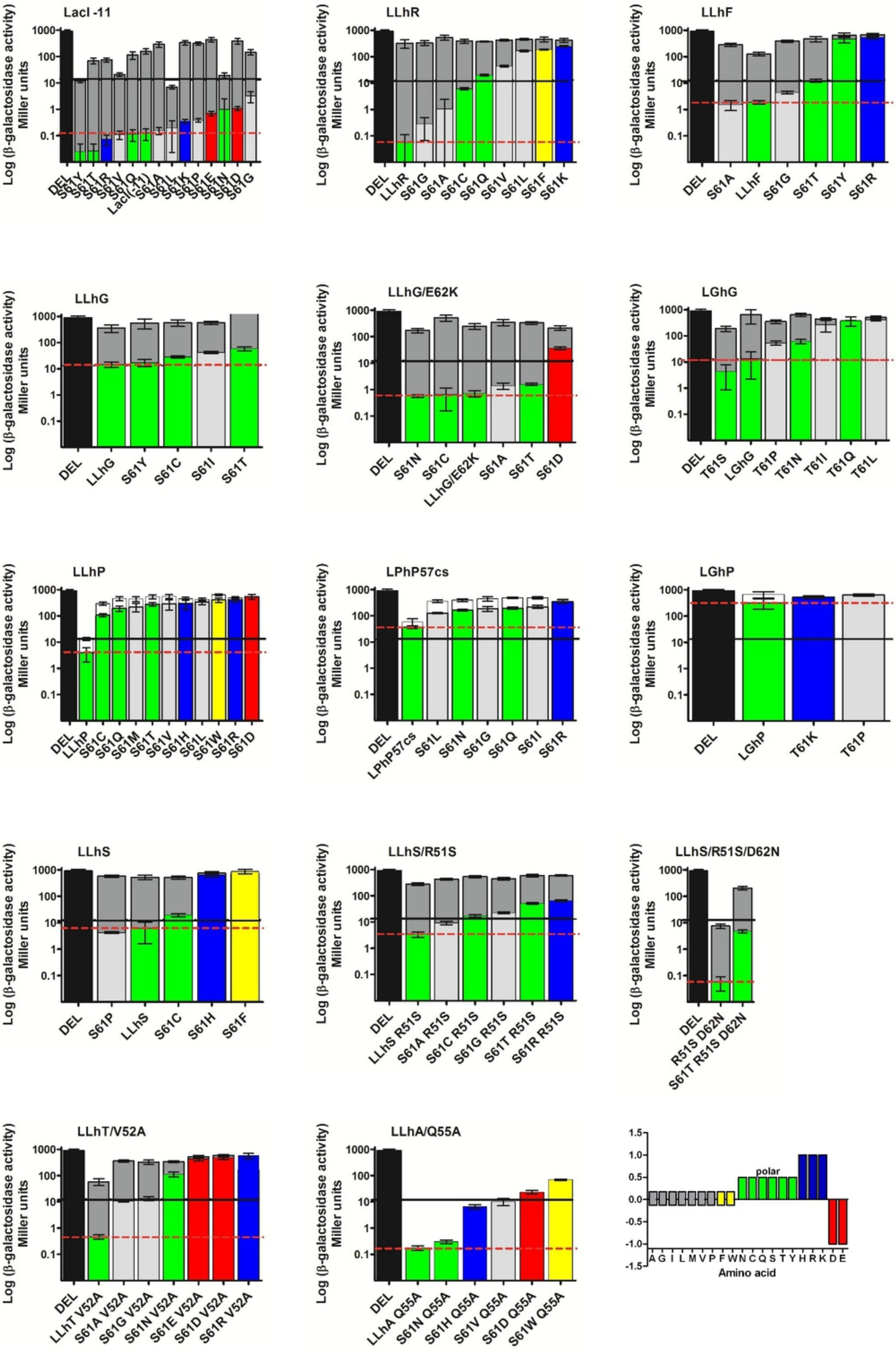

Figure S81: Physicochemical trends: Position 61, Charge/polarity/aromaticity

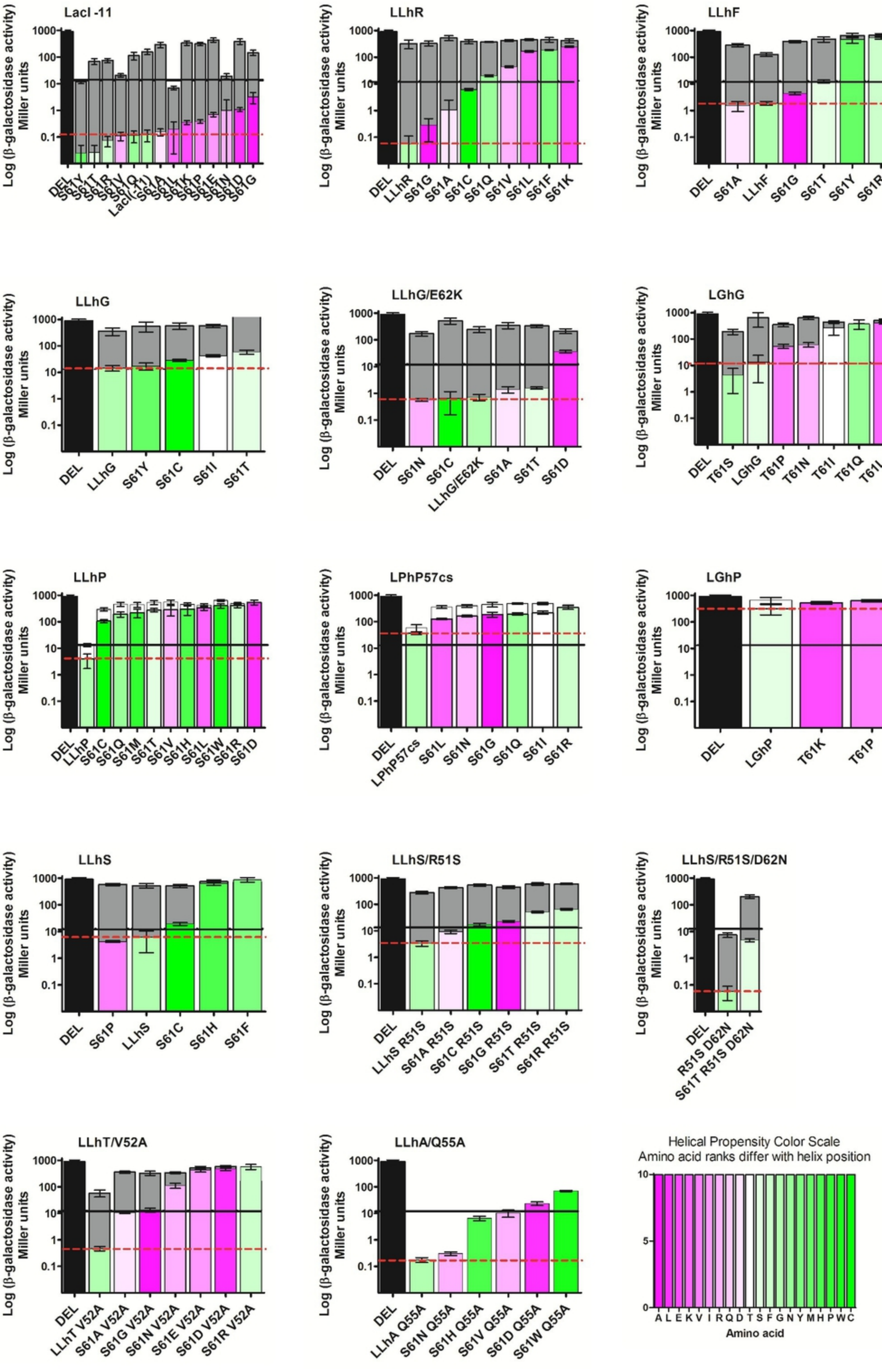

Figure S82: Physicochemical trends: Position 61, Helical propensity (C″ scale)

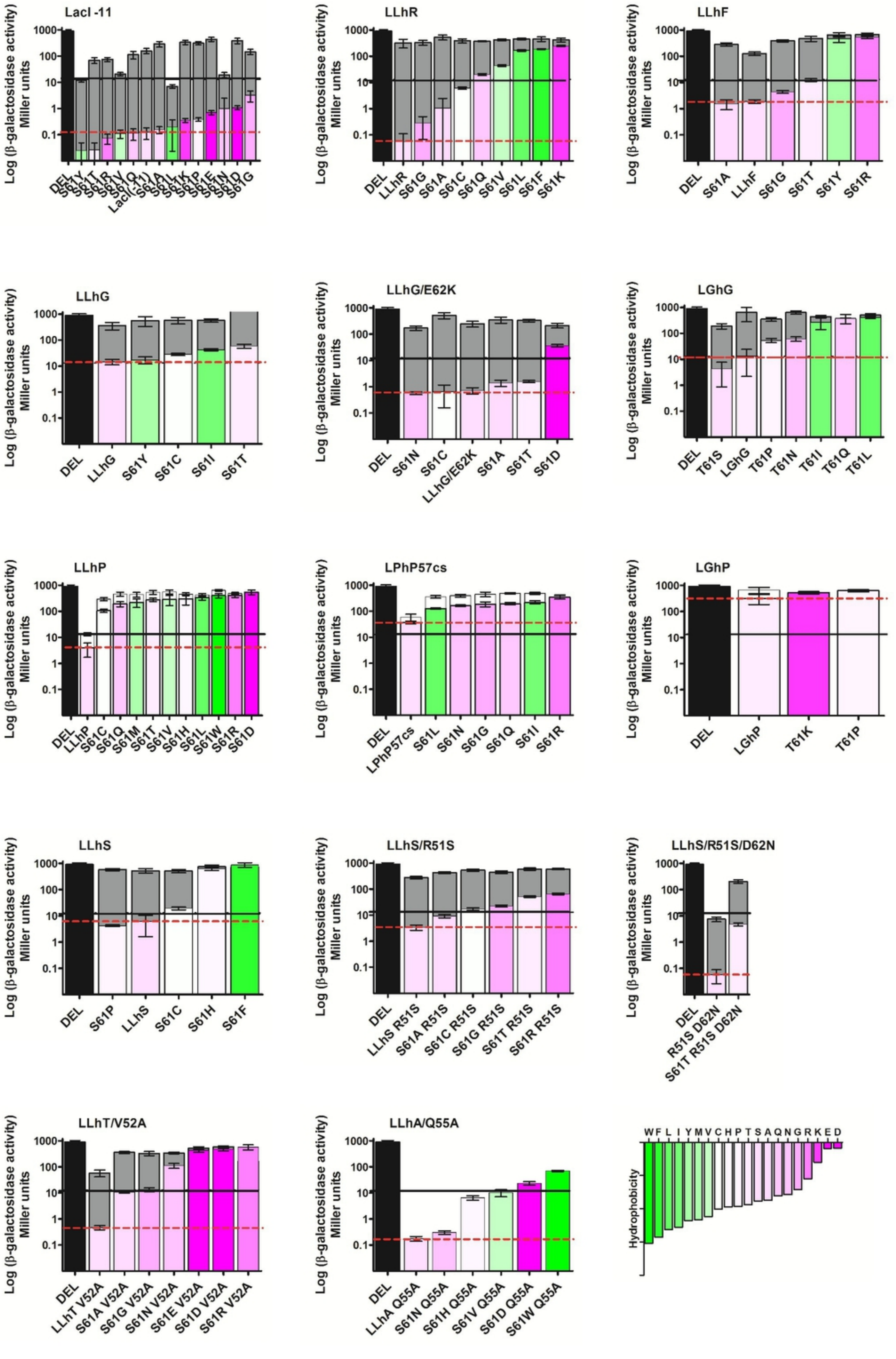

Figure S83: Physicochemical trends: Position 61, Hydrophobicity

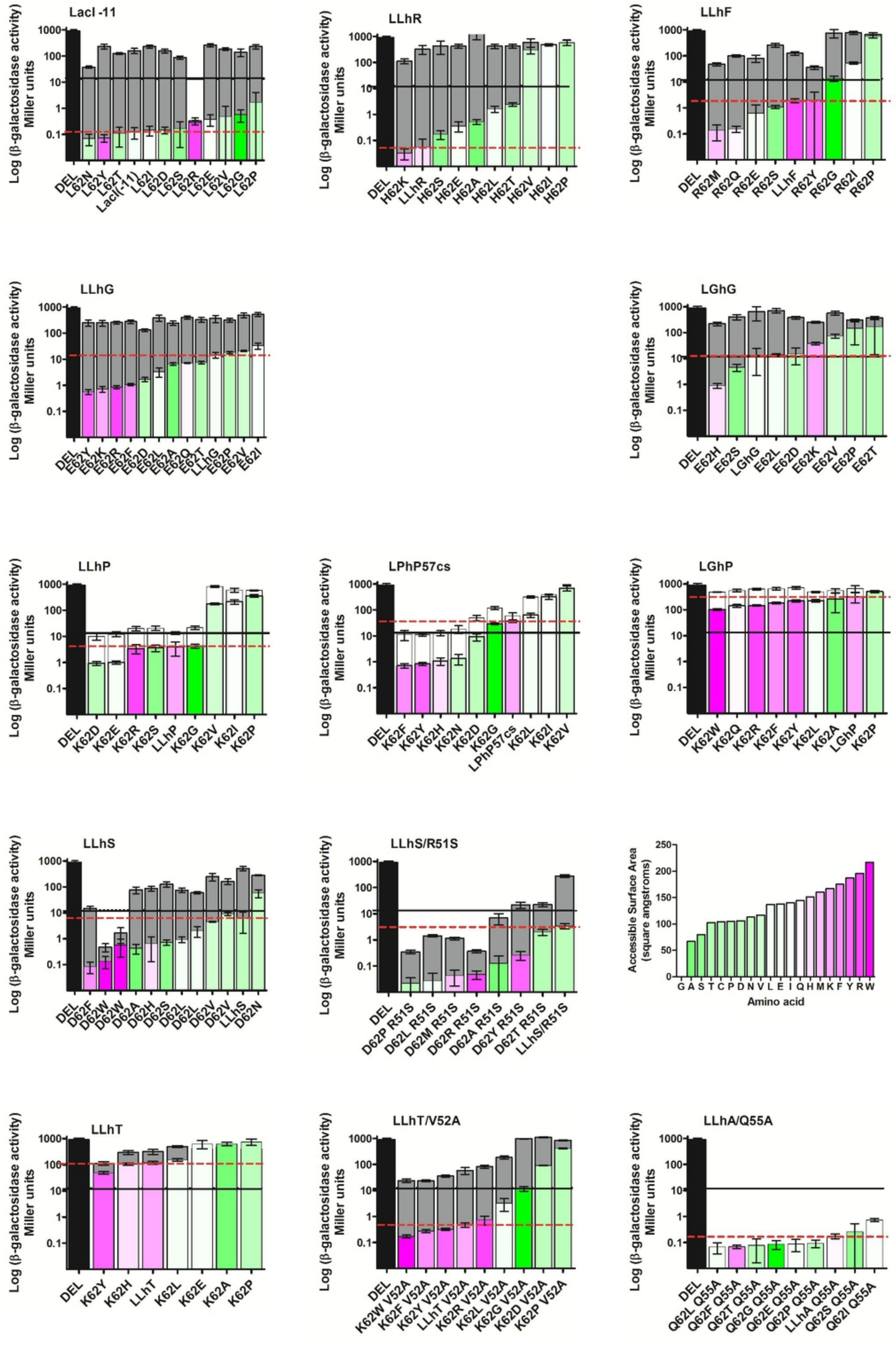

Figure S84: Physicochemical trends: Position 62, Accessible surface area

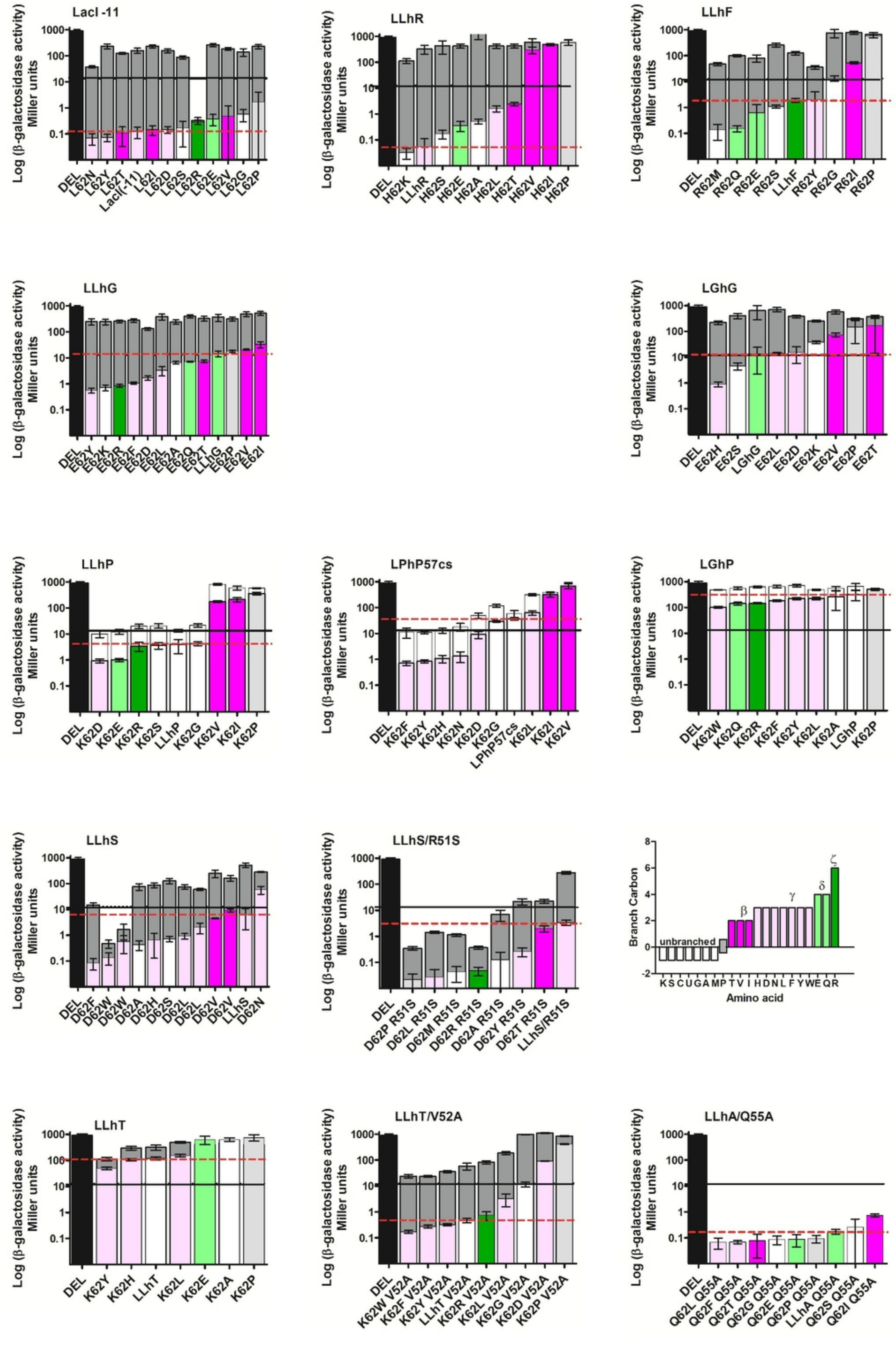

Figure S85: Physicochemical trends: Position 62, Side chain branching

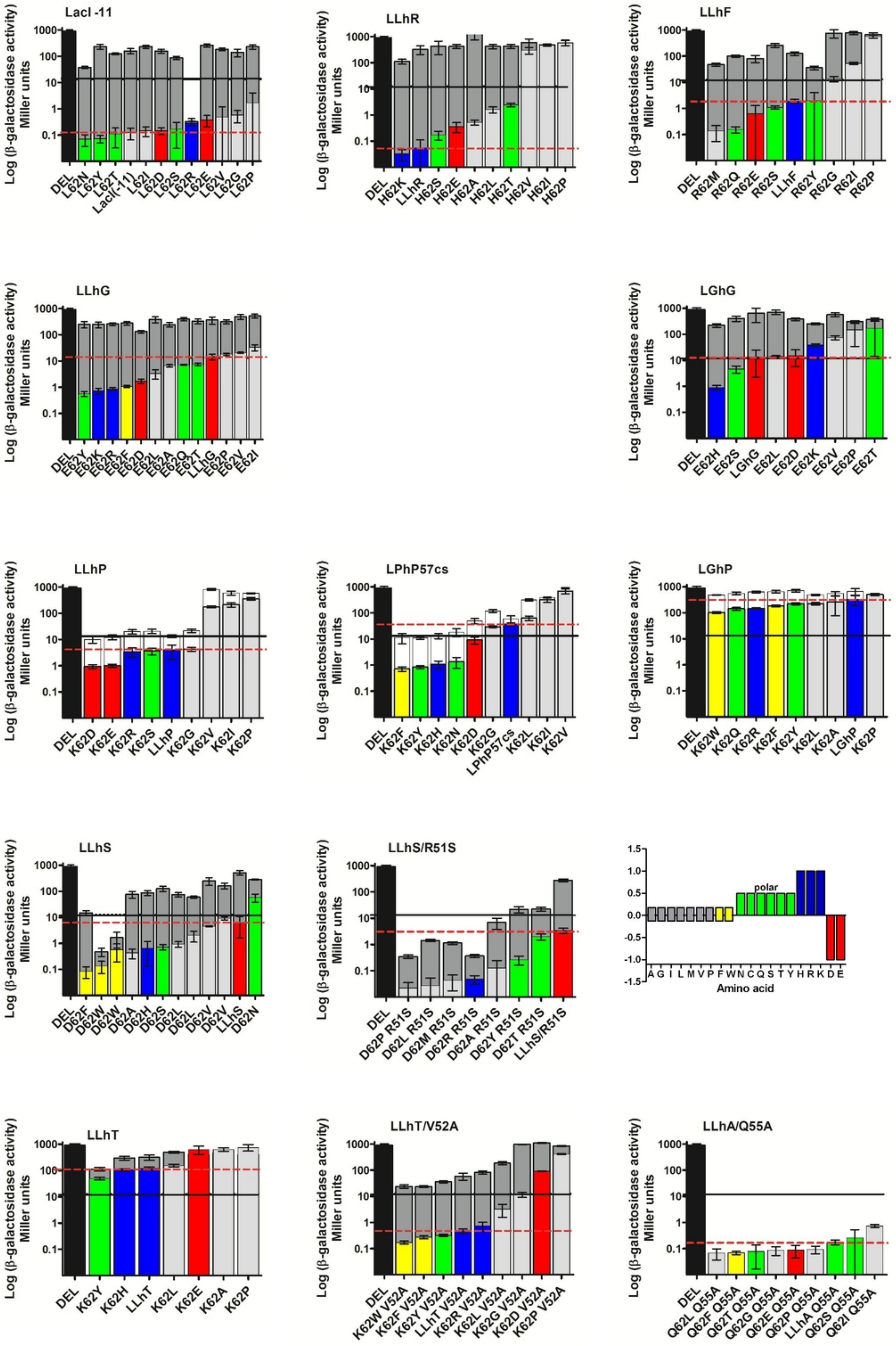

Figure S86: Physicochemical trends: Position 62, Charge/polarity/aromaticity

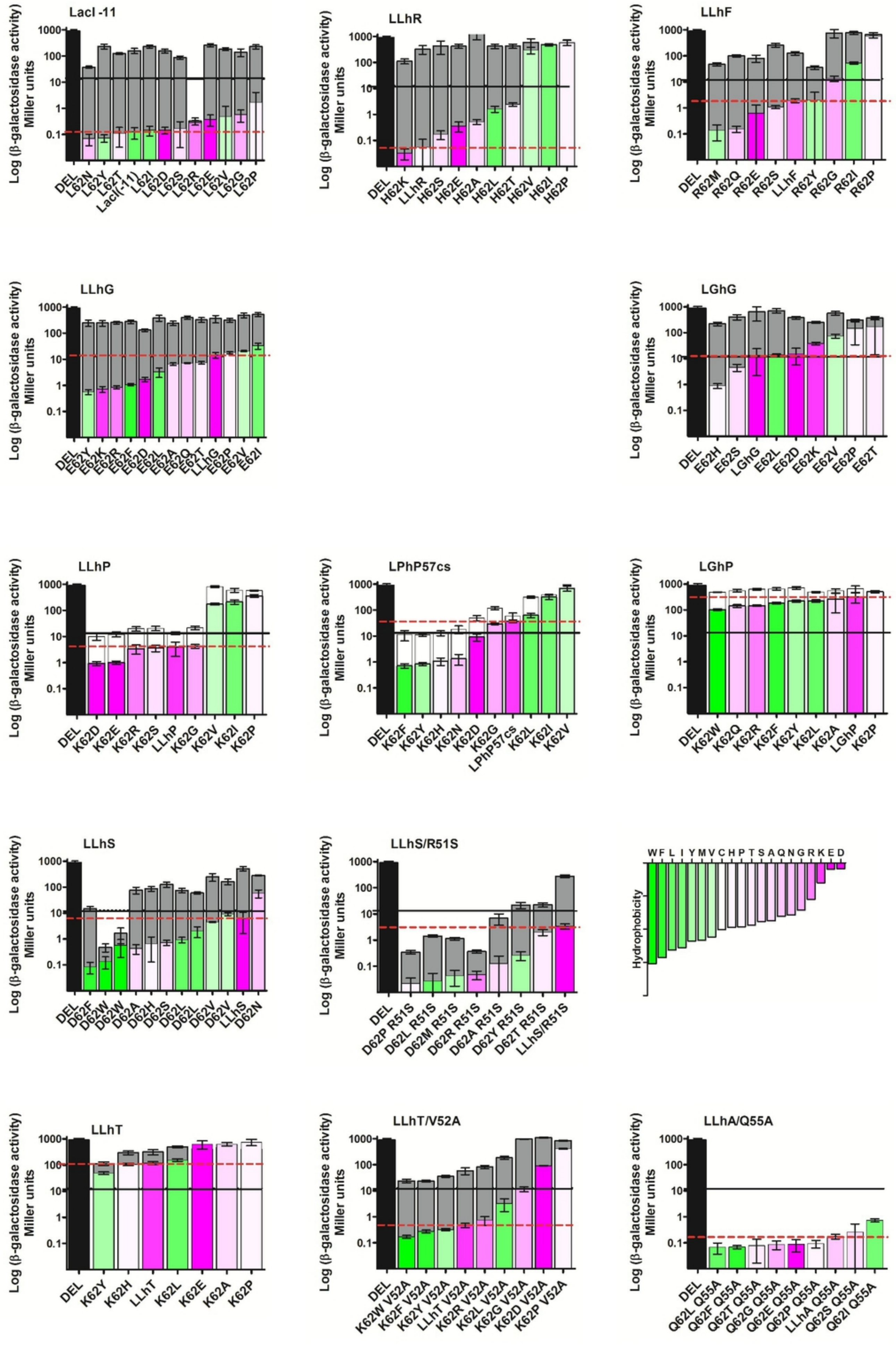

Figure S87: Physicochemical trends: Position 62, Hydrophobicity
